# Association of birth weight with type 2 diabetes mellitus and the mediating role of fatty acids traits: a two-step mendelian randomization study

**DOI:** 10.1186/s12944-024-02087-z

**Published:** 2024-04-02

**Authors:** Limin Cao, Yahui Wen, Keyi Fan, Qiwei Wang, Yaochen Zhang, Zhenglong Li, Nan Wang, Xinhua Zhang

**Affiliations:** 1 Shanxi Children’s Hospital (Shanxi Maternal and Child Health Hospital), Xinmin North Street No.13, Taiyuan, Shanxi China; 2 Shanxi Medical University, Taiyuan, China

**Keywords:** Birth weight, Type-2 diabetes mellitus, Fatty acids, Mediation, Mendelian randomization

## Abstract

**Background:**

Observational studies have suggested an association between birth weight and type 2 diabetes mellitus, but the causality between them has not been established. We aimed to obtain the causal relationship between birth weight with T2DM and quantify the mediating effects of potential modifiable risk factors.

**Methods:**

Two-step, two-sample Mendelian randomization (MR) techniques were applied using SNPs as genetic instruments for exposure and mediators. Summary data from genome-wide association studies (GWAS) for birth weight, T2DM, and a series of fatty acids traits and their ratios were leveraged. The inverse variance weighted (IVW) method was the main analysis approach. In addition, the heterogeneity test, horizontal pleiotropy test, Mendelian randomization pleiotropy residual sum and outlier (MR-PRESSO) test, and leave-one-out analysis were carried out to assess the robustness.

**Results:**

The IVW method showed that lower birth weight raised the risk of T2DM (β: −1.113, 95% CI: −1.573 ∼ −0.652). Two-step MR identified 4 of 17 candidate mediators partially mediating the effect of lower birth weight on T2DM, including ratio of polyunsaturated fatty acids to monounsaturated fatty acids (proportion mediated: 7.9%), ratio of polyunsaturated fatty acids to total fatty acids (7.2%), ratio of omega-6 fatty acids to total fatty acids (8.1%) and ratio of linoleic acid to total fatty acids ratio (6.0%).

**Conclusions:**

Our findings supported a potentially causal effect of birth weight against T2DM with considerable mediation by modifiable risk factors. Interventions that target these factors have the potential to reduce the burden of T2DM attributable to low birth weight.

**Supplementary Information:**

The online version contains supplementary material available at 10.1186/s12944-024-02087-z.

## Introduction

Type 2 diabetes mellitus (T2DM) is a clinical syndrome primarily characterized by a disturbance in glucose metabolism, which comprises a significant burden for public health. According to a recent systematic analysis, the global age-standardised prevalence of T2DM was 5.9% (95% uncertainty interval [UI] 5.5–6.3) in 2021. By 2050, the rate is estimated to reach 9.5% (9.0-9.9), affecting more than 1.27 billion (1.19–1.35) people [1]. T2DM is the result of a complex interaction between genetic and environmental factors, including dietary intake. Identification of potential pathogenic risk factors would help guide the prevention of the disease.

**Fig. 1:**
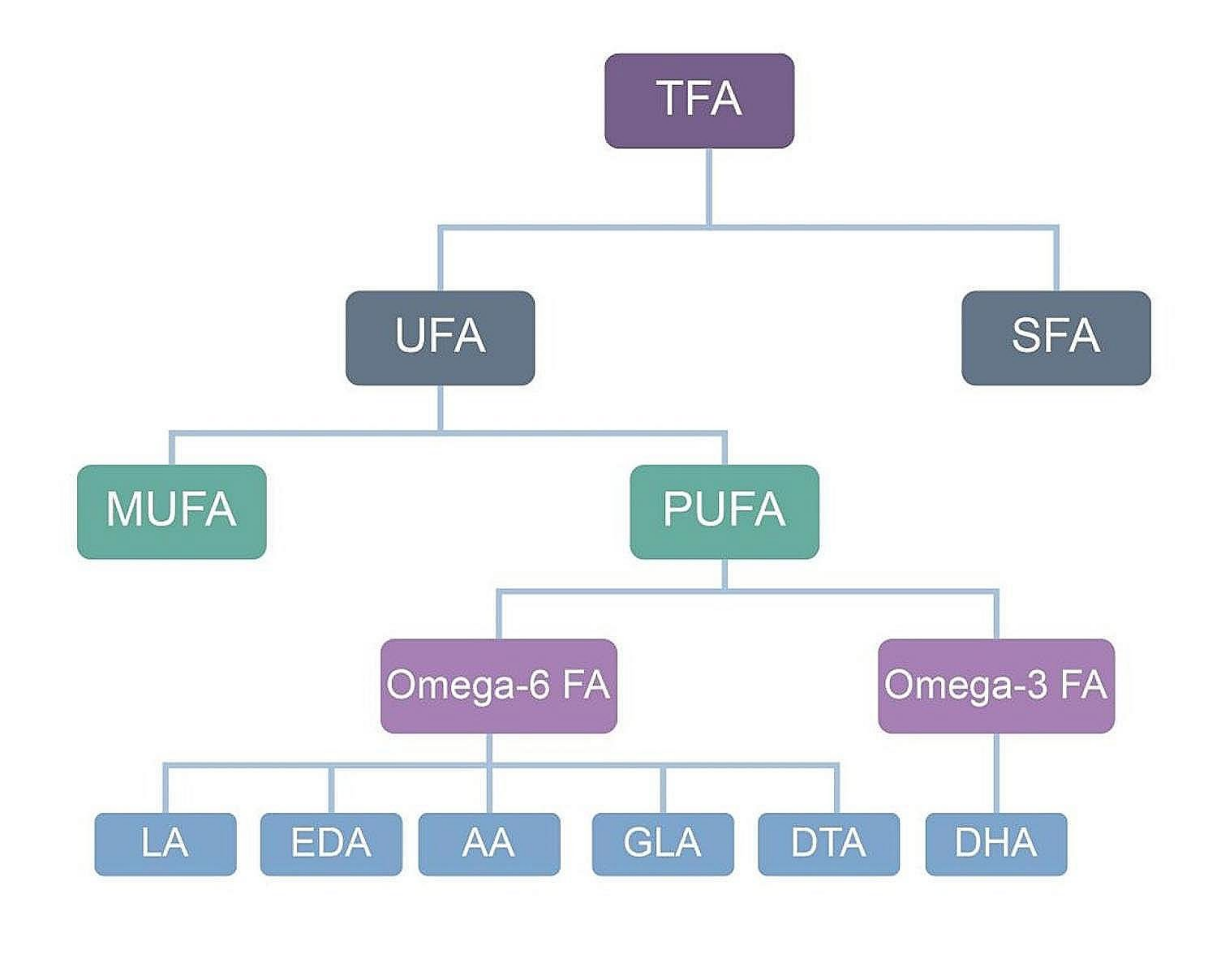
Classification of fatty acids. Abbreviations: AA, arachidonic acid; DHA, docosahexaenoic acid; DTA, docosatetraenoic acid; EDA, eicosadienoic acid; GLA, gamma-linolenic acid; LA, linoleic acid; MUFA, monounsaturated fatty acid; Omega-3 FA, omega-3 fatty acid; Omega-6 FA, omega-6 fatty acid; PUFA, polyunsaturated fatty acid; SFA, saturated fatty acid; TFA, total fatty acid; UFA, unsaturated fatty acid

The fetal development in utero contributes to susceptibility to T2DM, as suggested by the Developmental Origin of Health and Disease (DOHaD) theory. This theory proposes that major risk factors for many adult diseases are established during fertilization, embryonic, fetal, and neonatal stages [2]. Previous studies have indicated that low birth weight is associated with an increased risk of T2DM compared to normal birth weight [3–5]. However, the mechanisms underlying the relationship between birth weight and T2DM remain unclear. Xiaoqiong Zhu et al. [6] demonstrated that fatty acids are risk factors for T2DM. Researchers have reported that fatty acids can regulate gene expression by altering epigenetic mechanisms, leading to either positive or negative outcomes [7, 8]. Based on these facts, we hypothesize that birth weight has a causal relationship with T2DM and glycemic quantitative traits (such as fasting glucose, fasting insulin, HbA1c, and two-hour glucose) through mediating factors (fatty acids traits) (Fig. 1).

Mendelian randomization (MR) is an epidemiological method that uses genetic variants as instrumental variables (IVs) to investigate the causal effects of exposures on disease outcomes [9, 10]. MR is less susceptible to confounding, measurement errors, and reverse causation compared to observational epidemiologic studies because genetic variants are randomly assigned at conception. These strengths also apply to mediation analysis. Previous MR studies suggested a potential causal relationship between birth weight and T2DM. However, these studies did not assess the potential mediators [11–13]. Therefore, we conducted an MR analysis to assess the impact of birth weight on T2DM and quantify the role of fatty acids traits as mediators.

## Methods

### Study design

**Fig. 2:**
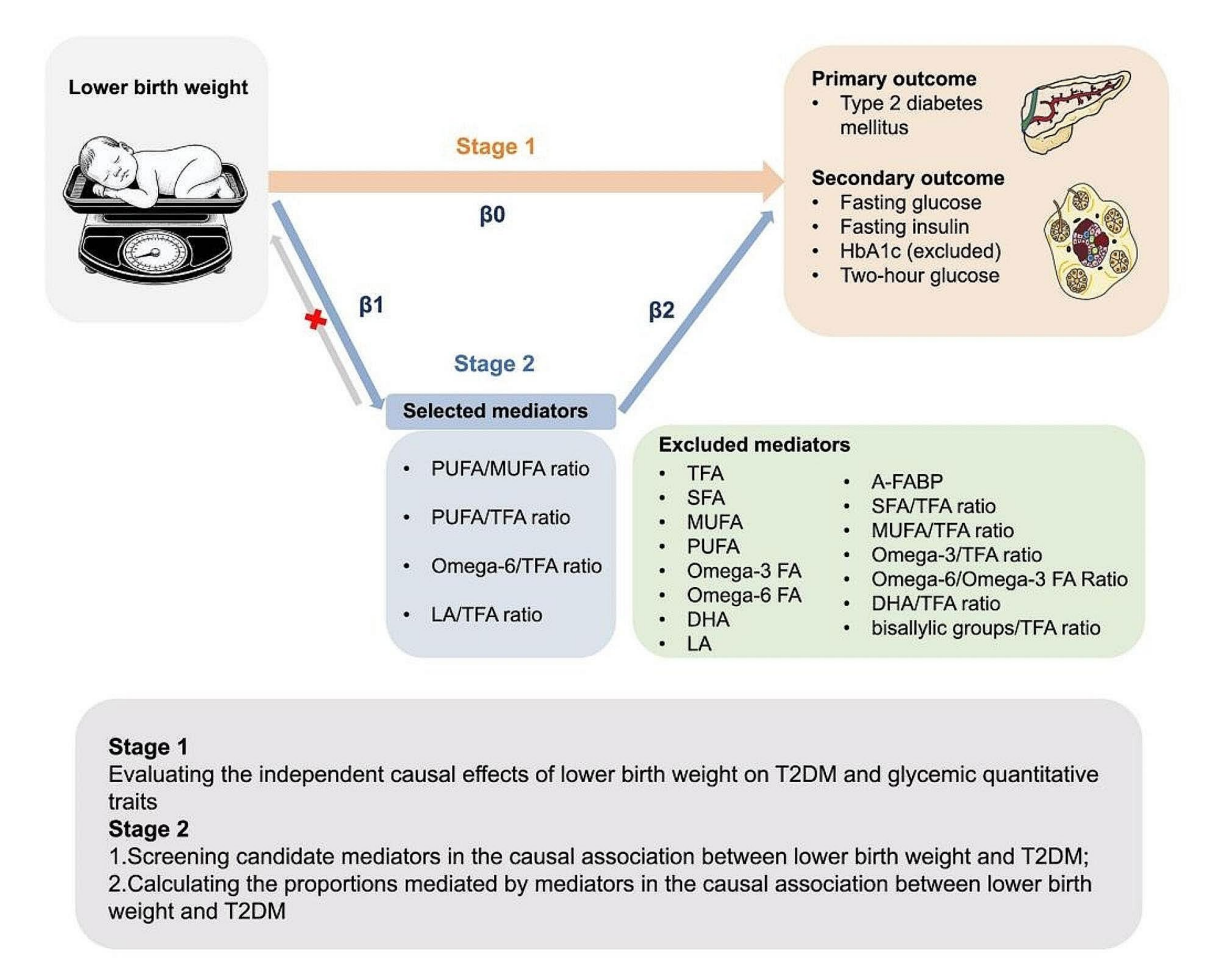
Overview of the MR study design. Abbreviations A-FABP, fatty acid-binding protein, adipocyte levels; bisallylic groups/TFA ratio, ratio of bisallylic groups to total fatty acids; DHA, docosahexaenoic acid levels; DHA/TFA ratio, ratio of docosahexaenoic acid to total fatty acid levels; LA, linoleic acid levels; LA/TFA ratio, ratio of linoleic acid to total fatty acids; MUFA, monounsaturated fatty acid levels; MUFA/TFA ratio, ratio of monounsaturated fatty acids to total fatty acids; Omega-3 FA, omega-3 fatty acid levels; Omega-3/TFA ratio, ratio of omega-3 fatty acids to total fatty acids; Omega-6 FA, omega-6 fatty acid levels; Omega-6/Omega-3 FA ratio, ratio of omega-6 fatty acids to omega-3 fatty acids; Omega-6/TFA ratio, ratio of omega-6 fatty acids to total fatty acids; PUFA, polyunsaturated fatty acid levels; PUFA/MUFA ratio, ratio of polyunsaturated fatty acids to monounsaturated fatty acids; PUFA/TFA ratio, ratio of polyunsaturated fatty acids to total fatty acids; SFA, saturated fatty acid levels; SFA/TFA ratio, ratio of saturated fatty acids to total fatty acids; TFA, total fatty acid levels

We reported the MR study in adherence to the Strengthening the Reporting of Observational Studies in Epidemiology using Mendelian Randomization (STROBE-MR). In MR analysis, we extracted single nucleotide polymorphisms (SNPs) from the GWAS database as genetic IVs to clarify the causal association between risk factors and outcomes. MR analysis can yield valid causal estimates if the following assumptions are met: [1] the IVs must be significantly closely related to exposures; [2] the IVs must be free of confounders; and [3] the IVs must be unrelated to the outcomes and only affect the outcomes through the exposure (Fig. S1). Two-sample MR analyses were first performed to assess the associations between birth weight and T2DM, and two-step MR analysis was then performed to investigate the mediating effects of fatty acids traits on these associations (Fig. 2).

Ethics committee approval and participant informed consent were obtained in the original studies.

### Data sources

SNPs associated with birth weight were obtained from UK Biobank, which contained 261,932 participants. For each birth weight instrument, the genetic effect of the corresponding SNP on T2DM was obtained from a GWAS study with a total of 298,957 Europeans (48,286 diabetes cases and 250,671 controls) [14]. Glycemic quantitative traits (fasting glucose, fasting insulin, HbA1c, and two-hour glucose) were also collected as secondary outcomes. Finally, the summary statistics for intermediate phenotypes (fatty acids) were selected.

All the GWAS summary data applied in the present analysis can be obtained from the IEU open GWAS project (https://gwas.mrcieu.ac.uk/). The above GWAS data are from the European origin population, and their information is shown in Table 1.

**Table 1:** Summary of the GWAS data used in the MR analyses

	GWAS ID	Trait	Year	Population	Sample Size	Number of SNPs	Author
Exposure	ukb-b-13,378	Birth weight	2018	European	261,932	9,851,867	Ben Elsworth
Mediator	ebi-a-GCST90092987	TFA	2022	European	115,006	11,590,399	Tom G Richardson
Mediator	ebi-a-GCST90092980	SFA	2022	European	115,006	11,590,399	Tom G Richardson
Mediator	ebi-a-GCST90092928	MUFA	2022	European	115,006	11,590,399	Tom G Richardson
Mediator	ebi-a-GCST90092939	PUFA	2022	European	115,006	11,590,399	Tom G Richardson
Mediator	ebi-a-GCST90092931	Omega-3 FA	2022	European	115,006	11,590,399	Tom G Richardson
Mediator	ebi-a-GCST90092933	Omega-6 FA	2022	European	115,006	11,590,399	Tom G Richardson
Mediator	ebi-a-GCST90092816	DHA	2020	European	115,006	11,590,399	Tom G Richardson
Mediator	ebi-a-GCST90092880	LA	2021	European	115,006	11,590,399	Tom G Richardson
Mediator	ebi-a-GCST90012075	A-FABP	2020	European	21,758	13,138,563	Lasse Folkersen
Mediator	ebi-a-GCST90092981	SFA/TFA ratio	2022	European	115,006	11,590,399	Tom G Richardson
Mediator	ebi-a-GCST90092929	MUFA/TFA ratio	2022	European	115,006	11,590,399	Tom G Richardson
Mediator	ebi-a-GCST90092940	PUFA/MUFA ratio	2022	European	115,006	11,590,399	Tom G Richardson
Mediator	ebi-a-GCST90092941	PUFA/TFA ratio	2022	European	115,006	11,590,399	Tom G Richardson
Mediator	ebi-a-GCST90092932	Omega-3/TFA ratio	2022	European	115,006	11,590,399	Tom G Richardson
Mediator	ebi-a-GCST90092934	Omega-6/Omega-3 FA Ratio	2022	European	115,006	11,590,399	Tom G Richardson
Mediator	ebi-a-GCST90092935	Omega-6/TFA ratio	2022	European	115,006	11,590,399	Tom G Richardson
Mediator	ebi-a-GCST90092817	DHA/TFA ratio	2022	European	115,006	11,590,399	Tom G Richardson
Mediator	ebi-a-GCST90092881	LA/TFA ratio	2022	European	115,006	11,590,399	Tom G Richardson
Mediator	met-c-845	bisallylic groups/TFA ratio	2016	European	13,171	11,274,684	Johannes Kettunen
Outcome	ebi-a-GCST007516	Type 2 diabetes (adjusted for BMI)	2018	European	298,957	190,208	Anubha Mahajan
Outcome	ebi-a-GCST90002232	Fasting glucose	2021	European	200,622	31,008,728	Ji Chen
Outcome	ebi-a-GCST90002238	Fasting insulin	2021	European	151,013	29,664,438	Ji Chen
Outcome	ieu-b-4842	HbA1c	2022	European	45,734	9,696,819	Howe LJ
Outcome	ebi-a-GCST90002227	Two-hour glucose	2021	European	63,396	27,330,879	Ji Chen

*Abbreviations* A-FABP, fatty acid-binding protein, adipocyte levels; bisallylic groups/TFA ratio, ratio of bisallylic groups to total fatty acids; DHA, docosahexaenoic acid levels; DHA/TFA ratio, ratio of docosahexaenoic acid to total fatty acid levels; GWAS, genome-wide association study; LA, linoleic acid levels; LA/TFA ratio, ratio of linoleic acid to total fatty acids; MUFA, monounsaturated fatty acid levels; MUFA/TFA ratio, ratio of monounsaturated fatty acids to total fatty acids; Omega-3 FA, omega-3 fatty acid levels; Omega-3/TFA ratio, ratio of omega-3 fatty acids to total fatty acids; Omega-6 FA, omega-6 fatty acid levels; Omega-6/Omega-3 FA ratio, ratio of omega-6 fatty acids to omega-3 fatty acids; Omega-6/TFA ratio, ratio of omega-6 fatty acids to total fatty acids; PUFA, polyunsaturated fatty acid levels; PUFA/MUFA ratio, ratio of polyunsaturated fatty acids to monounsaturated fatty acids; PUFA/TFA ratio, ratio of polyunsaturated fatty acids to total fatty acids; SFA, saturated fatty acid levels; SFA/TFA ratio, ratio of saturated fatty acids to total fatty acids; TFA, total fatty acid levels

### Instrumental variable selection and data harmonization

For all analyses, genetic instruments with p-value < 5 × 10^− 8^ were defined as SNPs. In addition, we selected independent SNPs according to the removal of linkage disequilibrium (LD, R^2^ > 0.001 and within 10,000 kb). We calculated the F-statistic to quantify the strength of associations between genetic IVs and exposure, and discarded those with an F statistic < 10. The F statistic was calculated using the following formula: F = R^2^ (N − K − 1)/(K (1 − R^2^). R^2^ was calculated using the formula R^2^ = (2 × EAF × (1 − EAF) × β^2^)/[(2 × EAF × (1 − EAF) × β^2^) + (2 × EAF × (1 − EAF) × N × SE^2^)], where R^2^, N, EAF, β, SE, and K refer to the cumulative explained variance of selected SNPs in the exposure, the sample size, the effect allele frequency, the estimated effect on the exposure, the standard error of the estimated effect, and the number of IVs, respectively.

### MR analyses and mediation analysis

We used the inverse variance weighted (IVW) method as the principal MR analytical approach; due to its high statistical power when the selected IVs were valid [15]. The MR-Egger [16], weighted median method, simple mode, weighted mode, and robust adjusted profile score (RAPS) were used in the two-sample MR analyses as complementary approaches to obtain MR estimates.

Two-sample MR was conducted to estimate the overall effect of birth weight on diabetes (β0) and the effect of birth weight on each mediator (β1) using publicly available GWAS. We further carried out multivariable MR (MVMR) to assess the effect of each mediator on diabetes (β2), adjusting for birth weight.

The total effect of birth weight was decomposed into direct (not acting through the mediators) and indirect (acting through the mediators) effects. The indirect effect of each mediator was calculated by the product of coefficients method (β1*β2) [17]. And the direct effects were estimated by subtracting the indirect effect of birth weight from the total effect. Then we calculated the proportion of the mediated as indirect effect divided by total effect. The 95% confidence intervals were calculated using the delta method.

### Sensitivity analyses

To evaluate the robustness of the findings, several sensitivity analyses were conducted. Firstly, the presence of heterogeneity was evaluated using Cochran’s Q statistic (MR-IVW) and Rucker’s Q statistic (MR Egger). Secondly, we used MR-Egger regression intercept test to assess the horizontal pleiotropy of valid IVs. Thirdly, the MR-PRESSO method detected and excluded for outliers, thereby eliminating detected pleiotropy. Finally, a leave-one-out (LOO) sensitivity analysis was conducted by sequentially omitting one SNP at a time to evaluate the possibility of results being driven by a single SNP [18].

The adjusted p-value threshold was set to 0.05 using the Benjamini-Hochberg (BH) method [19]. All statistical analyses were conducted using the TwoSampleMR (version 0.5.8, https://mrcieu.github.io/TwoSampleMR) and MRPRESSO (version 1.0) packages in R (version 4.2.2).

## Results

### Total effect of birth weight on T2DM and glycemic quantitative traits

**Fig. 3:**
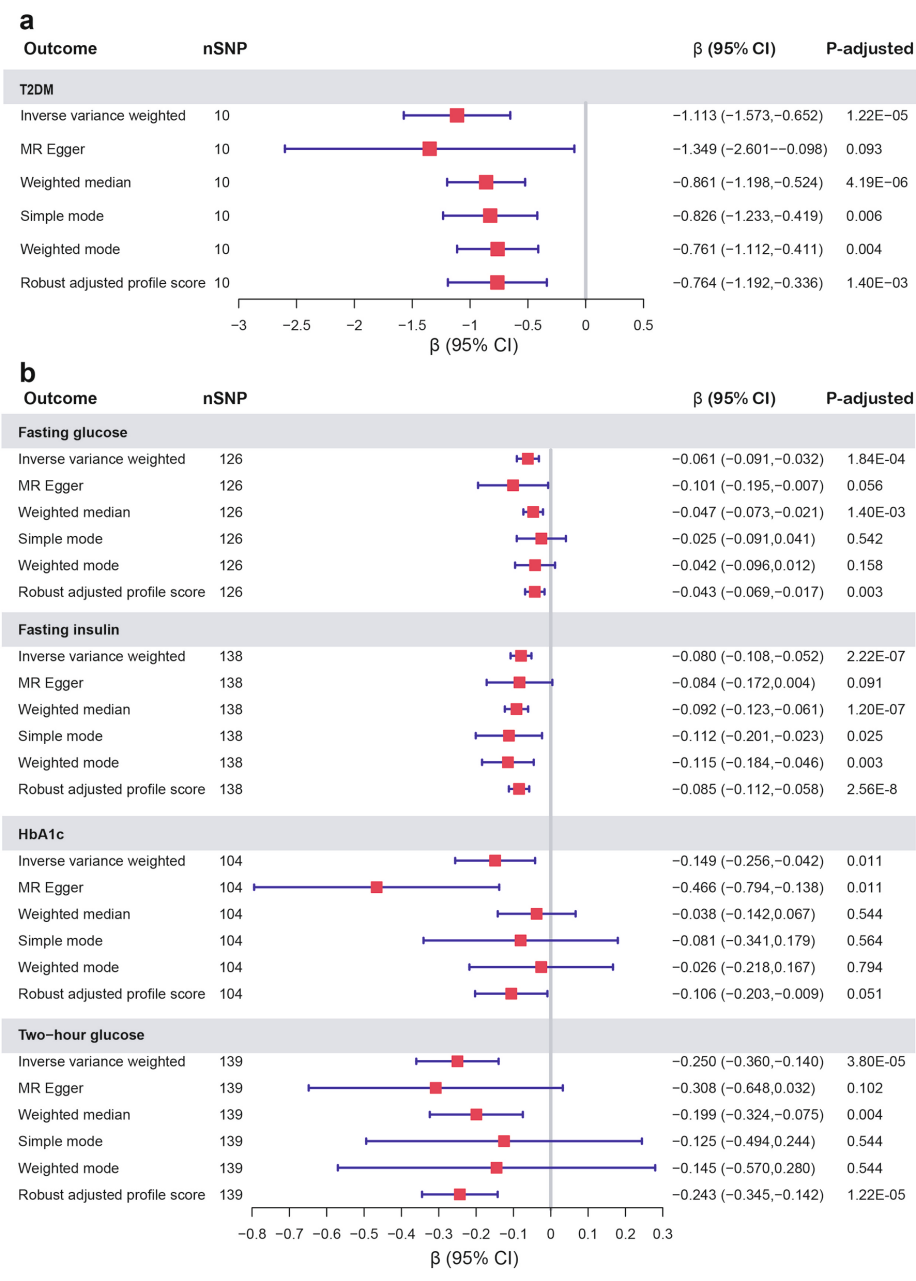
**a** Estimates of the causal effect of birth weight on T2DM using different MR methods. **b** Estimates of the causal effect of birth weight on fasting glucose, fasting insulin, HbA1c, and two-hour glucose using different MR methods. OR, odds ratio; CI, confidence interval

We found strong evidence of a causal relationship between birth weight and T2DM. Figure 3a illustrates the overall effect of birth weight on T2DM, fasting glucose, fasting insulin, HbA1c, and two-hour glucose. The IVW method showed that lower birth weight was associated with a higher risk of T2DM (β: −1.113, 95% CI: −1.573 ∼ −0.652), higher fasting insulin (β: −0.080, −0.108 ∼ −0.052) and higher two-hour glucose (β: −0.250, −0.360 ∼ −0.140).

Horizontal pleiotropy was detected for fasting glucose (ER intercept = 0.003; *P* = 0.028) and HbA1c (ER intercept = 0.008; *P* = 0.038). Potentially pleiotropic SNPs were excluded using MR-PRESSO. After removing 13 outliers (rs7854962, rs76895963, rs74932341, rs351776, rs329121, rs2934844, rs28378473, rs2747503, rs2159778, rs1522811, rs13266210, rs11708067 and rs10769199), we found that a lower birth weight indicated higher fasting glucose (β: −0.061, −0.091 ∼ −0.032), and no horizontal pleiotropy was detected (ER intercept = 0.001; *P* = 0.384). However, ER still indicated significant pleiotropy (ER intercept = 0.008; *P* = 0.048) for HbA1c after removing rs72790949, rs2551402, rs2395668, rs1776270, rs11222084(Table S14).

### Effect of birth weight on fatty acids traits

**Fig. 4:**
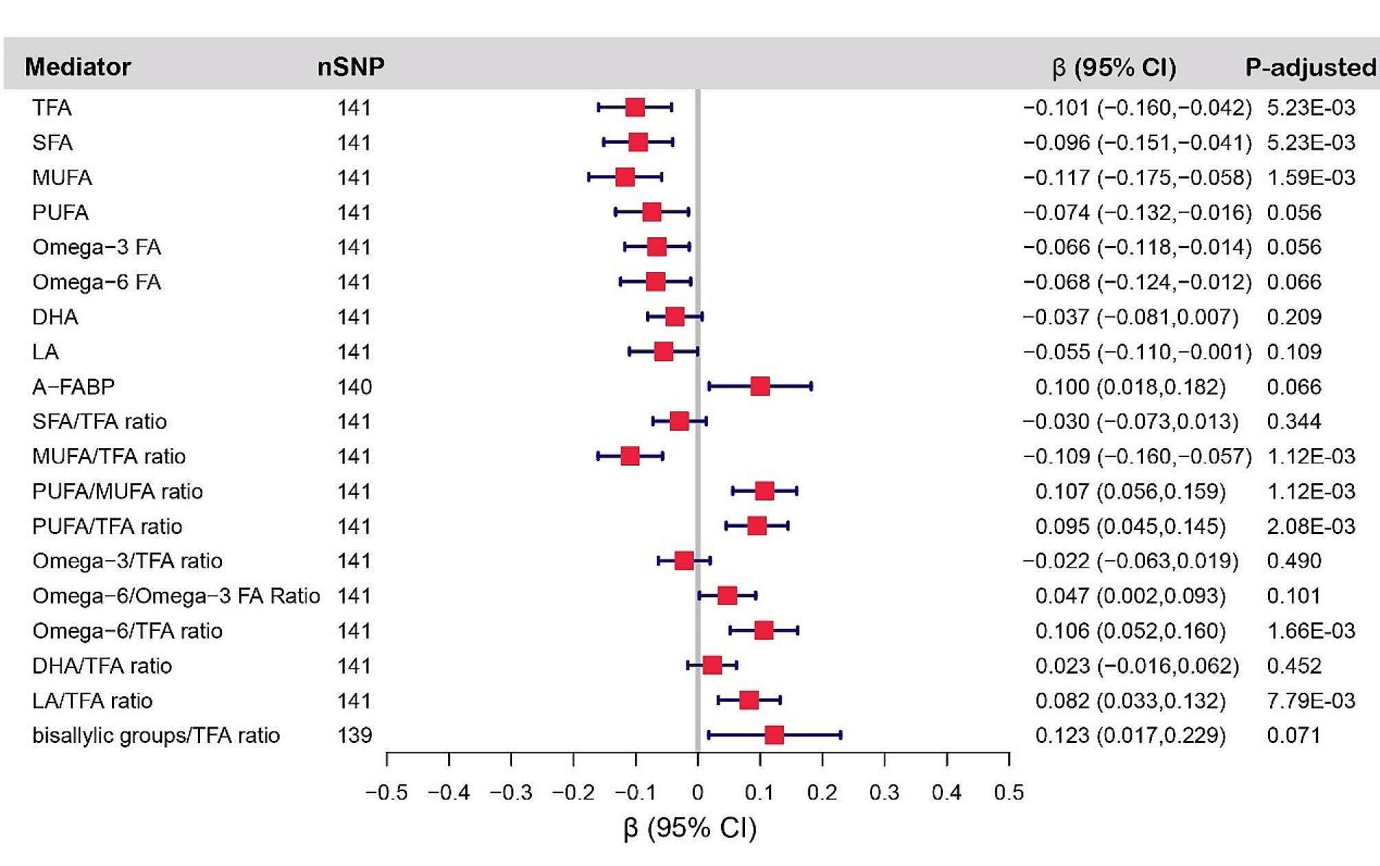
MR estimates derived from the IVW method to assess the causal effect of birth weight on fatty acids traits. A-FABP, fatty acid-binding protein, adipocyte levels; bisallylic groups/TFA ratio, ratio of bisallylic groups to total fatty acids; CI, confidence interval; DHA, docosahexaenoic acid levels; DHA/TFA ratio, ratio of docosahexaenoic acid to total fatty acid levels; LA, linoleic acid levels; LA/TFA ratio, ratio of linoleic acid to total fatty acids; MUFA, monounsaturated fatty acid levels; MUFA/TFA ratio, ratio of monounsaturated fatty acids to total fatty acids; Omega-3 FA, omega-3 fatty acid levels; Omega-3/TFA ratio, ratio of omega-3 fatty acids to total fatty acids; Omega-6 FA, omega-6 fatty acid levels; Omega-6/Omega-3 FA ratio, ratio of omega-6 fatty acids to omega-3 fatty acids; Omega-6/TFA ratio, ratio of omega-6 fatty acids to total fatty acids; PUFA, polyunsaturated fatty acid levels; PUFA/MUFA ratio, ratio of polyunsaturated fatty acids to monounsaturated fatty acids; PUFA/TFA ratio, ratio of polyunsaturated fatty acids to total fatty acids; SFA, saturated fatty acid levels; SFA/TFA ratio, ratio of saturated fatty acids to total fatty acids; TFA, total fatty acid levels

Genetically predicted each 1-SD lower birth weight was associated with higher TFA (β: −0.101, −0.160 ∼ −0.042), SFA (β: −0.096, −0.151 ∼ −0.041) and MUFA (β: −0.117, −0.175 ∼ −0.058). Besides, IVW results also showed that genetically predicted each 1-SD decrease in birth weight was associated with lower PUFA/MUFA ratio (β: 0.107, 0.056 ∼ 0.159), PUFA/TFA ratio (β: 0.095, 0.045 ∼ 0.145), Omega-6/TFA ratio (β: 0.106, 0.052 ∼ 0.160) and LA/TFA ratio (β: 0.082, 0.033 ∼ 0.132), but higher MUFA/TFA ratio (β: −0.109, −0.160 ∼ −0.057) (Fig. 4).

In addition, reverse MR suggested that there was no evidence for a causal effect of fatty acids traits on birth weight. (Table S12) The presence of heterogeneity and pleiotropic effect are shown in Table S14-16.

### Effects of fatty acids traits on T2DM and glycemic quantitative traits

**Fig. 5:**
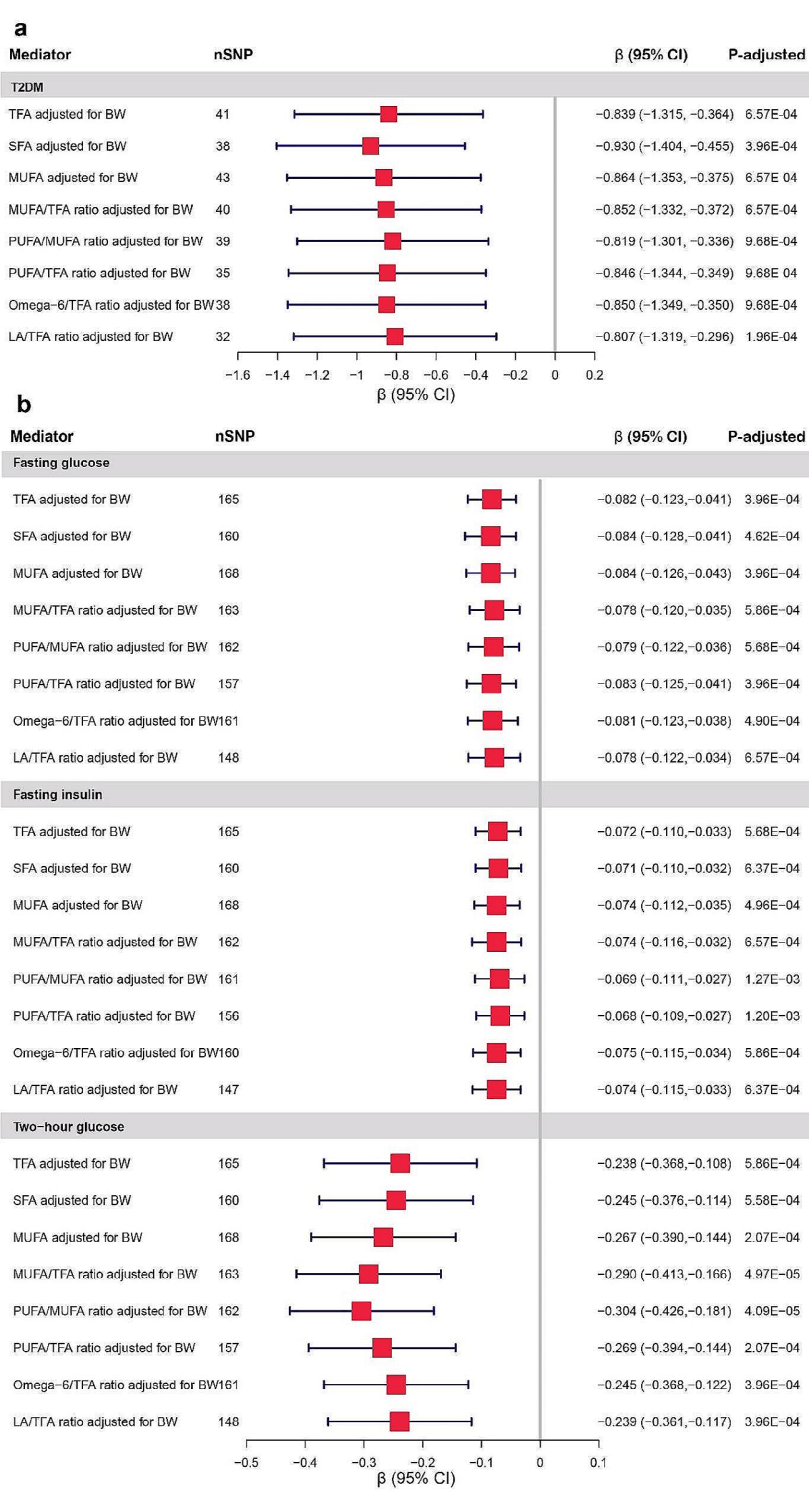
**a** MR estimates derived from the IVW method to assess the causal effect of fatty acids traits on T2DM after adjusting for birth weight. **b** MR estimates derived from the IVW method to assess the causal effect of fatty acids traits on fasting glucose, fasting insulin and two-hour glucose after adjusting for birth weight. BW, birth weight; CI, confidence interval; LA/TFA ratio, ratio of linoleic acid to total fatty acids; MUFA, monounsaturated fatty acid levels; MUFA/TFA ratio, ratio of monounsaturated fatty acids to total fatty acids; Omega-6/TFA ratio, ratio of omega-6 fatty acids to total fatty acids; OR, odds ratio; PUFA/MUFA ratio, ratio of polyunsaturated fatty acids to monounsaturated fatty acids; PUFA/TFA ratio, ratio of polyunsaturated fatty acids to total fatty acids; SFA, saturated fatty acid levels; TFA, total fatty acid levels

In MVMR, TFA (β: −0.839, 95% CI: −1.315 ∼ −0.364), SFA (β: −0.930, −1.404 ∼ −0.455), and MUFA (β: −0.864, −1.353 ∼ −0.375) were negatively associated with T2DM after adjusting for birth weight. Besides, lower MUFA/TFA ratio (β: −0.852, −1.332 ∼ −0.372), PUFA/MUFA ratio (β: −0.819, −1.301 ∼ −0.336), PUFA/TFA ratio (β: −0.846, −1.344 ∼ −0.349), Omega-6/TFA ratio (β: −0.850, −1.349 ∼ −0.350), and LA/TFA ratio (β: −0.807, −1.319 ∼ −0.296) were in relation to an increased risk of T2DM after adjusting for birth weight (Fig. 5a).

Genetically determined each 1-SD higher fatty acids traits with adjustment for birth weight showed consistently causal associations with fasting glucose, fasting insulin and two-hour glucose (Fig. 5b).

### Mediation effects of fatty acids traits on T2DM and glycemic quantitative traits

For the causal effect of birth weight on T2DM, the percentage mediated by PUFA/MUFA ratio, PUFA/TFA ratio, Omega-6/TFA ratio and LA/TFA ratio was 7.9% (95%CI: 1.1%∼14.7%), 7.2% (0.8%∼13.7%), 8.1% (0.9%∼15.2%) and 6.0% (0.2%∼11.7%), respectively. The mediation effects of PUFA/MUFA ratio, PUFA/TFA ratio, Omega-6/TFA ratio and LA/TFA ratio on fasting glucose, fasting insulin, and two-hour glucose were estimated to account for 7.6%∼13.9%. Table 2 shows the mediated effect of birth weight on T2DM, fasting glucose, fasting insulin and two-hour glucose explained by each mediator separately.

**Table 2:** Proportion of the effect of birth weight on T2DM, fasting glucose, fasting insulin and two-hour glucose mediated by cardiometabolic factors

Exposure	β1(SE)	Mediator	β2(SE)	Outcome	β0(SE)	Mediation proportion
Birth weight	0.107 (0.026)	PUFA/MUFA ratio	−0.819 (0.246)	T2DM	−1.113 (0.235)	7.9% (1.1%,14.7%)
	0.095 (0.025)	PUFA/TFA ratio	−0.846 (0.254)			7.2% (0.8%,13.7%)
	0.106 (0.028)	Omega-6/TFA ratio	−0.850 (0.255)			8.1% (0.9%,15.2%)
	0.082 (0.025)	LA/TFA ratio	−0.807 (0.261)			6.0% (0.2%,11.7%)
Birth weight	0.107 (0.026)	PUFA/MUFA ratio	−0.079 (0.022)	Fasting glucose	−0.061 (0.015)	13.8% (1.8%,25.9%)
	0.095 (0.025)	PUFA/TFA ratio	−0.083 (0.022)			12.8% (1.6%,24.1%)
	0.106 (0.028)	Omega-6/TFA ratio	−0.081 (0.022)			13.9% (1.7%,26.2%)
	0.082 (0.025)	LA/TFA ratio	−0.078 (0.022)			10.5% (0.5%,20.5%)
Birth weight	0.107 (0.026)	PUFA/MUFA ratio	−0.069 (0.021)	Fasting insulin	−0.080 (0.014)	9.3% (1.4%,17.2%)
	0.095 (0.025)	PUFA/TFA ratio	−0.068 (0.021)			8.1% (1.0%,15.2%)
	0.106 (0.028)	Omega-6/TFA ratio	−0.074 (0.021)			9.8% (1.7%,17.9%)
	0.082 (0.025)	LA/TFA ratio	−0.074 (0.021)			7.6% (0.8%,14.4%)
Birth weight	0.107 (0.026)	PUFA/MUFA ratio	−0.304 (0.063)	Two-hour glucose	−0.250 (0.056)	13.0% (3.0%,23.0%)
	0.095 (0.025)	PUFA/TFA ratio	−0.269 (0.064)			10.2% (1.8%,18.7%)
	0.106 (0.028)	Omega-6/TFA ratio	−0.245 (0.063)			10.4% (1.6%,19.1%)
	0.082 (0.025)	LA/TFA ratio	−0.239 (0.062)			7.9% (0.7%,15.0%)

*Abbreviations* LA/TFA ratio, ratio of linoleic acid to total fatty acids; Omega-6/TFA ratio, ratio of omega-6 fatty acids to total fatty acids; PUFA/MUFA ratio, ratio of polyunsaturated fatty acids to monounsaturated fatty acids; PUFA/TFA ratio, ratio of polyunsaturated fatty acids to total fatty acids

## Discussion

In this study, we conducted a series of two-sample MR and two-step MR analyses to assess the independent impact of birth weight on T2DM and glycemic quantitative traits, identify potential metabolic mediators and quantify the mediation effects. Our findings provided robust evidence supporting the negative causal effect of birth weight on T2DM. Furthermore, the two-step MR analysis indicated that 4 of 17 candidate mediators were identified to partially mediate the causal effect of low birth weight on T2DM, including PUFA/MUFA ratio, PUFA/TFA ratio, Omega-6/TFA ratio, and LA/TFA ratio. These mediators also exhibited corresponding mediating effects on fasting glucose, fasting insulin, and two-hour glucose. Therefore, interventions targeted at these factors could potentially reduce the risk of T2DM among individuals with low birth weight.

T2DM is a significant societal burden [20, 21]. Our study indicated that a lower birth weight was associated with a higher risk of T2DM, fasting insulin, fasting glucose, and two-hour glucose. A recent systematic review involving 152,084 individuals supported a negative correlation between birth weight and T2DM [22]. However, Tamarra M. James-Todd’s study suggested that low birth weight did not significantly increase the risk of T2DM [23]. The increased incidence of impaired glucose tolerance and T2DM in adulthood with low birth weight can be explained from two perspectives. Firstly, from the genetic aspect, the fetal insulin hypothesis proposes that low birth weight and T2DM in adulthood are two phenotypes of the same gene. The loci that primarily affect pancreatic β-cell function, such as ADCY5 and CDKAL1, show the strongest associations between T2DM risk alleles and lower birth weight. Utilizing genome-wide data, common variants can explain 36% of the negative correlation between birth weight and T2DM. Secondly, we explained this result from the maternal impact. During pregnancy, the mother transmits relevant environmental information (such as nutritional status) to the fetus through the placenta [24]. The Developmental Origins of Health and Disease (DOHaD) suggests that various adverse early-life conditions, including nutrition imbalance, maternal conditions or diseases, maternal chemical exposure, and medication use can lead to vulnerability to later metabolic disorders [25]. Lumey LH’s investigation found that individuals born in famine areas in Ukraine exhibited decreased glucose tolerance about 50 years later [26]. Additionally, low birth weight reflects poor intrauterine nutritional status, which could lead to alterations in pancreatic β-cell structure and function [27], skeletal muscle abnormalities [28], and hypothalamus-pituitary-adrenal axis dysfunction [29], further influencing the occurrence of T2DM.

Infants with low birth weight often experience catch-up growth under nutrient sufficient conditions [30]. Due to parental concerns, they tend to consume more fatty acids, including TFA, SFA, MUFA, PUFA, Omega-3 FA, and Omega-6 FA. However, we observed a decrease in the ratios of PUFA/MUFA, PUFA/TFA, Omega-6/TFA, and LA/TFA, which could be associated with factors such as the types of fatty acids consumed and metabolic processes. When exposed to a metabolic challenge of high-fat overfeeding, low birth weight subjects could lead to peripheral insulin resistance and T2DM, possibly associated with decreased expression of OXPHOS genes and mitochondrial dysfunction [31, 32]. Furthermore, a mouse experiment suggested that catch-up growth in fetuses leads to severe impairment of insulin sensitivity [33].

TFA includes SFA and UFA. High SFA content in diets could adversely affect insulin sensitivity, potentially leading to the development of T2DM [34]. The potential mechanism involves the loss of β-cell fat storage-inducing transmembrane protein 2 (FIT2) and lipid droplet (LD), leading to β cell dysfunction and subsequently causing T2DM [35]. The role of SFA in T2DM remains controversial, with some studies indicating no association [36–38]. The lack of correlation between SFA and T2DM is due to the varying effects of SFA from different food sources. Consuming red meat, which is a primary source of SFA in the European and American diet, is associated with a higher risk of T2DM [39]. In contrast, a negative correlation is observed between the consumption of SFA and T2DM when dairy products, low-fat dairy, and cheese are the primary SFA sources [40].

UFA encompasses MUFA and PUFA. The substitution of carbohydrates with MUFA correlates with an increased T2DM (HR 1.10, [95% CI 1.01, 1.19]) [41]. Oleic acid, the most abundant MUFA, is implicated in lipotoxicity of pancreatic β-cells, thereby promoting the progression of T2DM. Islets exposed to oleic acid elevate the expression of PPI, PDX-1, and GLUT2, potentially contributing to increased basal insulin secretion [42]. Additionally, excessive oleic acid intake induces DNA methylation, which most likely accounts for the decrease of glucose-stimulated insulin secretion (GSIS) [43].

Our study indicated a negative correlation between PUFA/SFA and PUFA/MUFA ratios with T2DM. A study exploring complete data on plasma fatty acids from 95,854 participants in the UK Biobank between 2006 and 2010 revealed a positive association of SFA (OR: 1.03) and MUFA (OR: 1.03) with T2DM, while PUFA (OR: 0.62) showed a negative association with T2DM [44]. Substituting dietary SFA with PUFA improved insulin sensitivity within just 5 weeks, thereby decreasing the risk of developing T2DM [45]. Fumiaki et al. [46] suggested that replacing SFA or MUFA with PUFA leads to significant reductions in blood glucose levels. The findings of the previous studies mentioned above are consistent with our results. In animal studies, PUFA primarily enhanced insulin sensitivity in skeletal muscle and adipose tissue, thereby improving glucose homeostasis [47, 48].

In our study, Omega-6/SFA showed a negative correlation with T2DM. Omega-6 fatty acids are a class of PUFA, and their impact on T2DM remains controversial. Specifically, LA and eicosadienoic acid (EDA) exhibit a negative correlation with T2DM, while arachidonic acid (AA) shows no significant correlation. Additionally, gamma-linolenic acid (GLA), dihomo-GLA, docosatetraenoic acid (DTA), and n6-docosapentaenoic acid (n6-DPA) are significantly positively correlated with T2DM [49].

LA, as an essential fatty acid, is the most abundant Omega-6 FA [50]. Studies suggested a negative correlation between LA intake and T2DM [51, 52], although some studies found no significant association [53]. In a multivariable-adjusted pooled analysis, a higher LA/TFA ratio was associated with an overall lower risk of T2DM, which was in agreement with our study [54].

The primary component of vegetable oils is Omega-6 FA, in particular LA. And nuts are nutrition-dense foods, abundant in PUFA and some bioactive compounds, providing benefits for glycemic regulation [55]. Therefore, increasing the proportion of vegetable oils and nuts consumed may be beneficial in preventing T2DM, particularly in individuals with low birth weight.

Our study possesses several strengths. Firstly, to our knowledge, it is the first MR analysis to assess the association between low birth weight with T2DM risk and glycemic traits, exploring fatty acids traits as intermediaries. Secondly, we investigated not only the content of fatty acids but also the ratio of a series of fatty acids in mediating the impact of low birth weight on T2DM. Thirdly, leveraging large-sample GWAS data enables a more reliable assessment of the relationship between risk factors and disease outcomes compared to observational studies at the individual level. Finally, we employed a variety of MR methods, including weighted median regression, MR-Egger method, simple mode, weighted mode, and robust adjusted profile score, and the consistently estimated associations support the robustness of our study findings.

However, our study has some limitations. Firstly, we assessed the linear effect association between birth weight and T2DM using two-sample MR analysis, lacking exploration into non-linear associations [5, 56–58]. Secondly, we employed the MVMR method to estimate the individual mediating effects of each fatty acid factor. Nevertheless, interactions among these mediators could exist, complicating the precision of individual effects as one mediator could influence others. Thirdly, a random effect model was adopted, taking into consideration the high heterogeneity. Fourthly, considering the limitations in the GWAS summary data, the MR analyses with age, sex or education stratification are unavailable. Fifthly, it is important to note that MR findings only reflect lifetime exposure levels and cannot easily provide information on acute changes in exposure levels. Lastly, it should be noted that the participants in our study were of European descent, so caution should be exercised when generalizing our results to populations of Asian or African descent.

In conclusion, our results supported a potential causal role of low birth weight in T2DM. Additionally, individual fatty acid mediating effects were modest, while their ratios (PUFA/MUFA ratio, PUFA/TFA ratio, Omega-6/TFA ratio, and LA/TFA ratio) demonstrated substantial mediating effects. Therefore, interventions targeting these factors could significantly alleviate the burden of T2DM associated with low birth weight.

## Conclusions

In conclusion, this two-step MR study presented genetic evidence of a causal relationship between low birth weight and T2DM, with PUFA/MUFA ratio, PUFA/TFA ratio, Omega-6/TFA ratio, and LA/TFA ratio serving as mediators. This finding provided novel insights into the underlying mechanisms of the occurrence and development of T2DM and suggested potential avenues for developing preventive and therapeutic strategies.

### Electronic supplementary material

Below is the link to the electronic supplementary material.

Supplementary Material 1

Supplementary Material 2

Supplementary Material 3

Supplementary Material 4

Supplementary Material 5

Supplementary Material 6

## Data Availability

All data used in the current study were obtained from public genome-wide association study summary statistics which were publicly released by genetic consortia. All data is obtained from OpenGWAS (https://gwas.mrcieu.ac.uk/).

## Abbreviations

A-FABP: fatty acid-binding protein adipocyte levels
bisallylic groups/TFA ratio: ratio of bisallylic groups to total fatty acids
CI: confidence interval
DHA: docosahexaenoic acid levels
DHA/TFA ratio: ratio of docosahexaenoic acid to total fatty acid levels
GWAS: genome-wide association study
LA: linoleic acid levels
LA/TFA ratio: ratio of linoleic acid to total fatty acids
MUFA: monounsaturated fatty acid levels
MUFA/TFA ratio: ratio of monounsaturated fatty acids to total fatty acids
Omega-3 FA: omega-3 fatty acid levels
Omega-3/TFA ratio: ratio of omega-3 fatty acids to total fatty acids
Omega-6 FA: omega-6 fatty acid levels
Omega-6/Omega-3 FA ratio: ratio of omega-6 fatty acids to omega-3 fatty acids
Omega-6/TFA ratio: ratio of omega-6 fatty acids to total fatty acids
OR: odds ratio
PUFA: polyunsaturated fatty acid levels
PUFA/MUFA ratio: ratio of polyunsaturated fatty acids to monounsaturatedfatty acids
PUFA/TFA ratio: ratio of polyunsaturated fatty acids to total fatty acids
SD: standard deviation
SFA: saturated fatty acid levels
SFA/TFA ratio: ratio of saturated fatty acids to total fatty acids
TFA: total fatty acid levels
