# Supplementary figures and images for: Association of birth weight with type 2 diabetes mellitus and the mediating role of fatty acids traits: a two-step mendelian randomization study
Source: Lipids Health Dis. 2024 Apr 2;23:97. doi: 10.1186/s12944-024-02087-z (PMC10986016; doi:10.1186/s12944-024-02087-z)

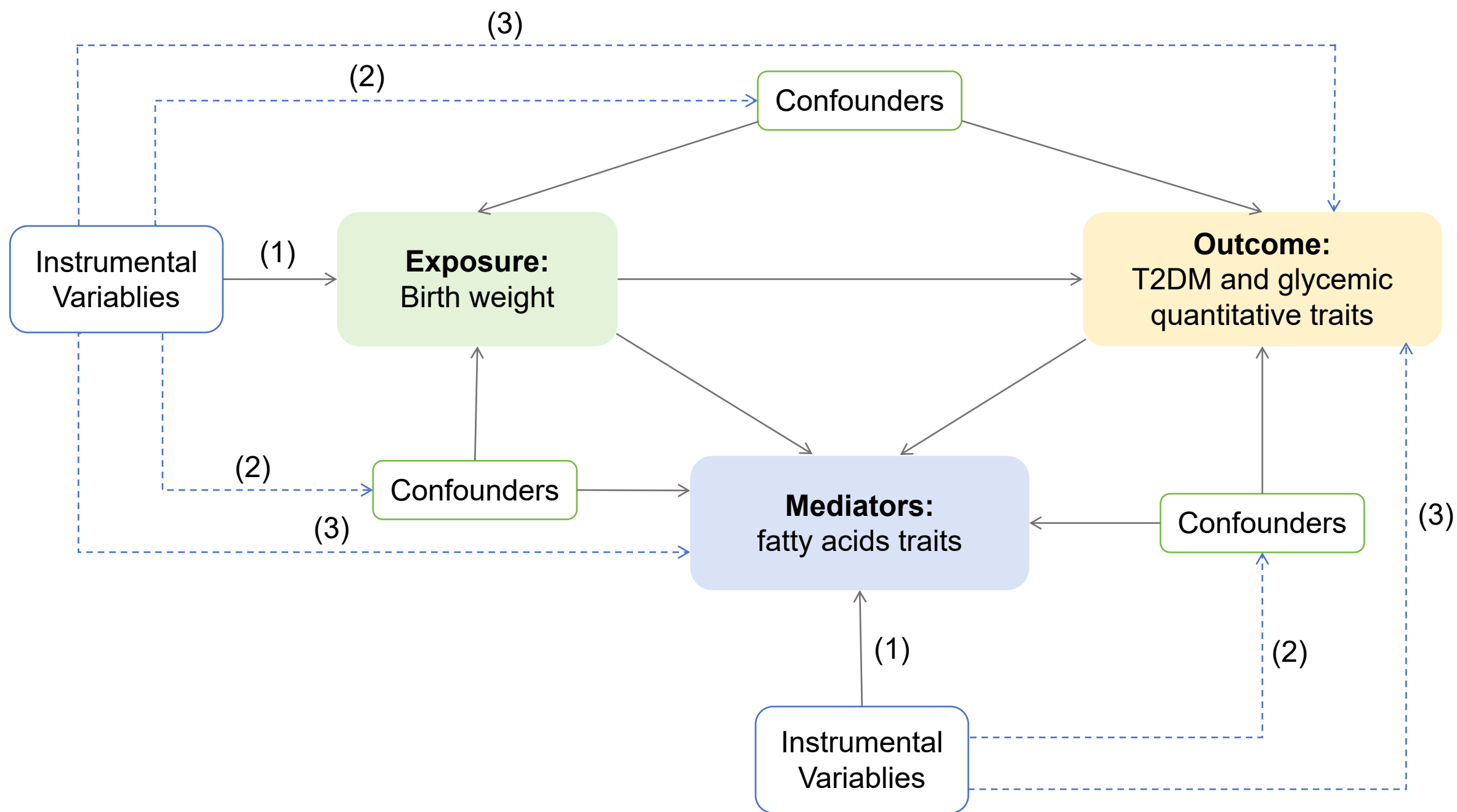

Supplement: Supplementary file 2 — Supplementary Material 2 [file 12944_2024_2087_MOESM2_ESM.pdf]

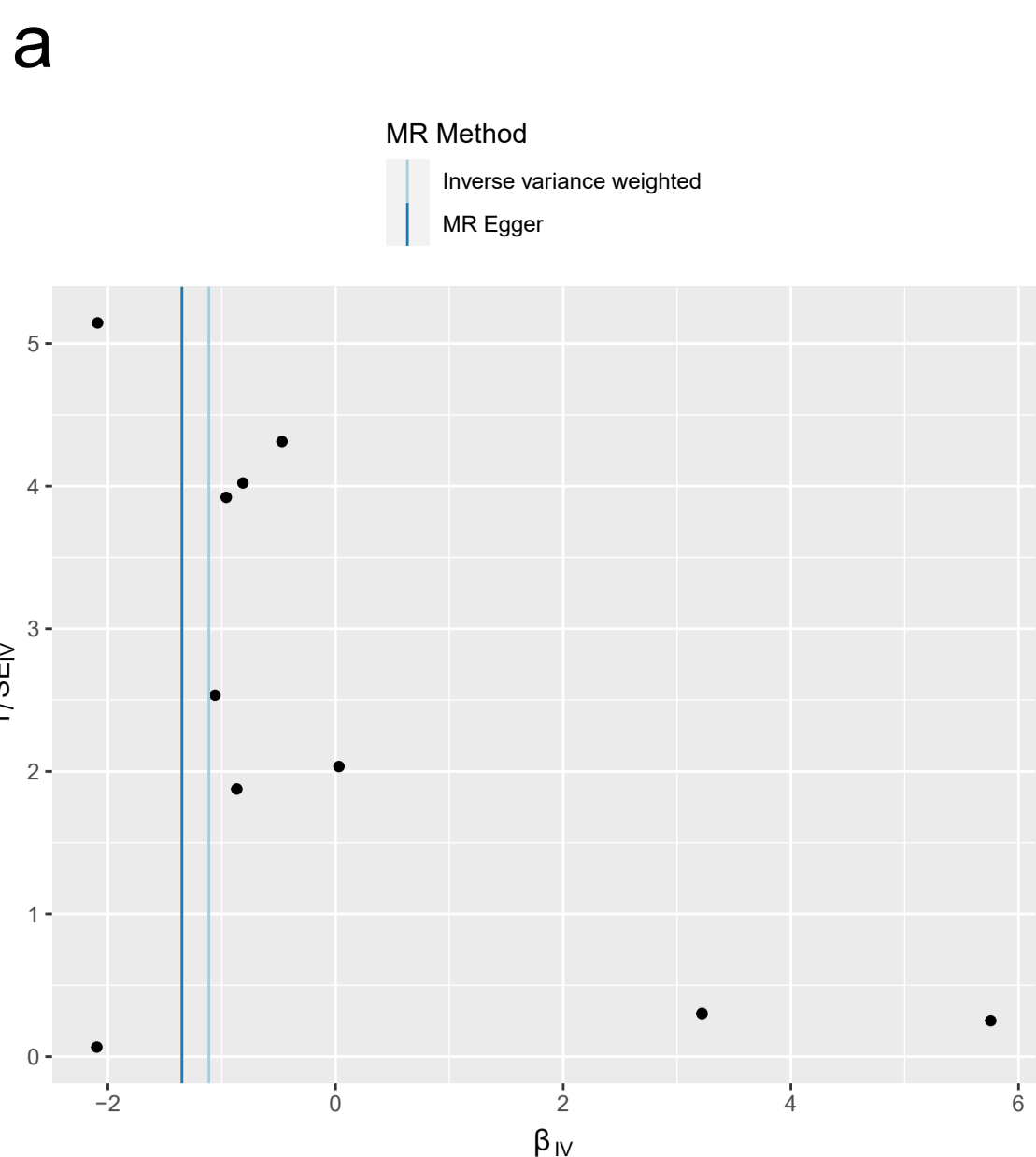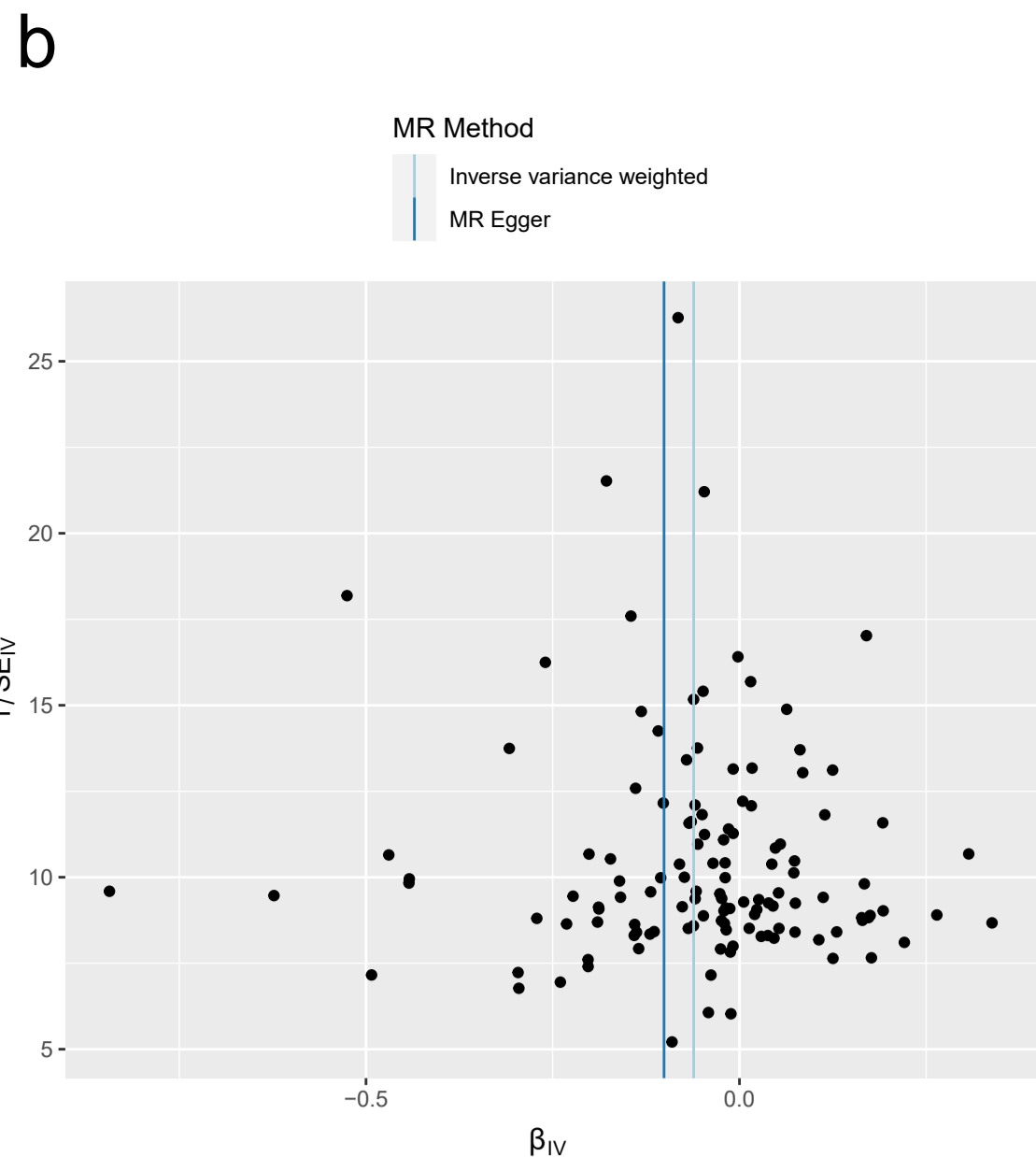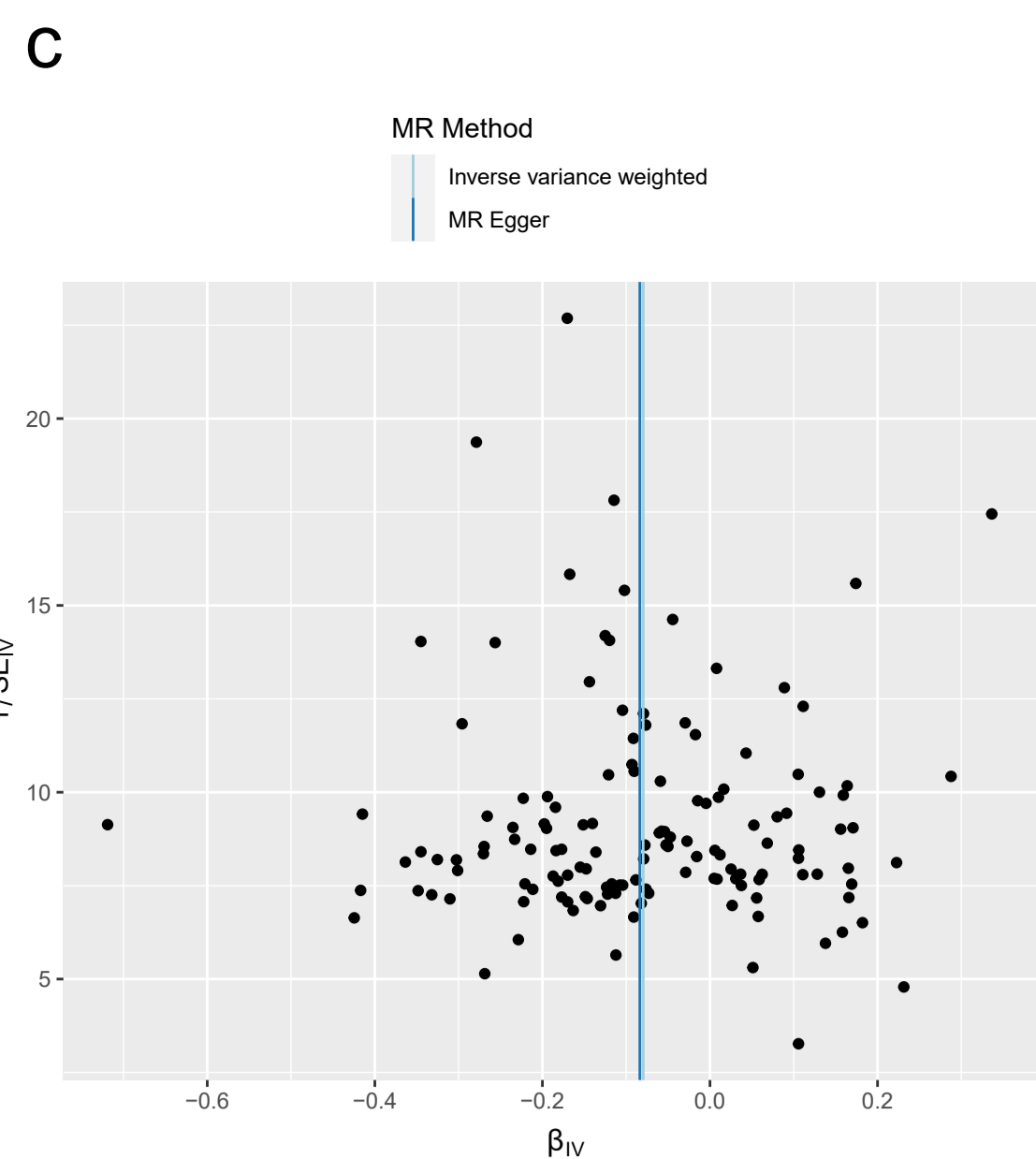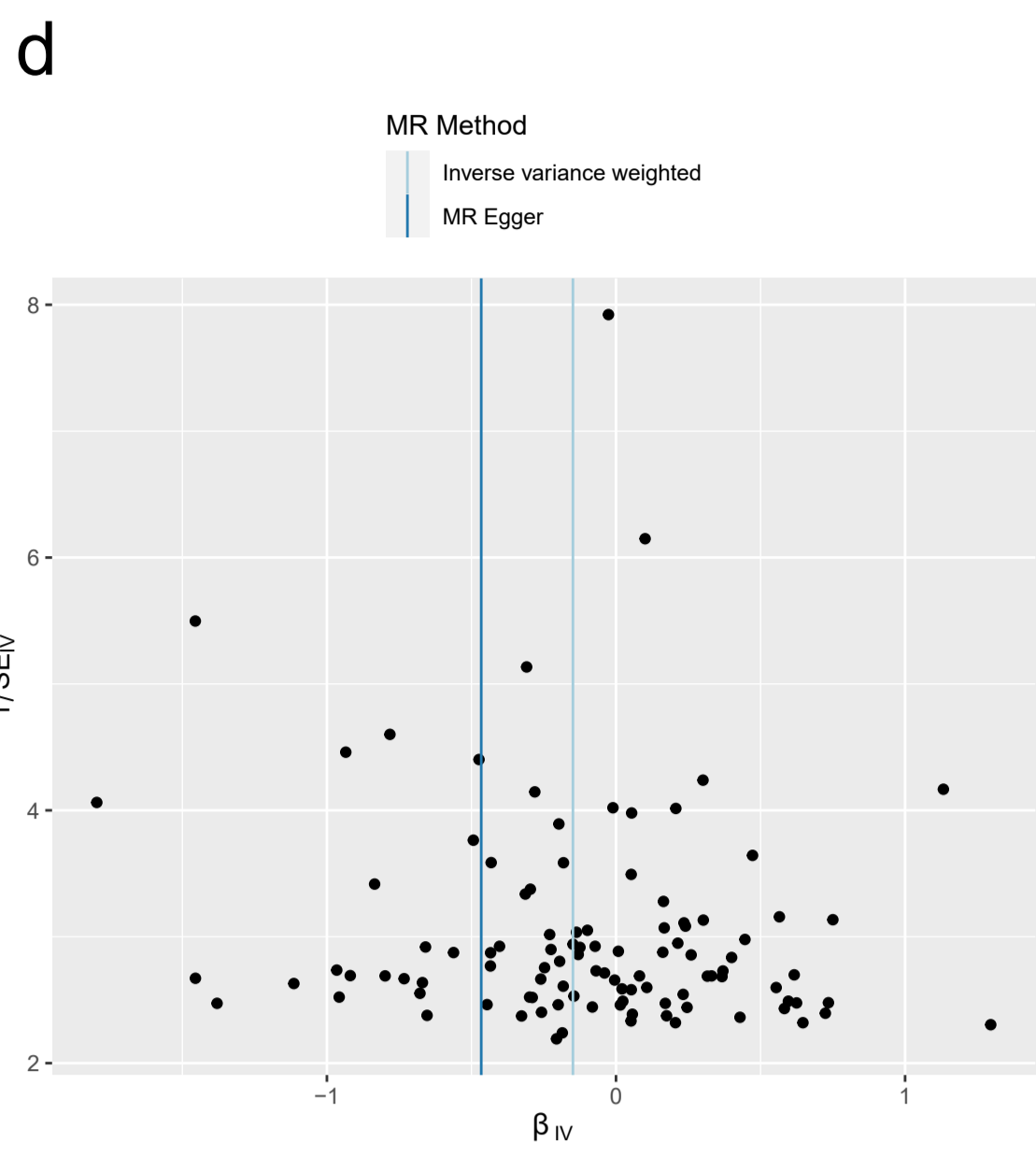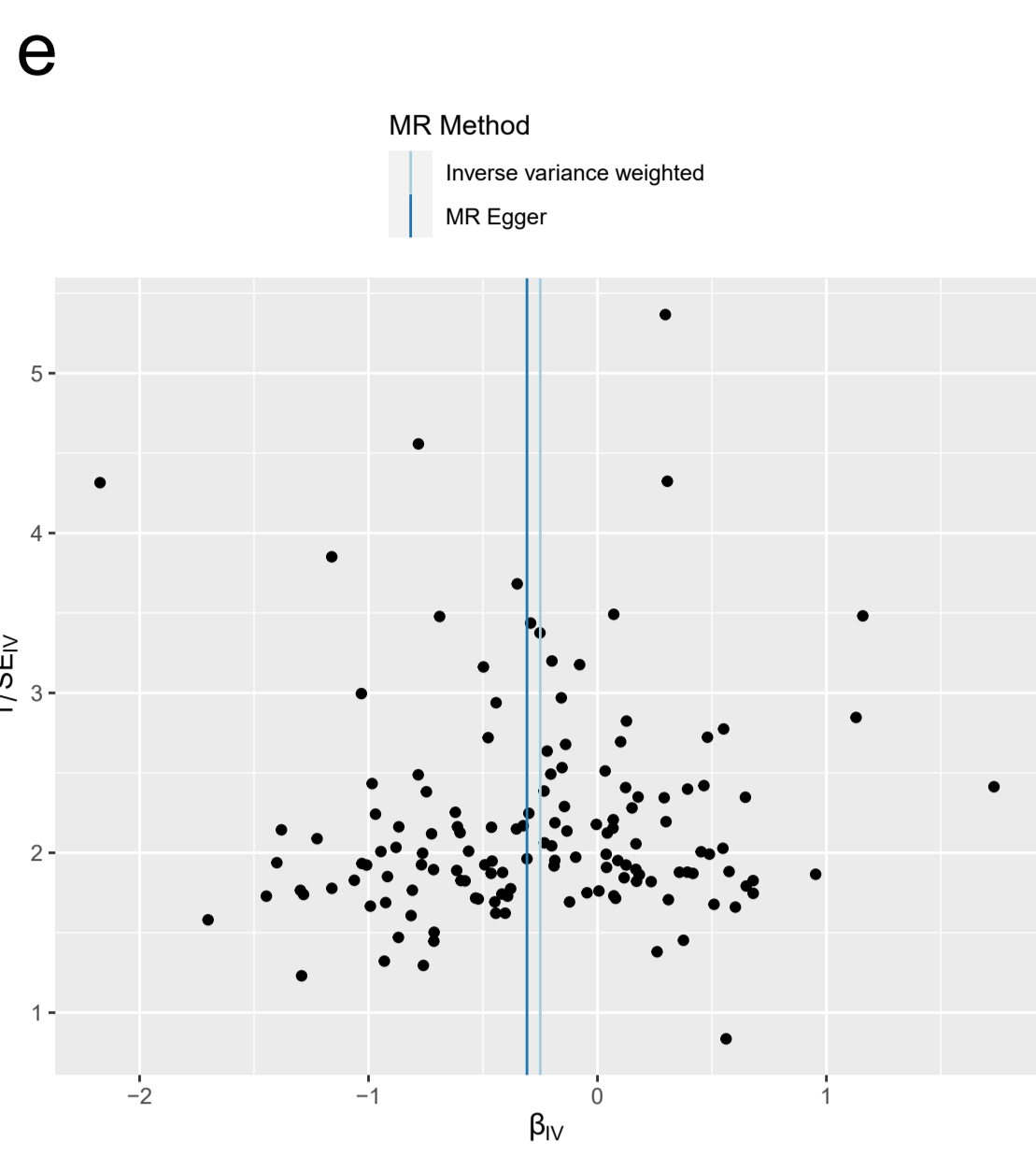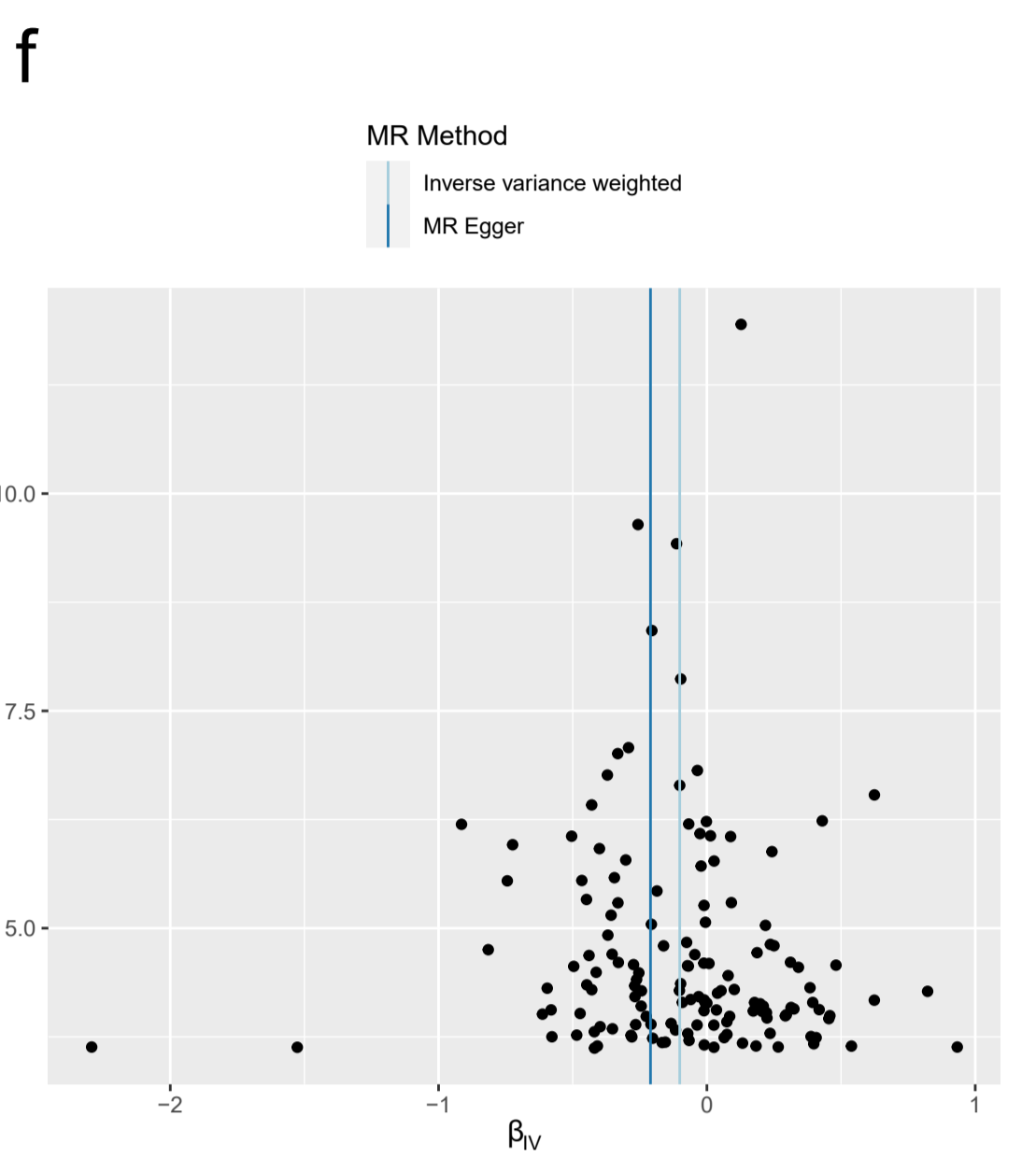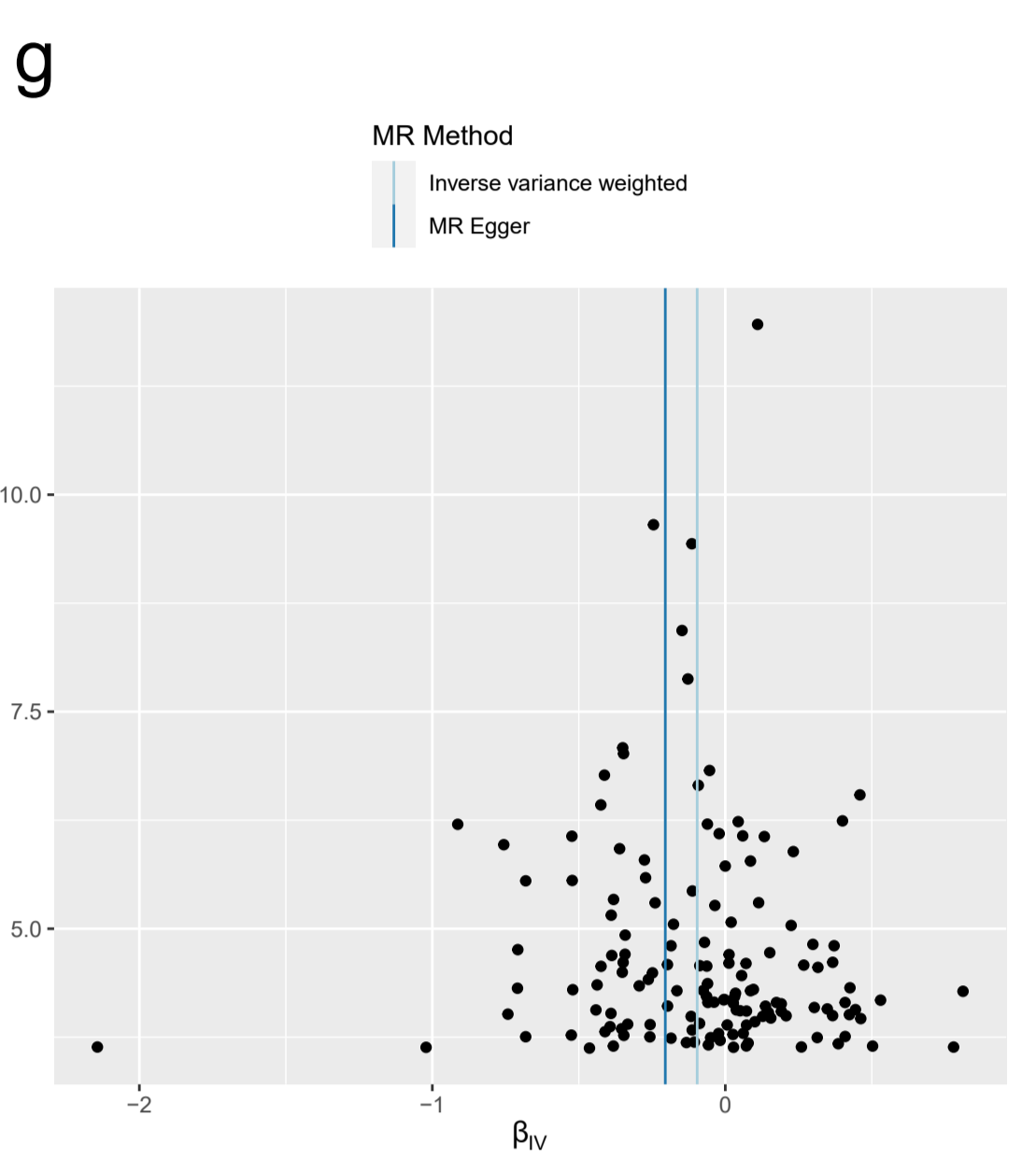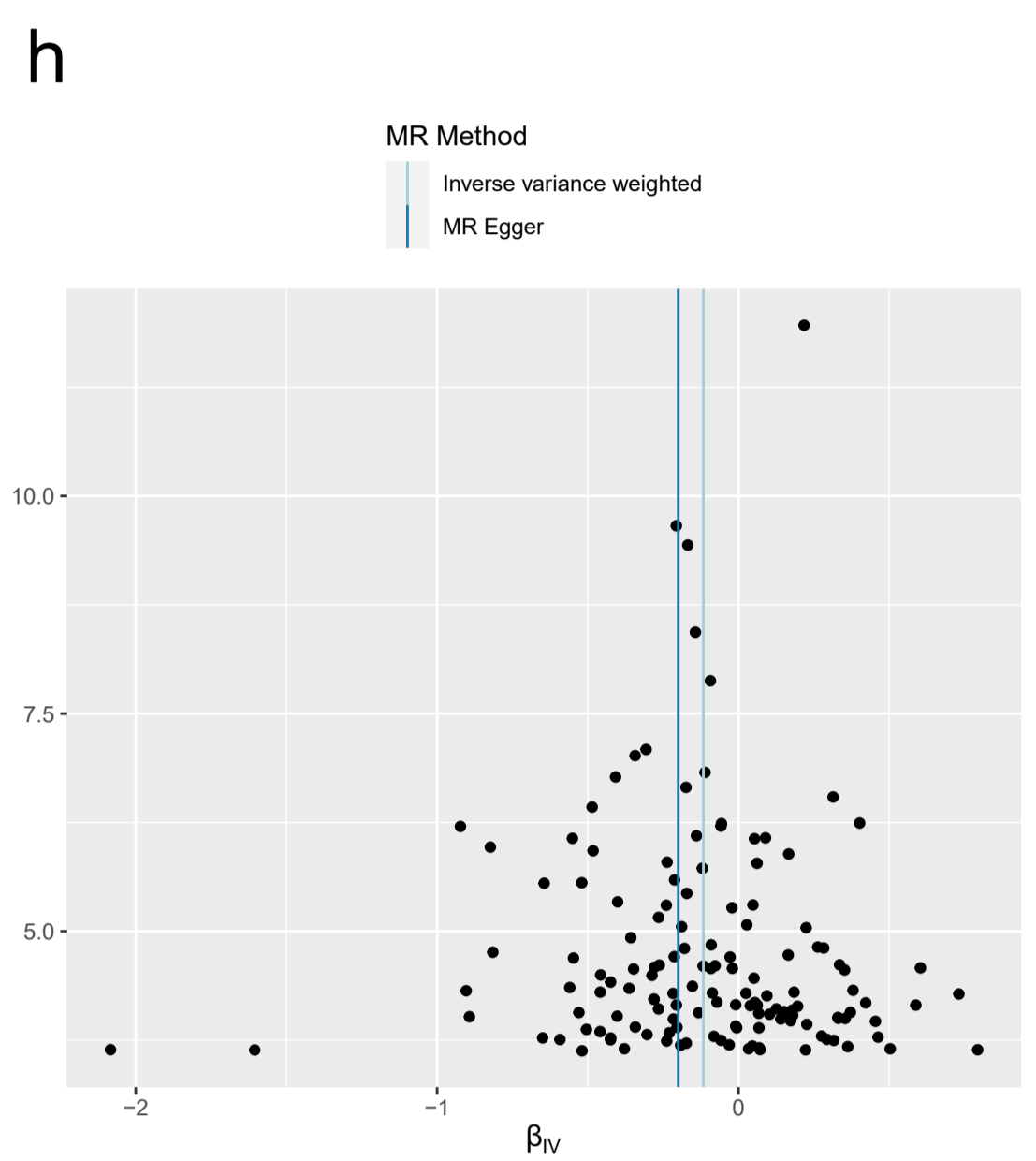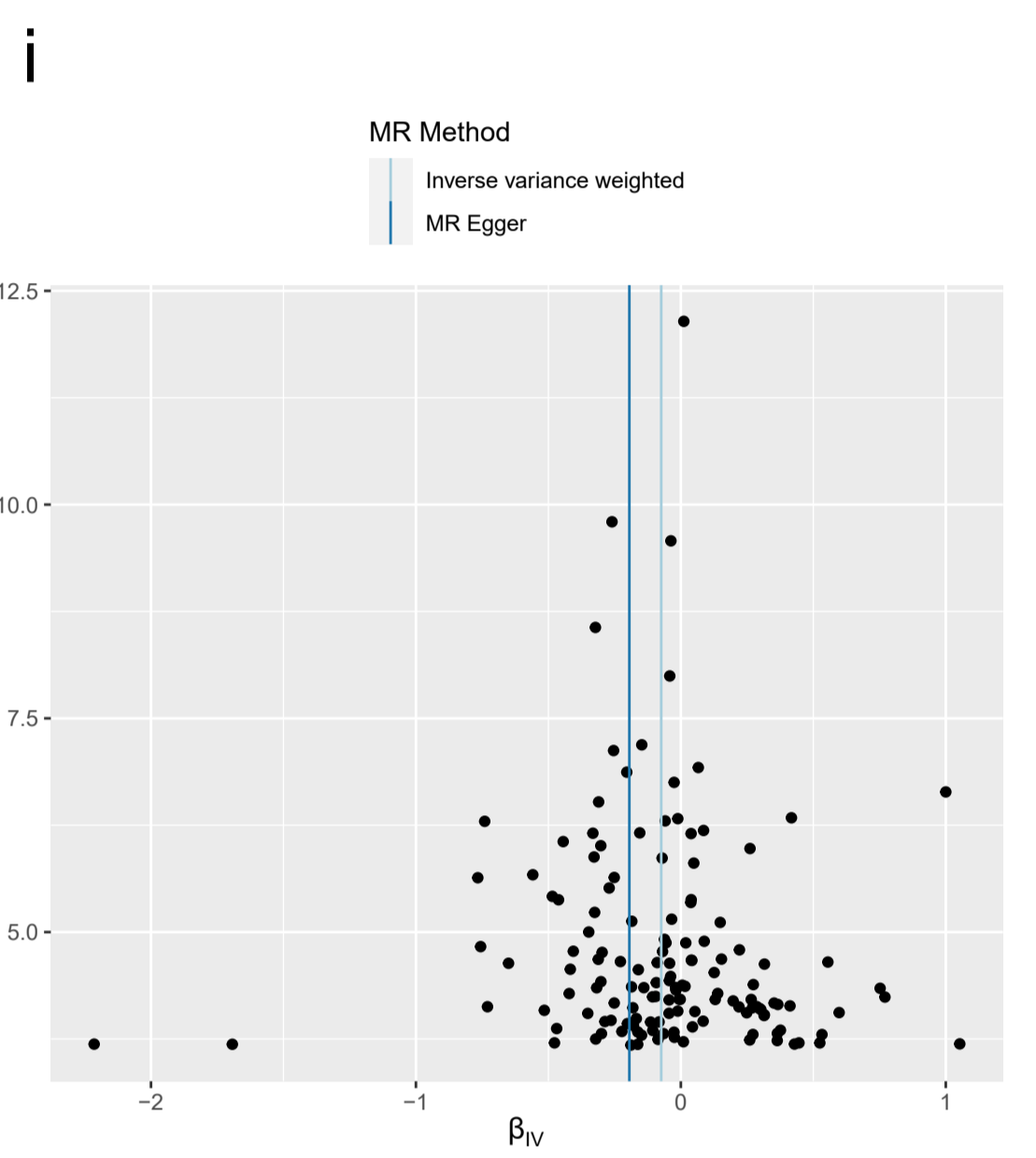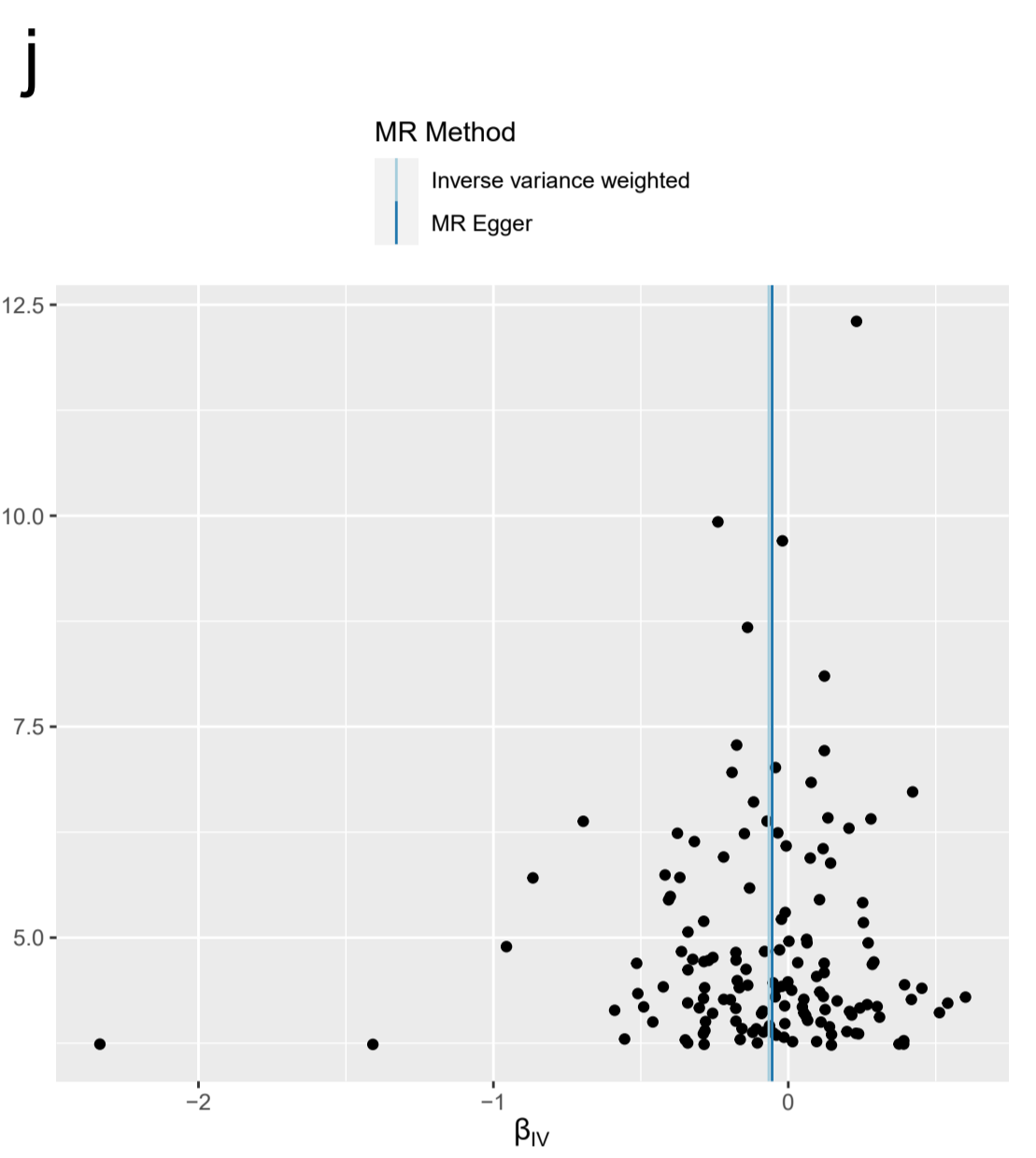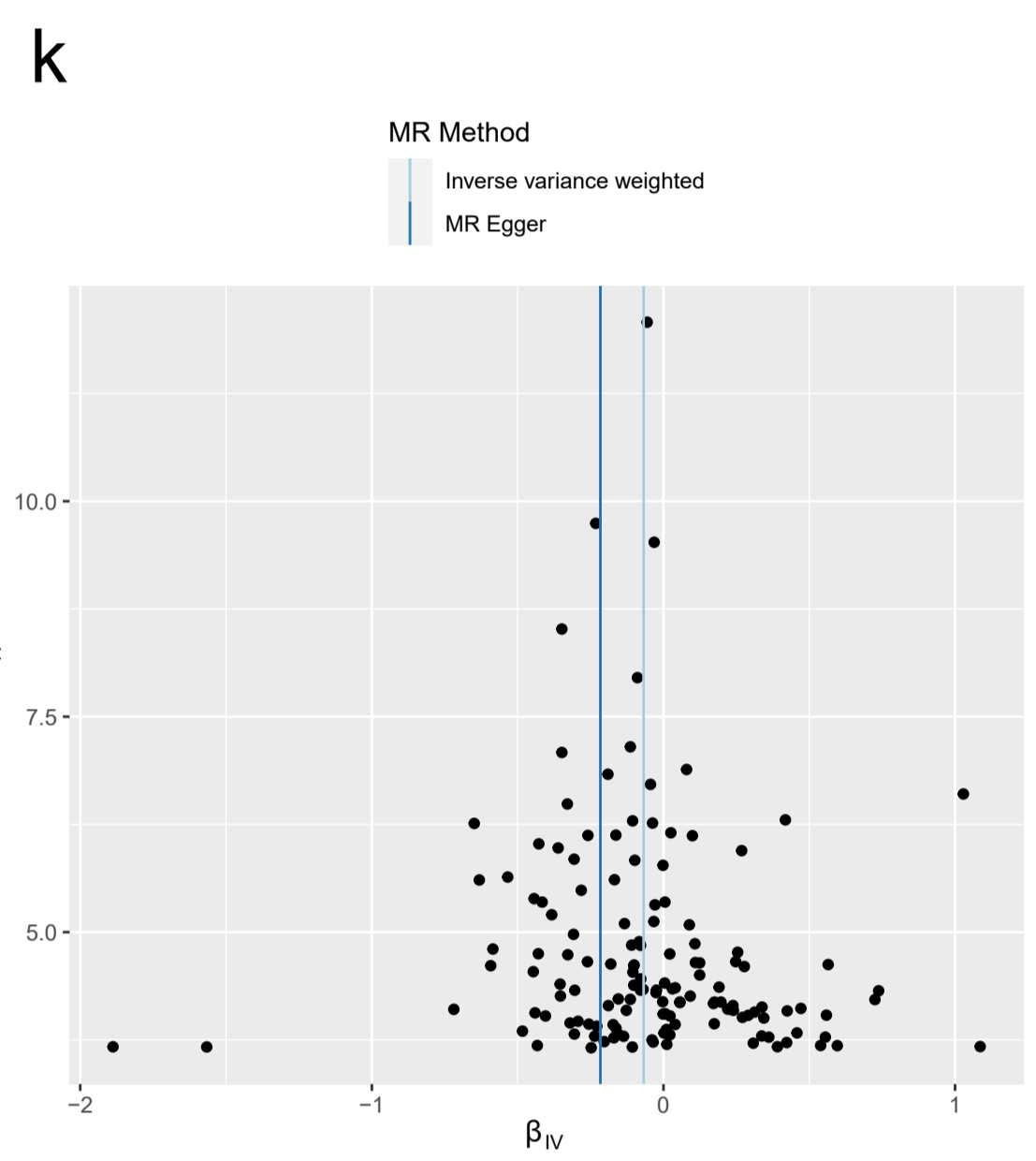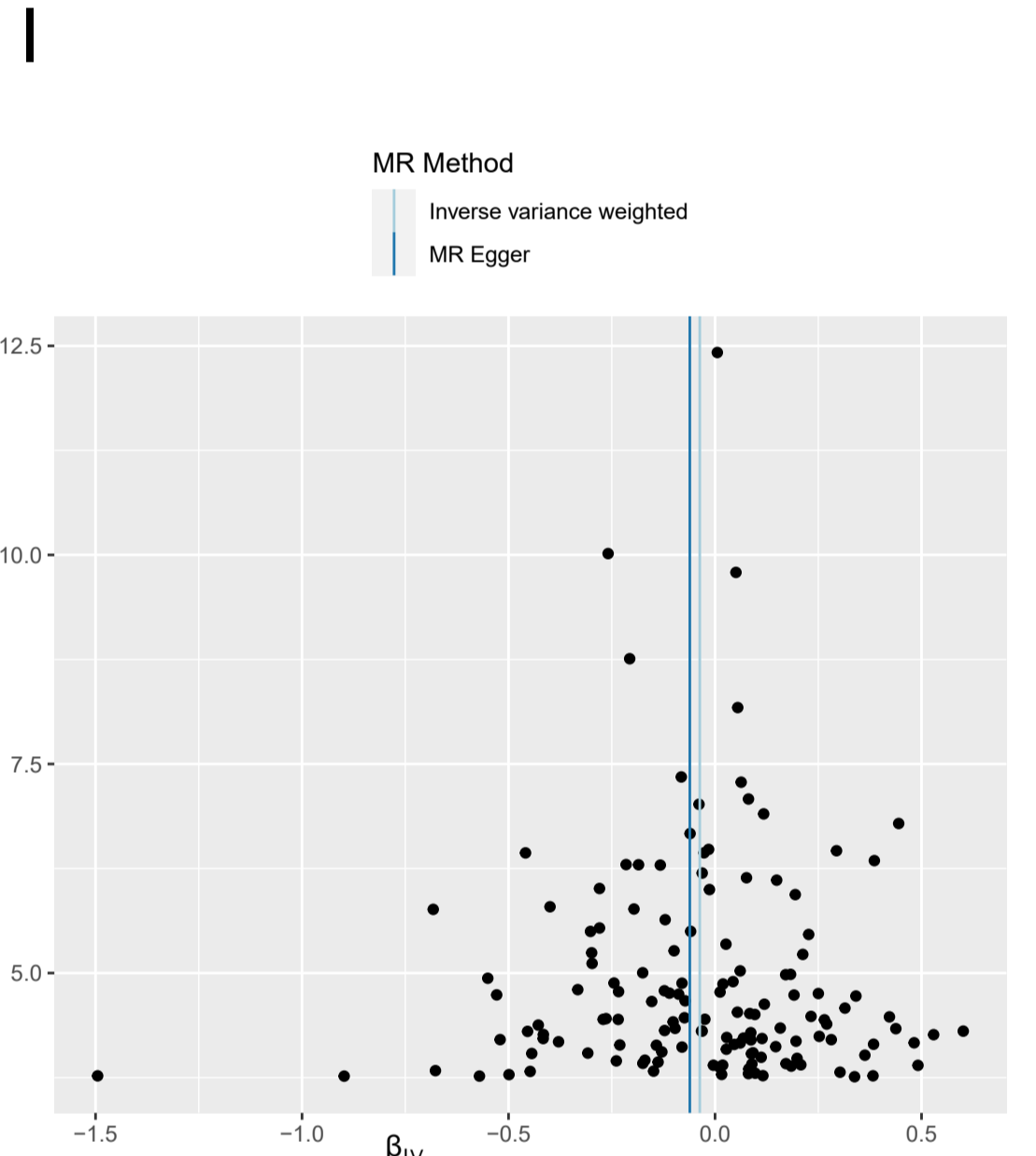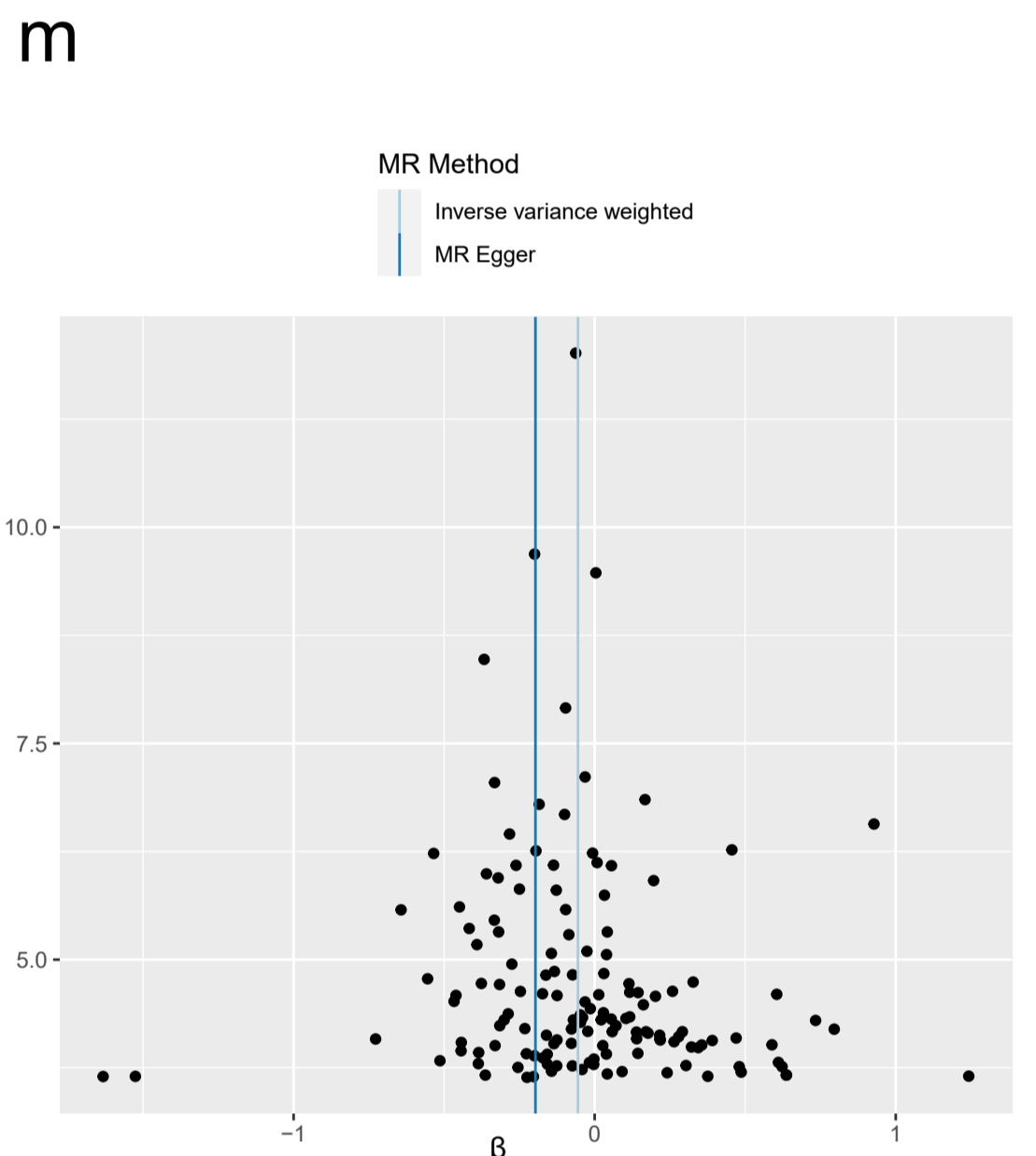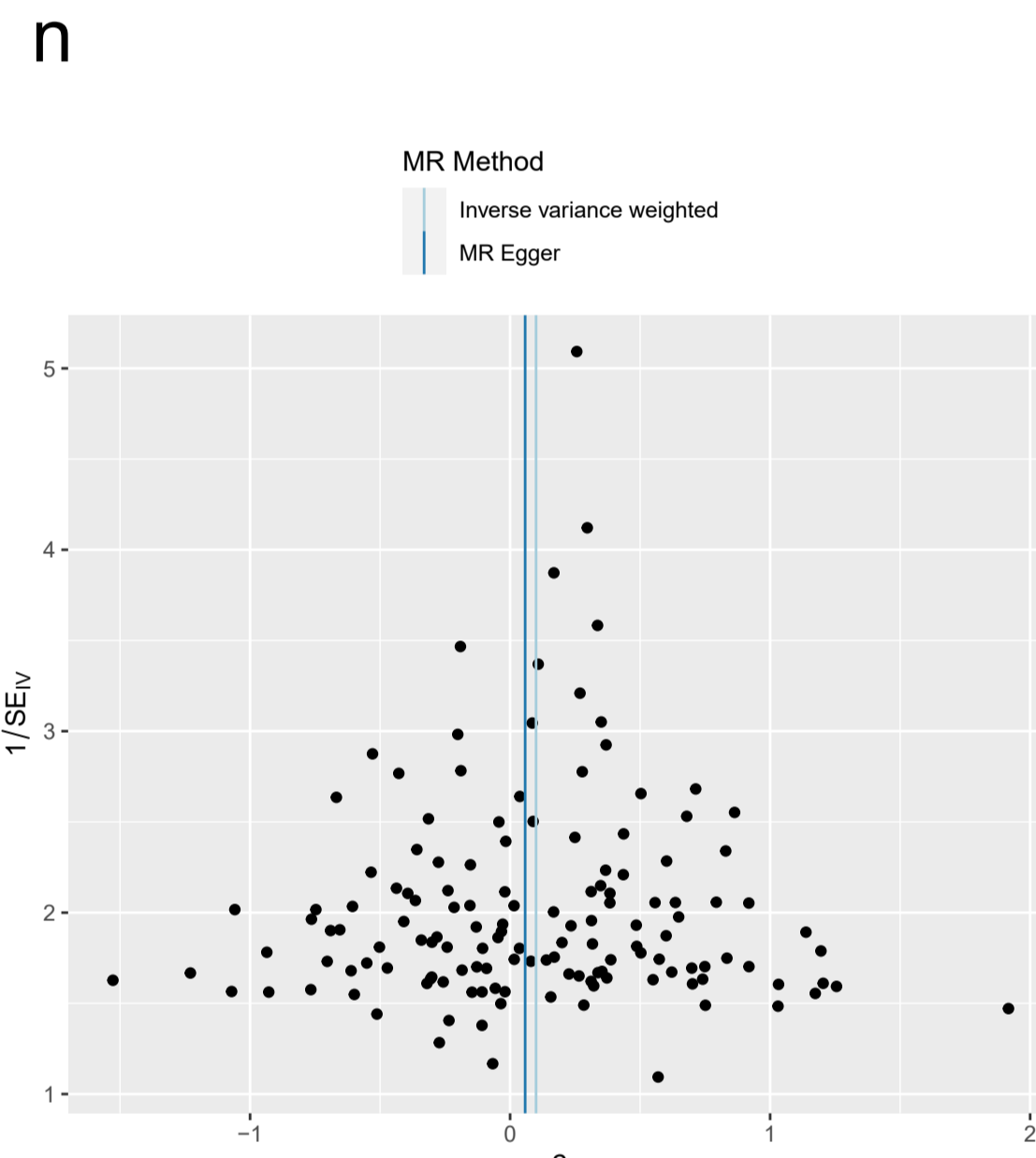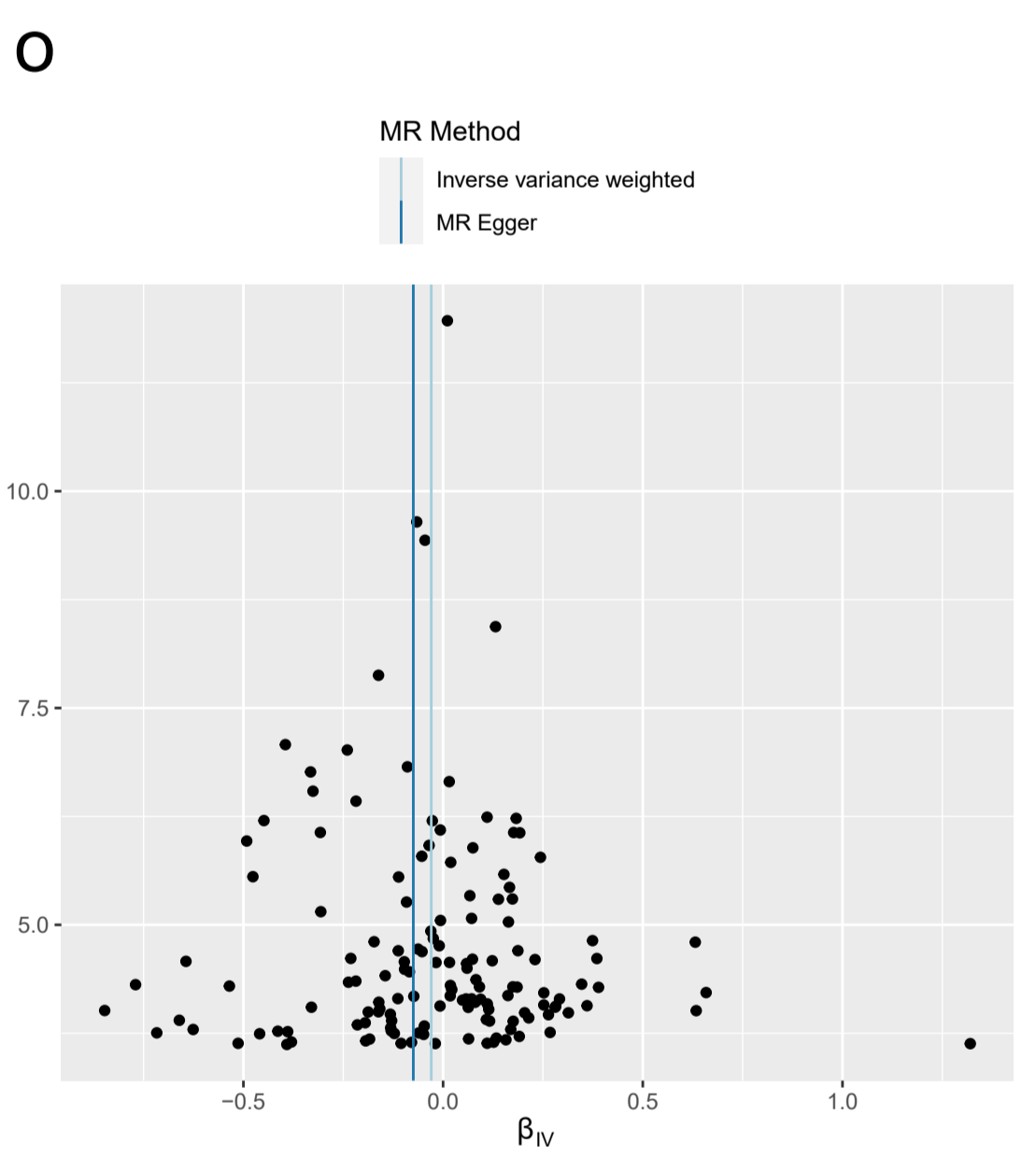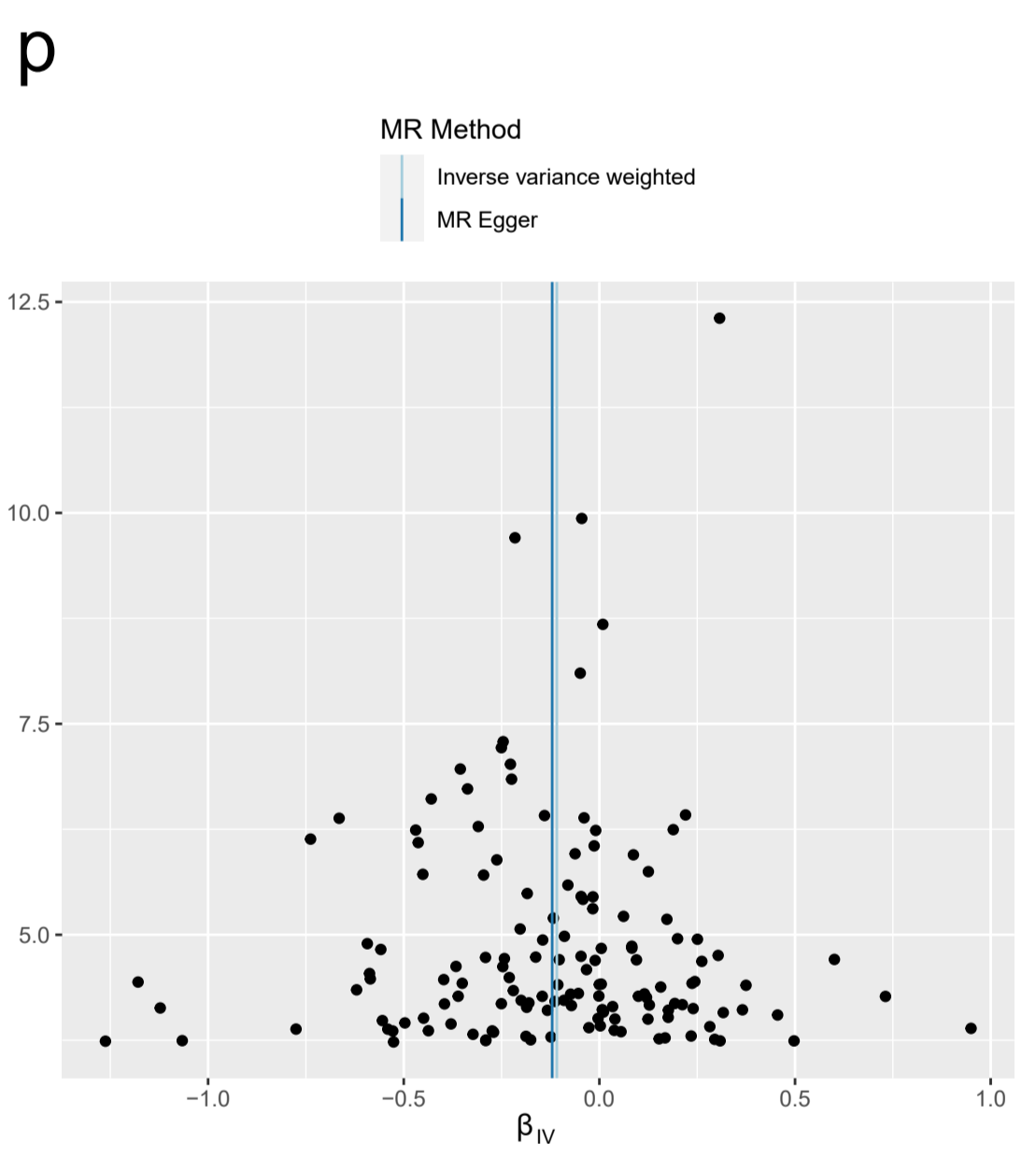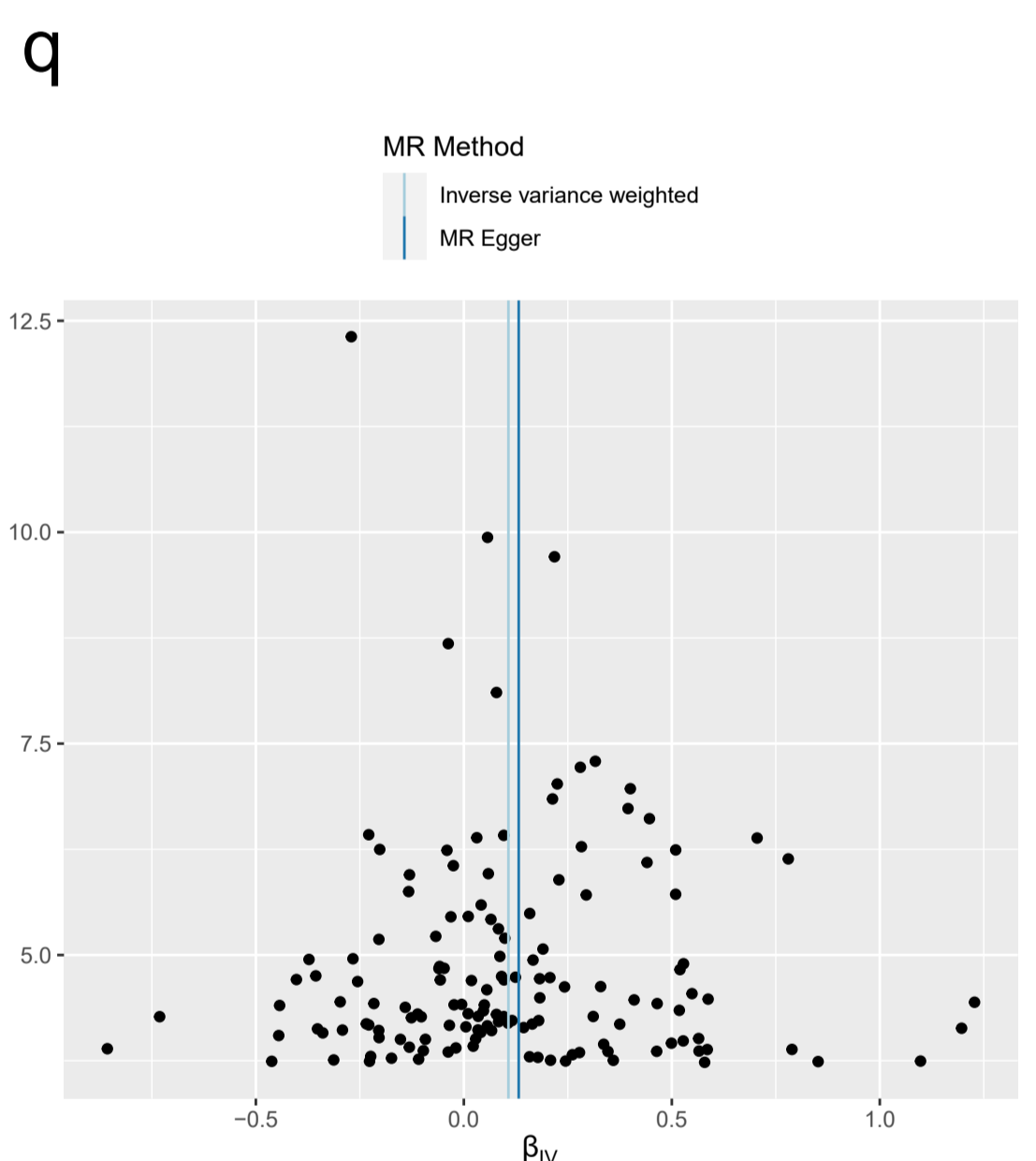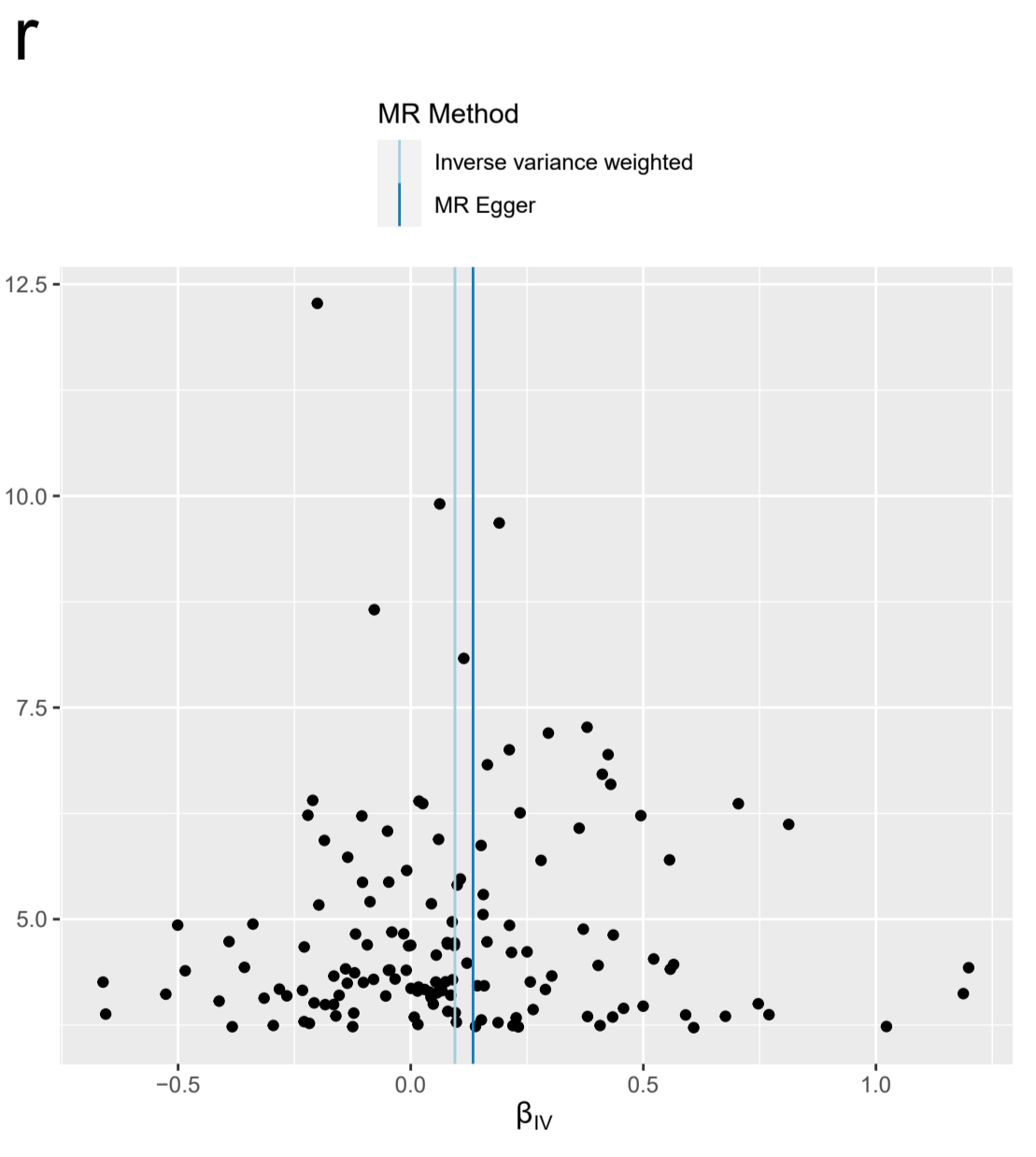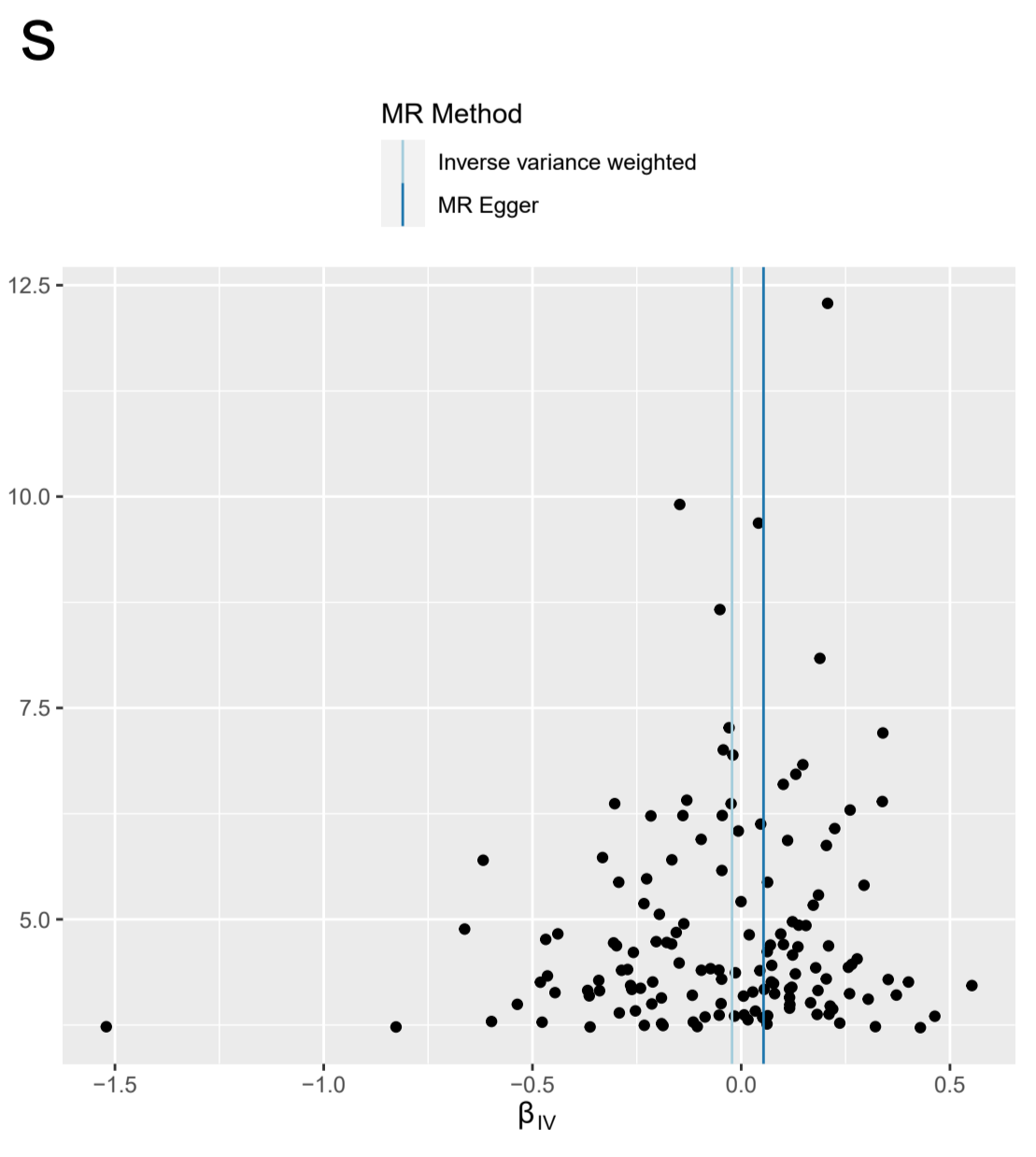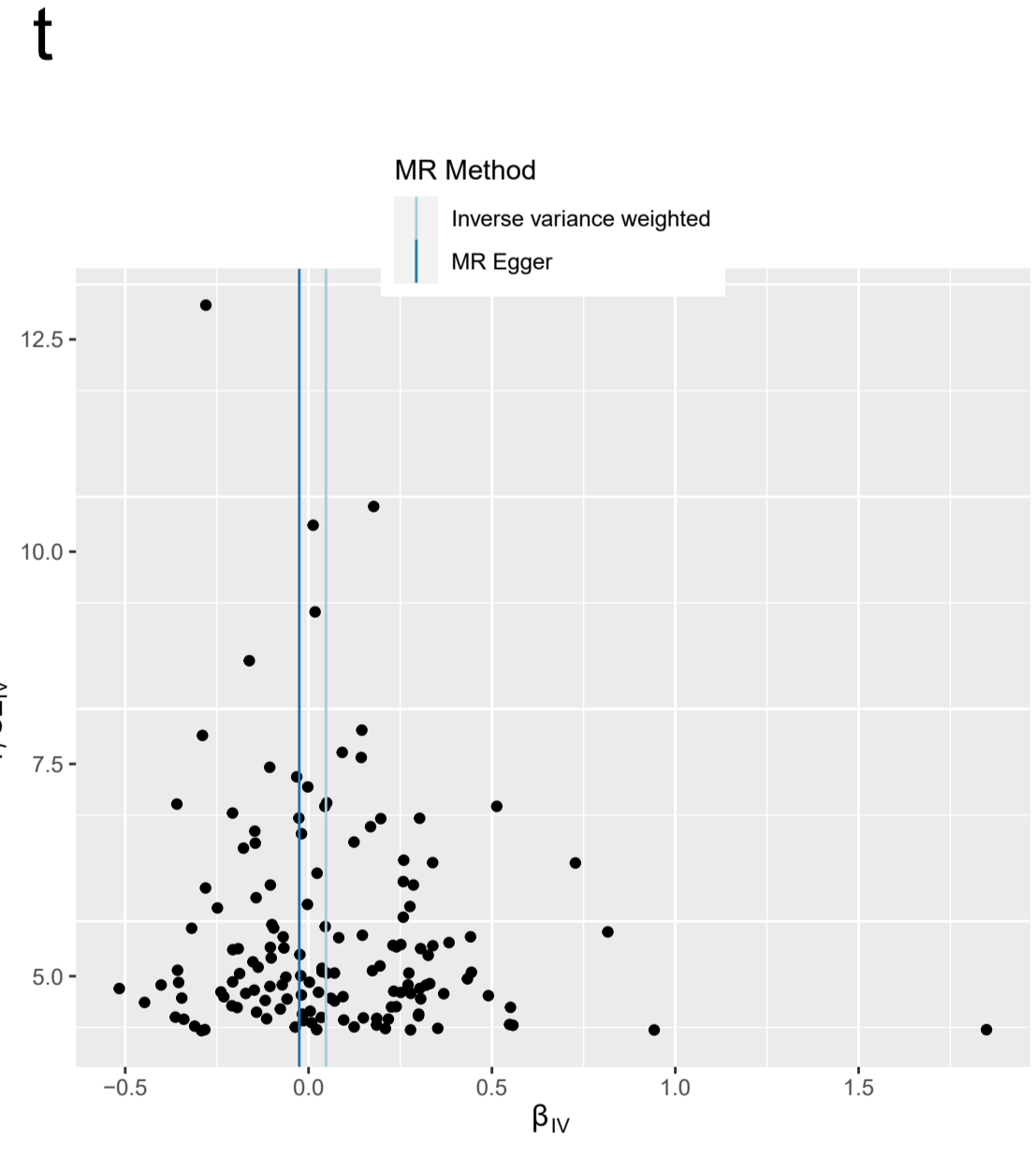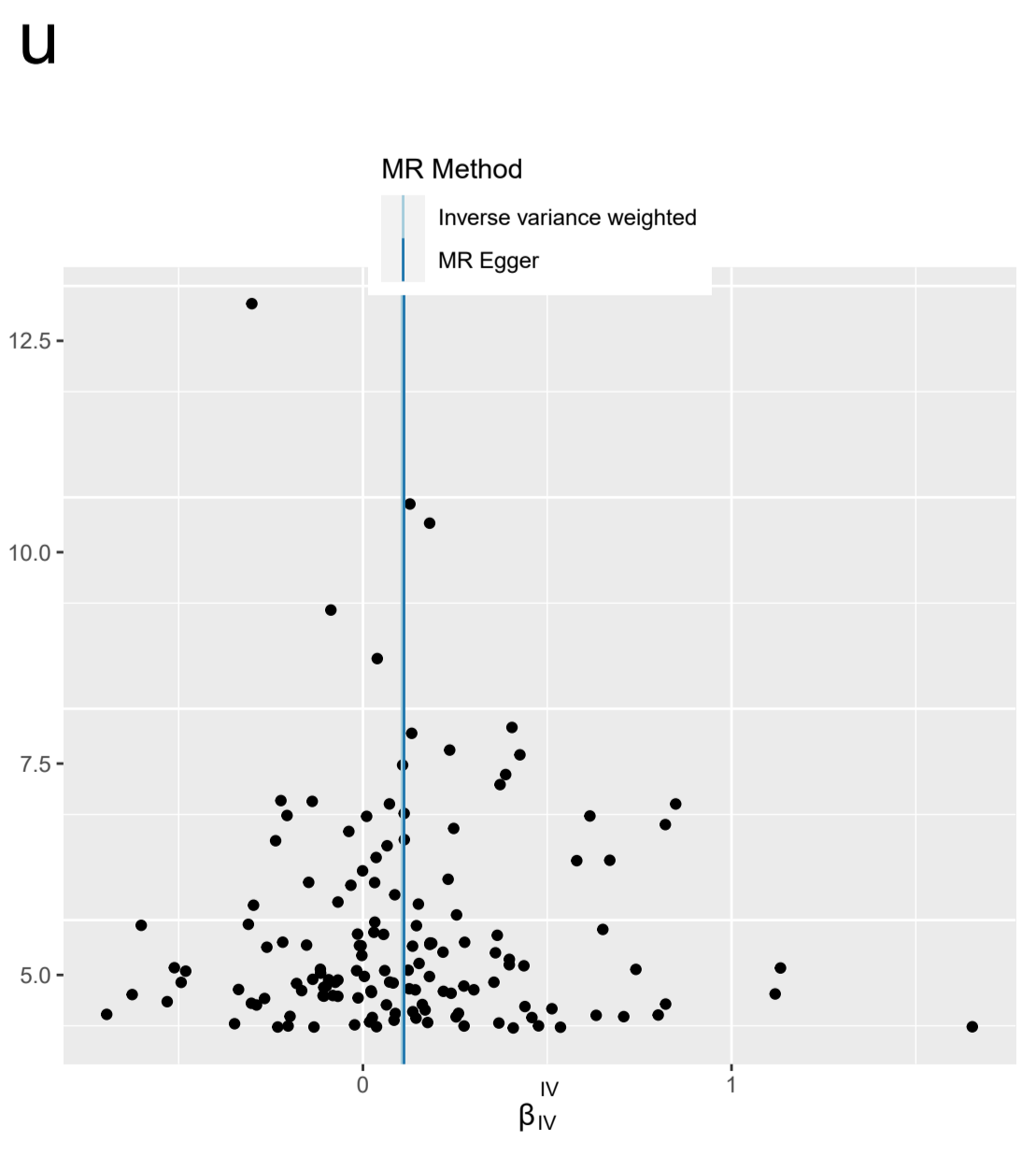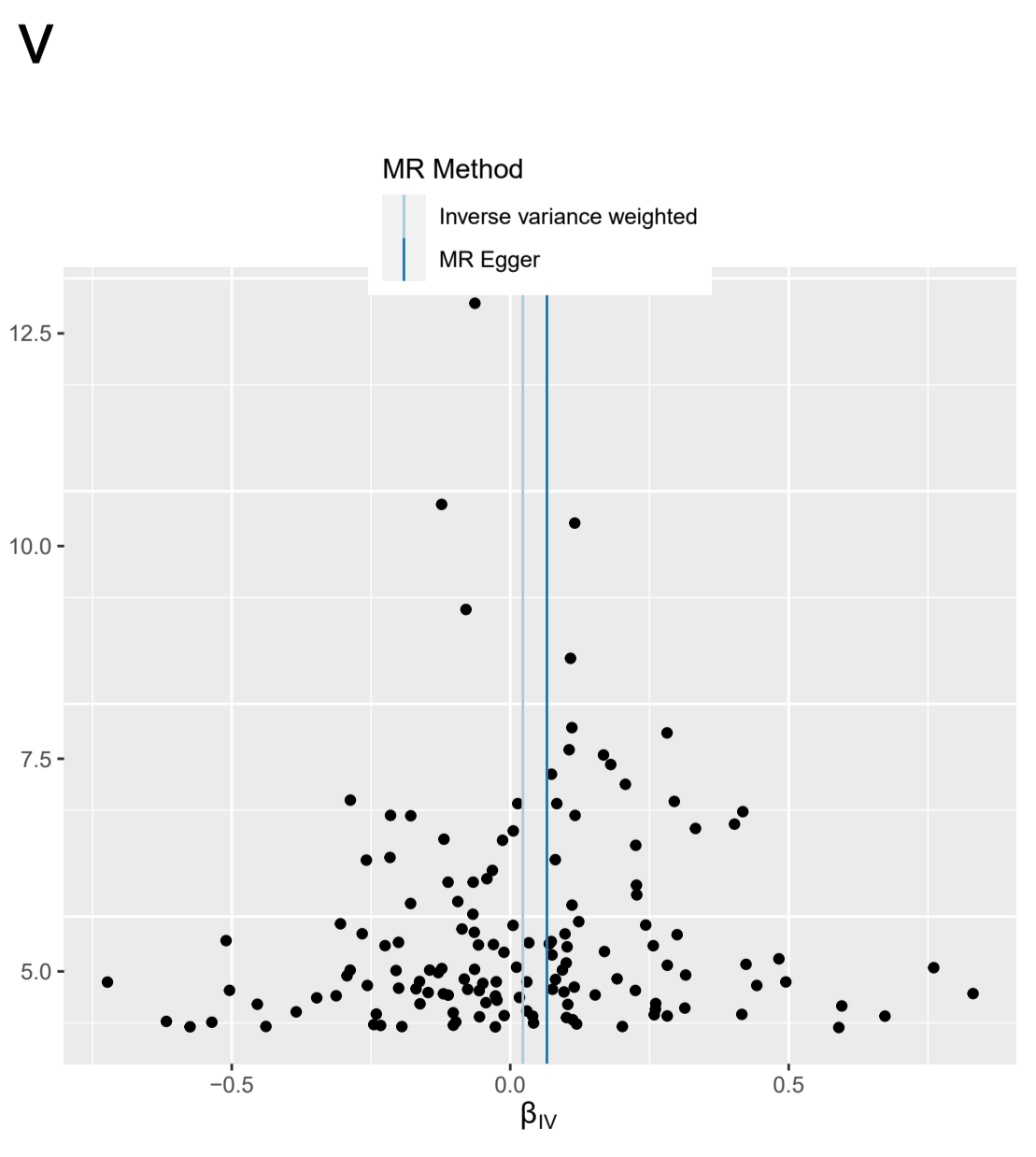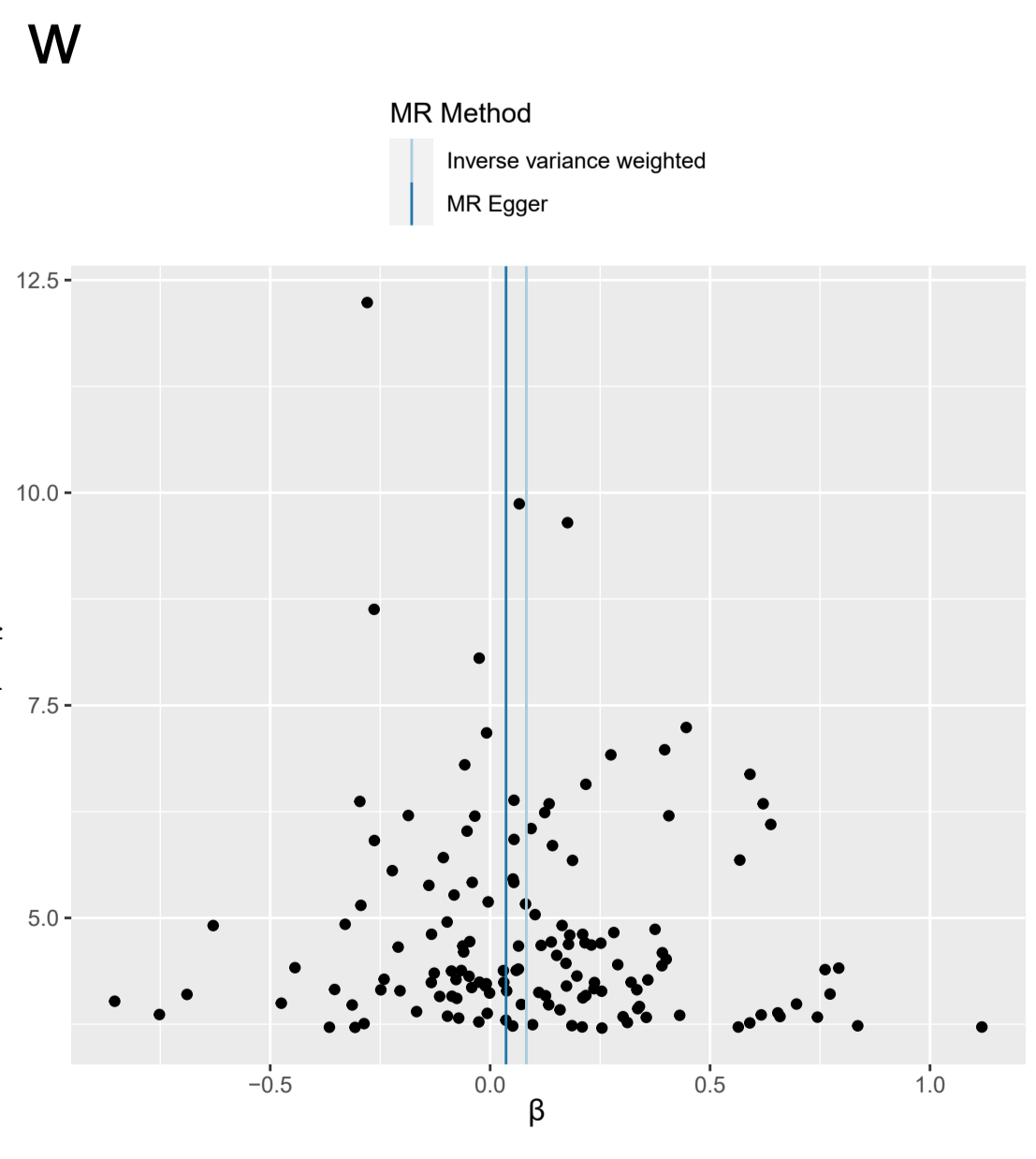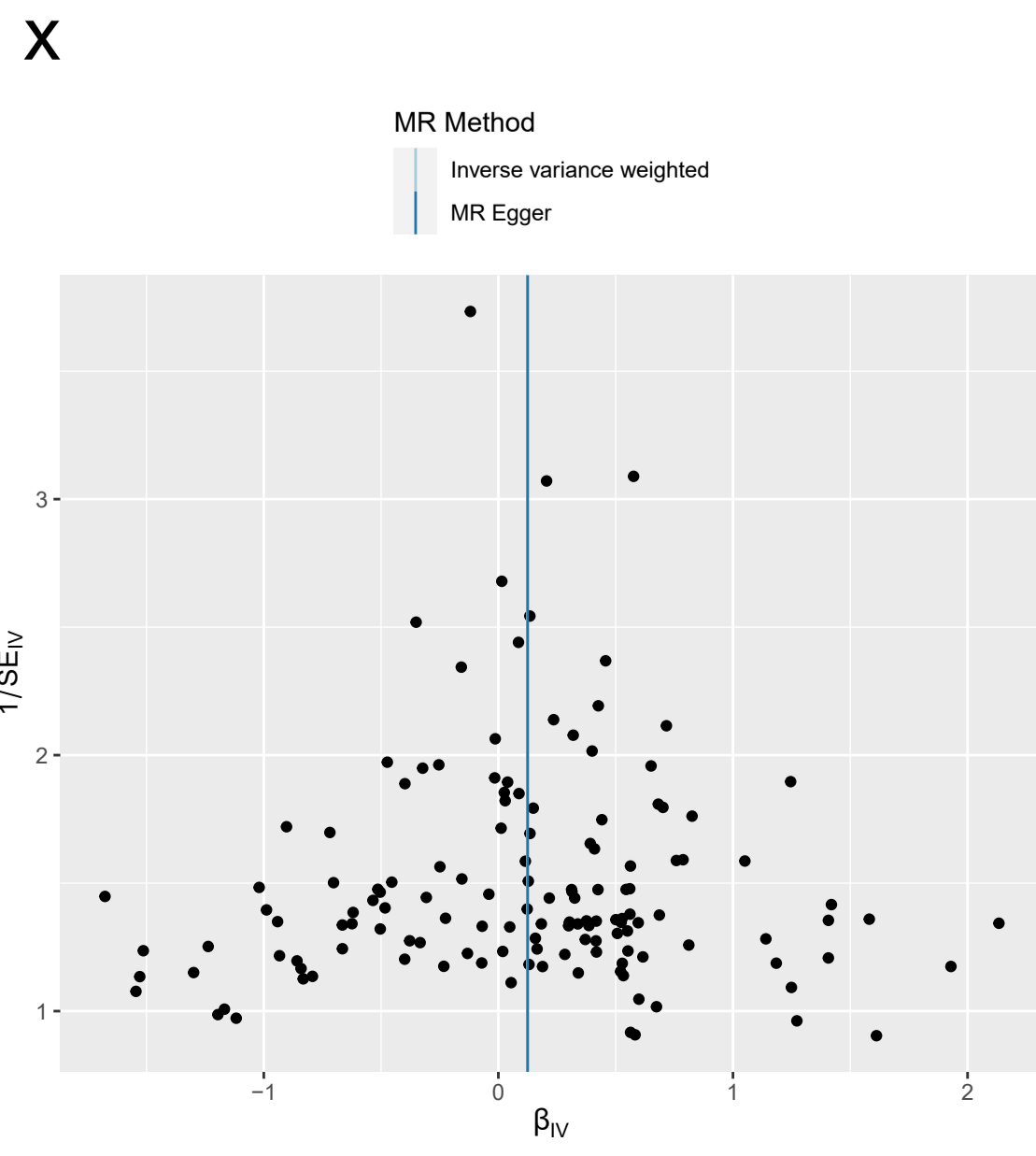

Supplement: Supplementary file 4 — Supplementary Material 4 [file 12944_2024_2087_MOESM4_ESM.pdf]

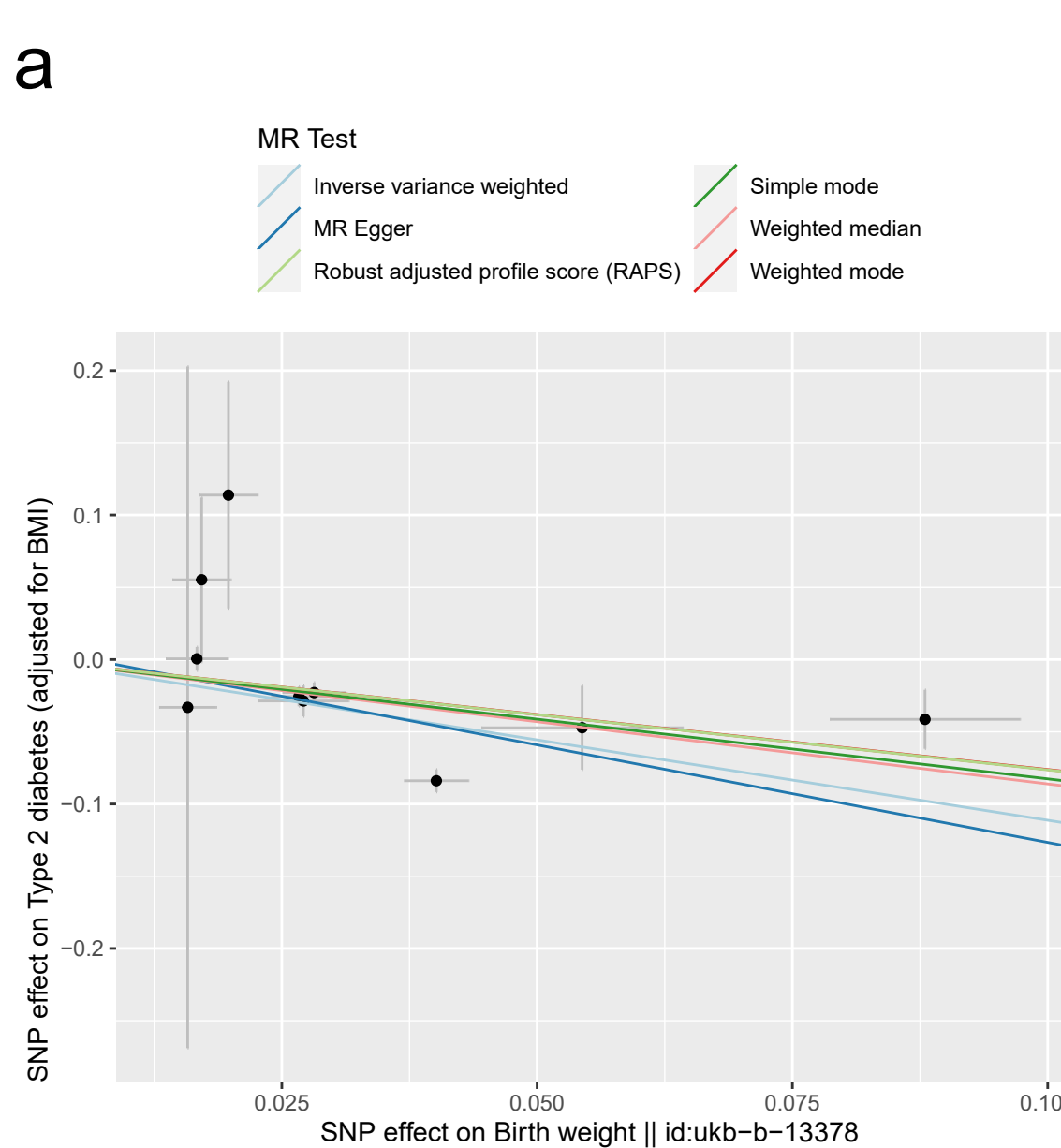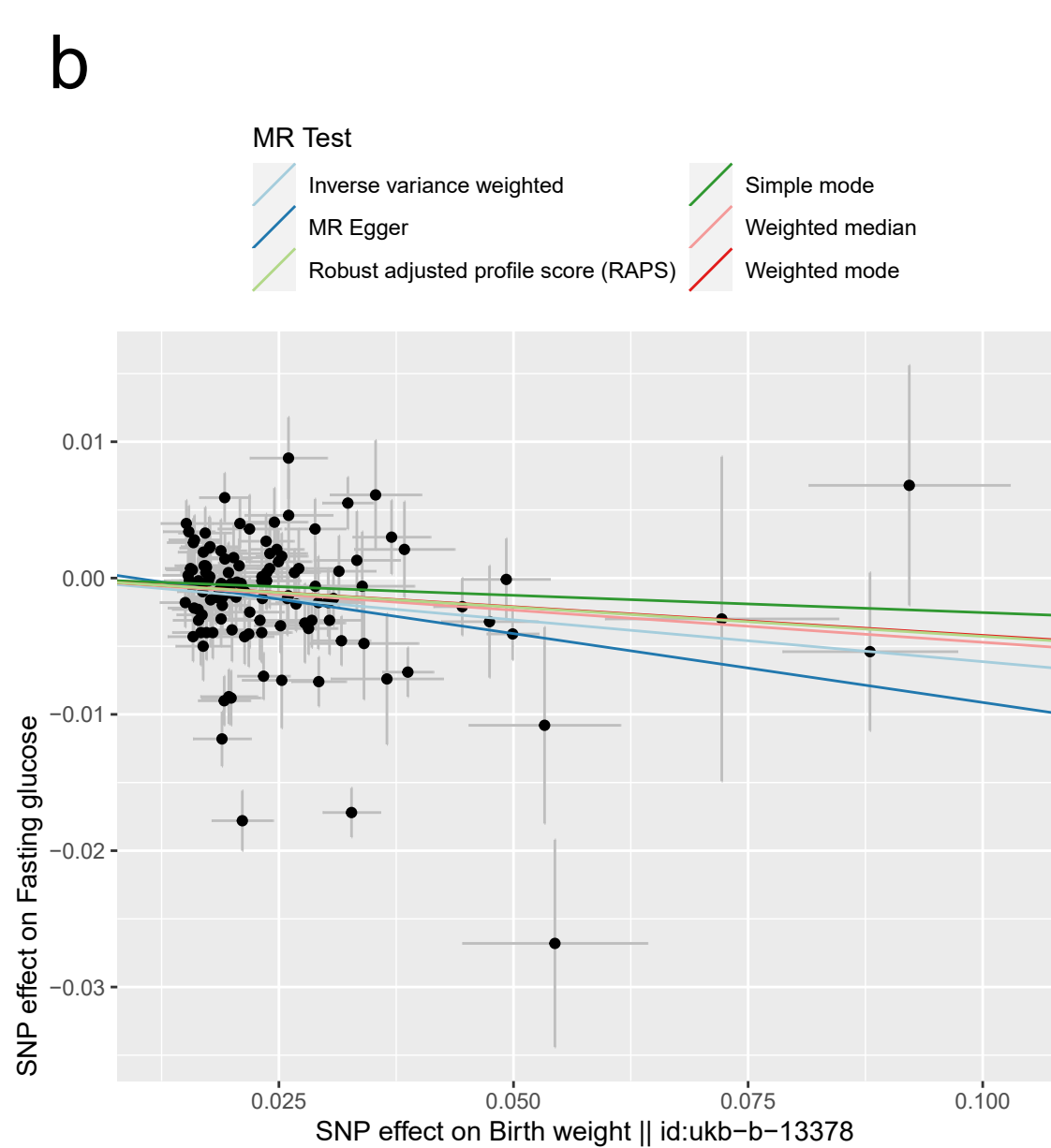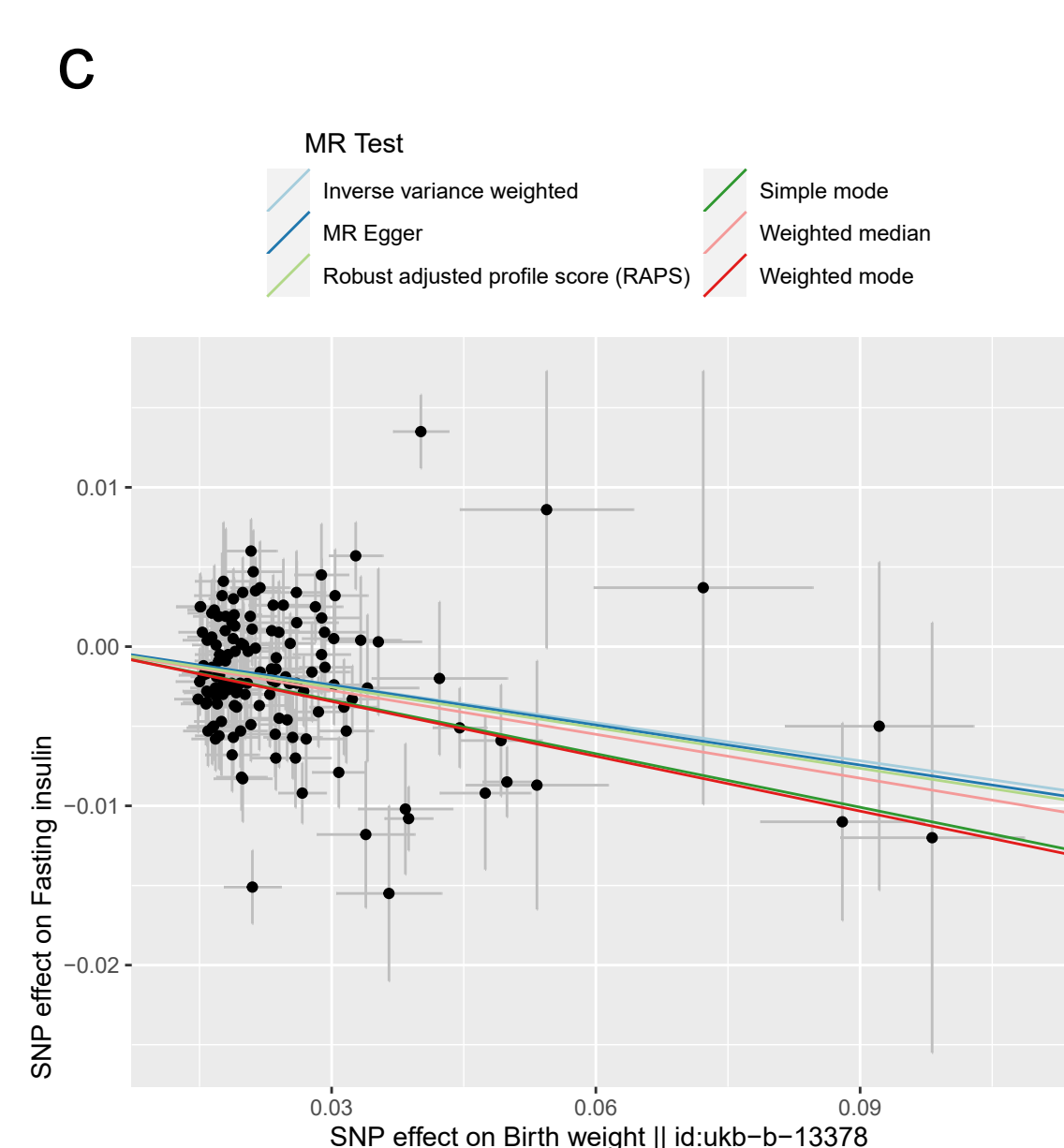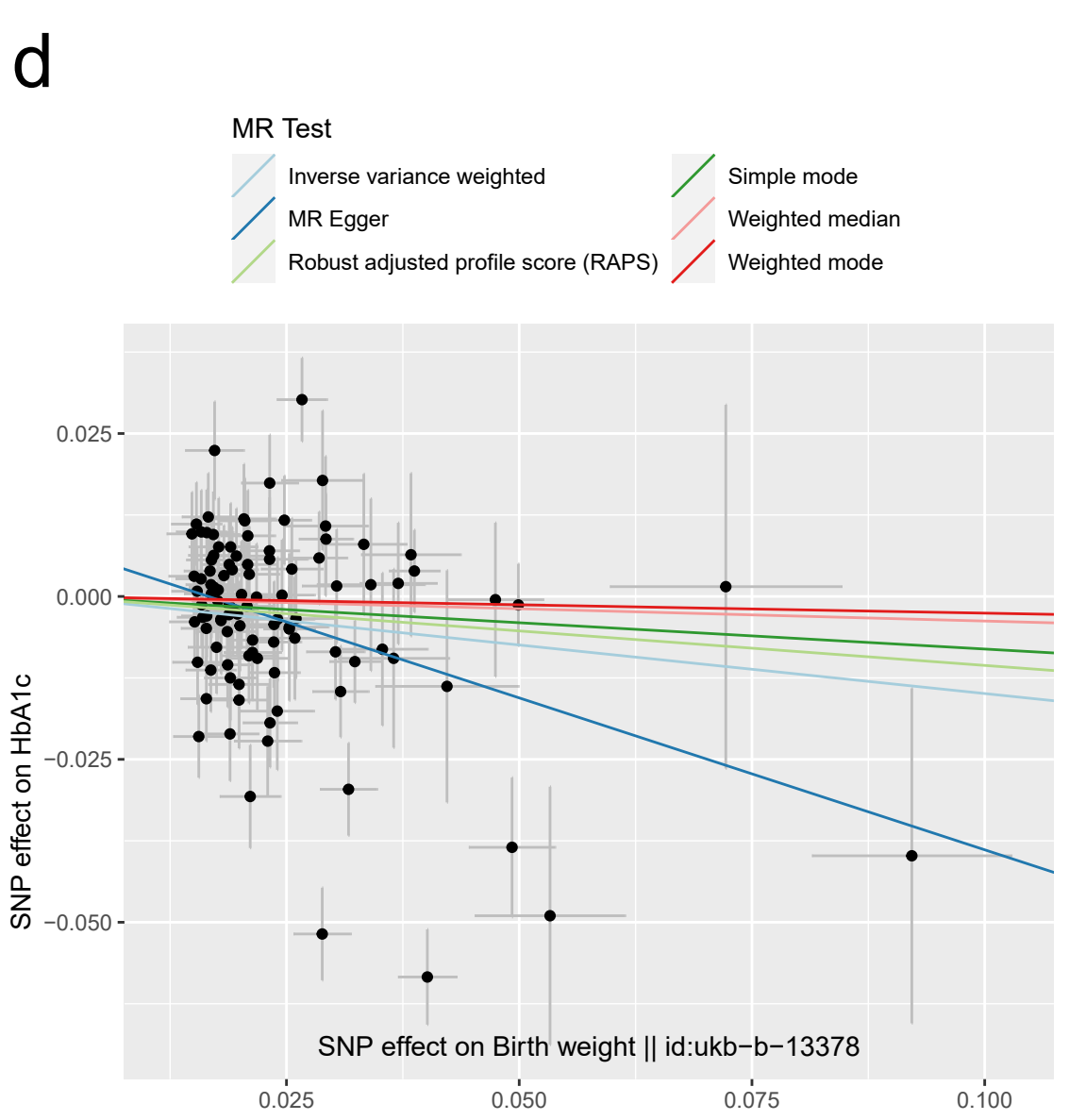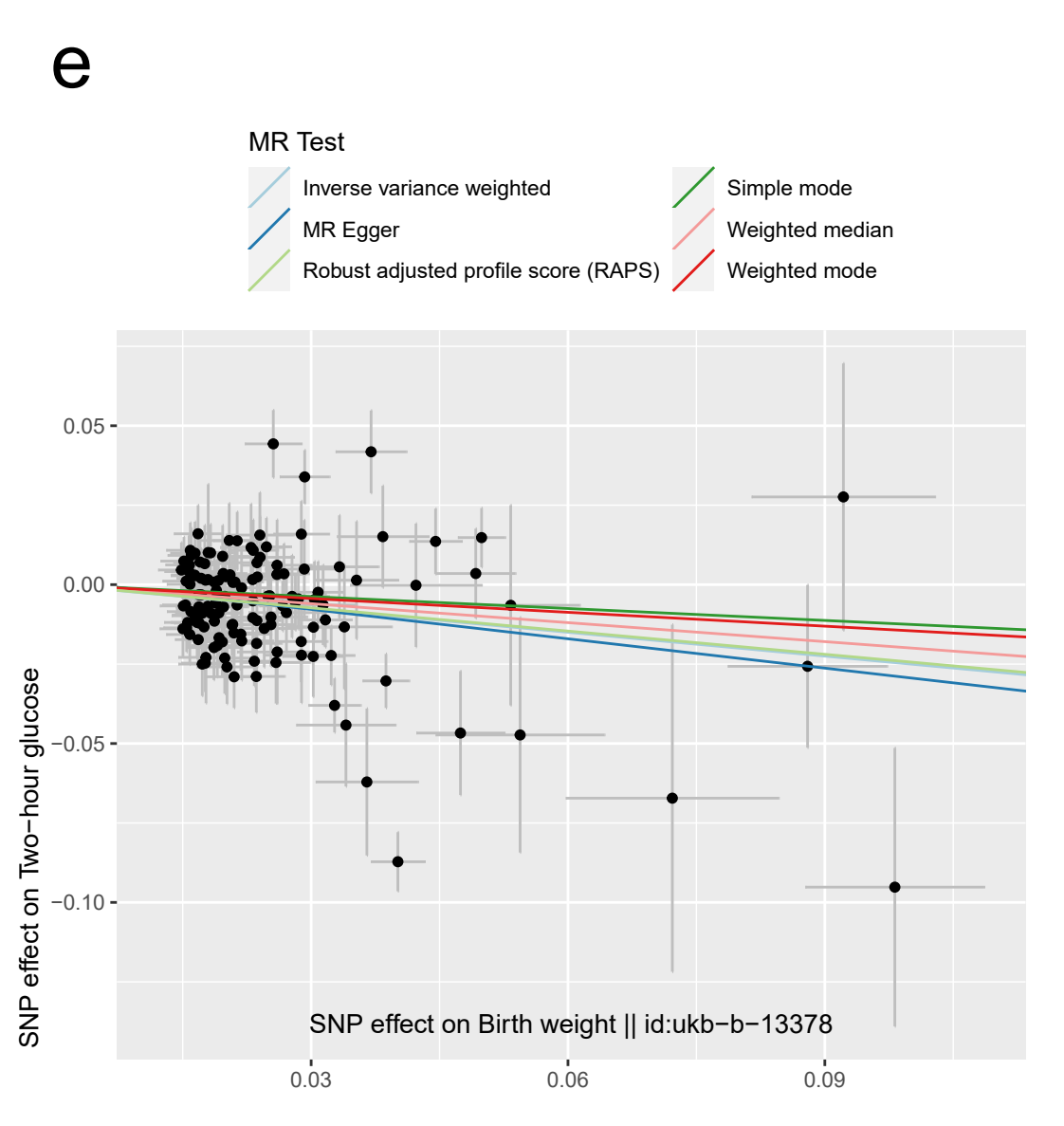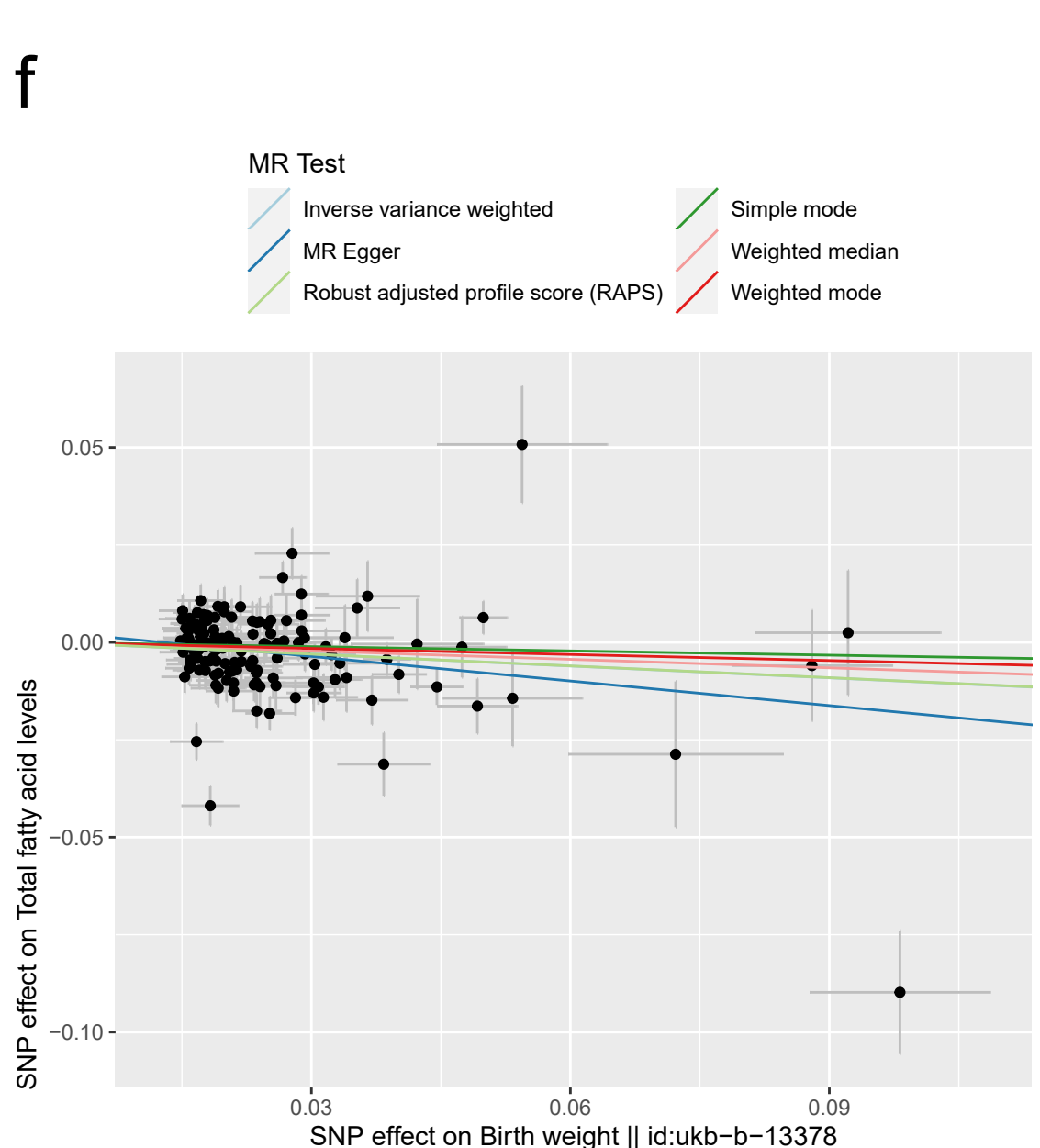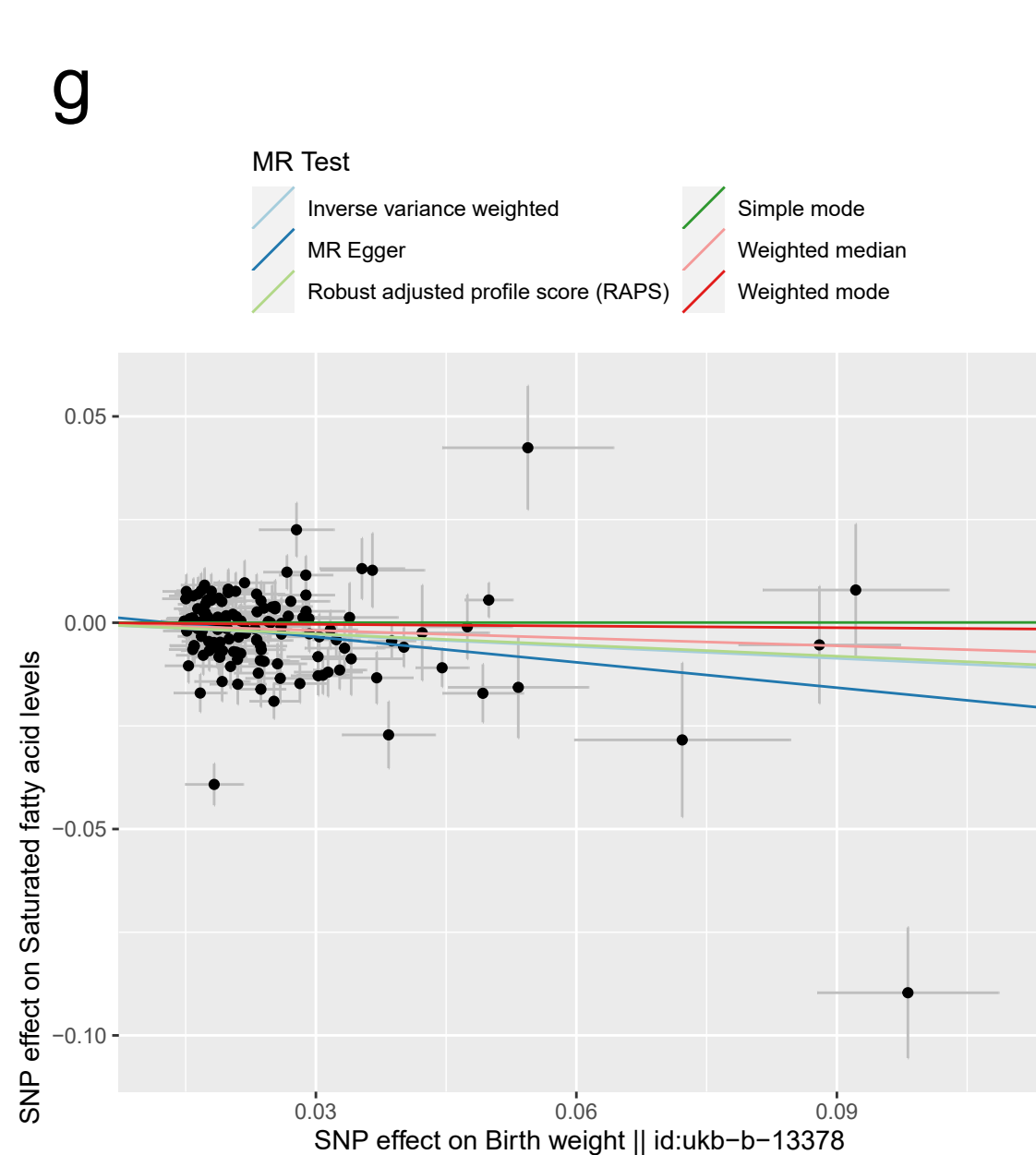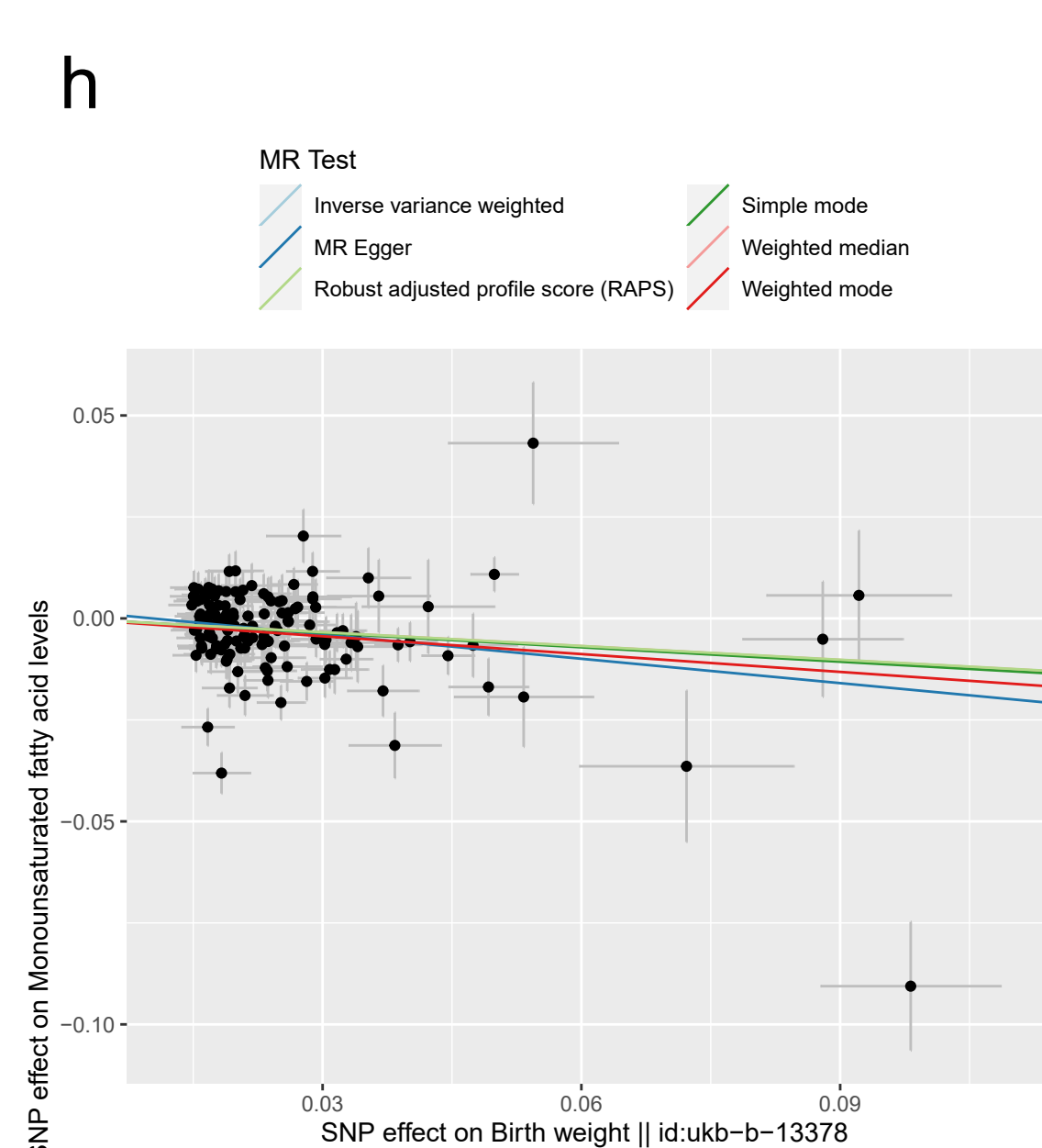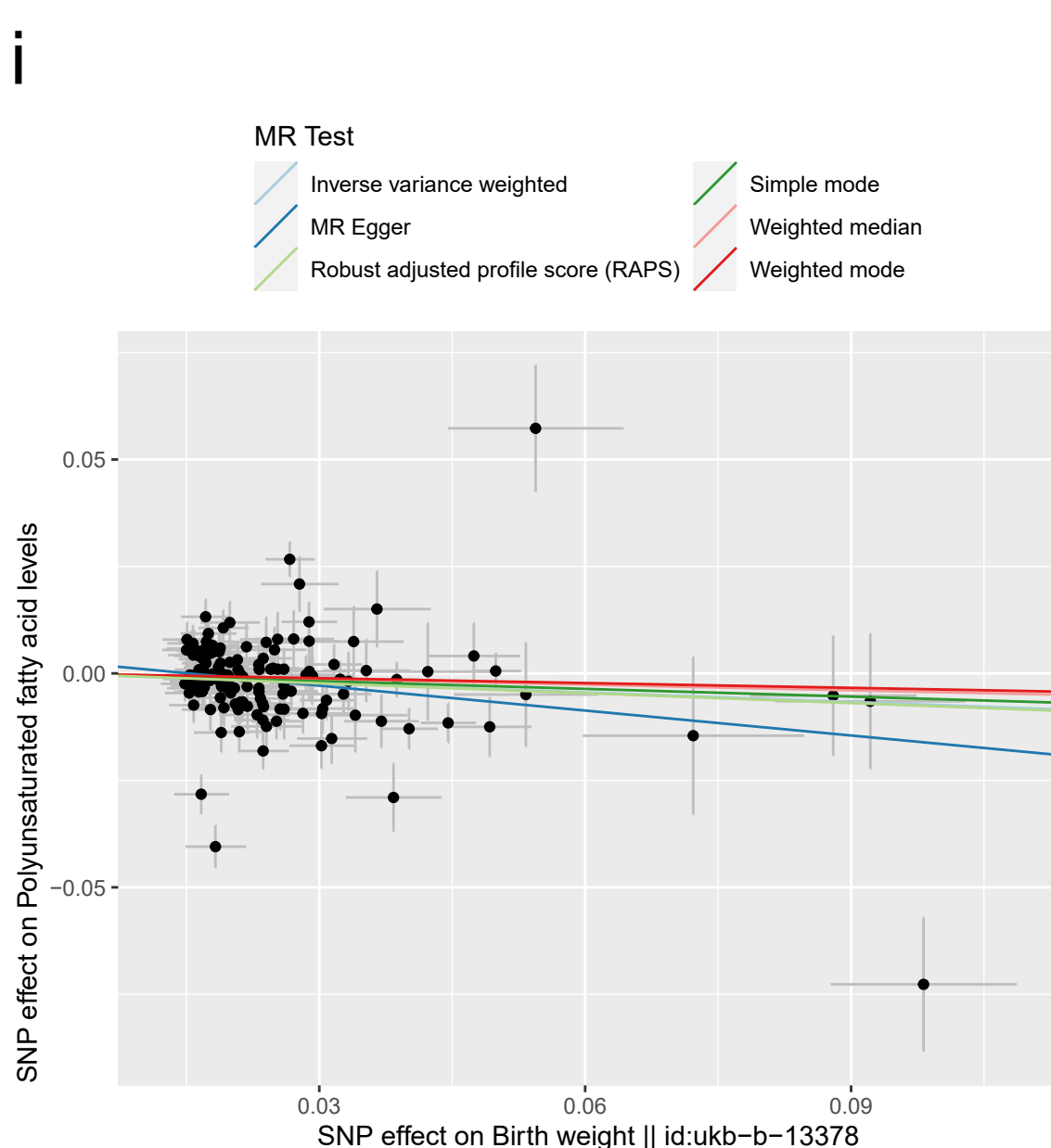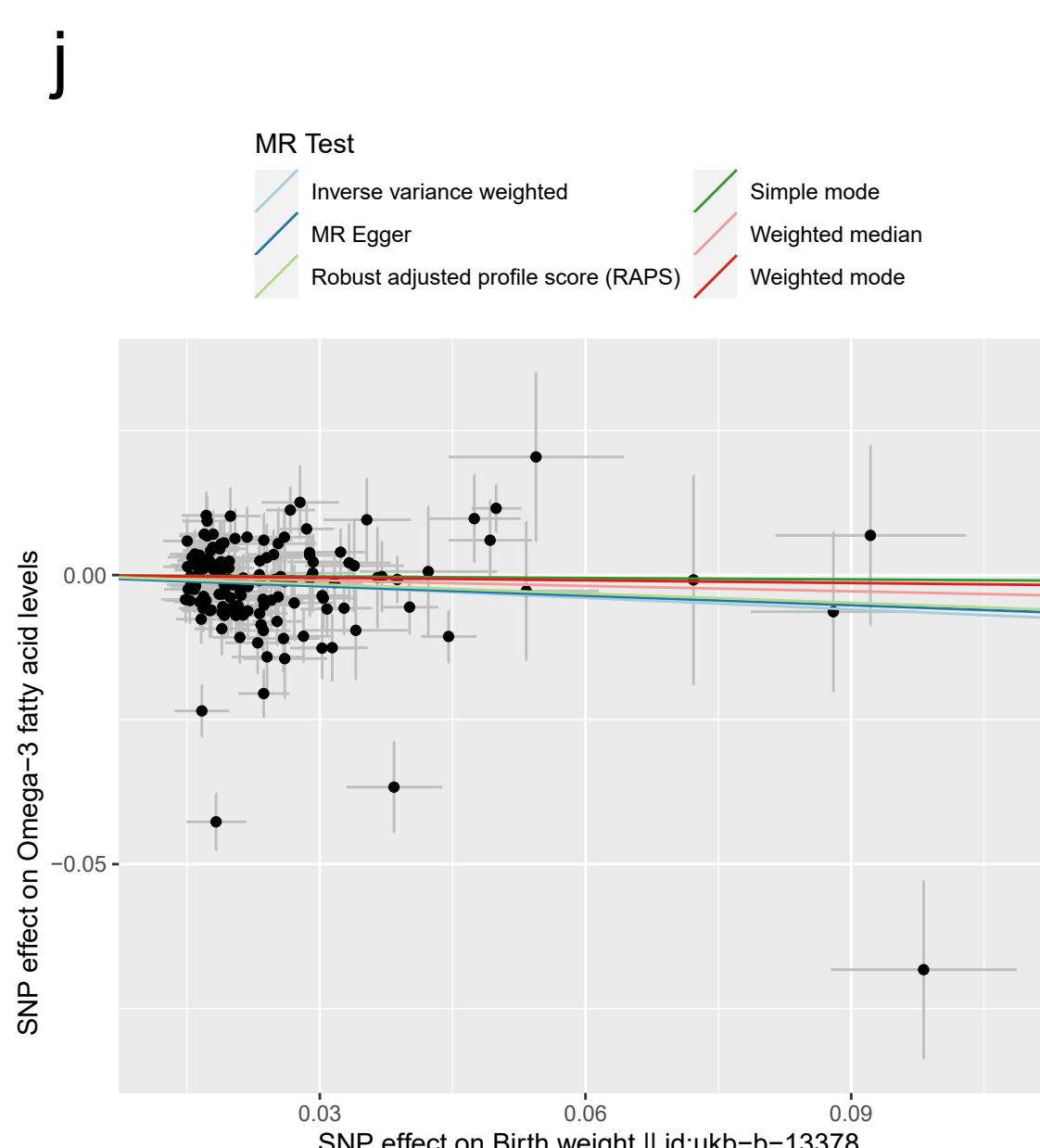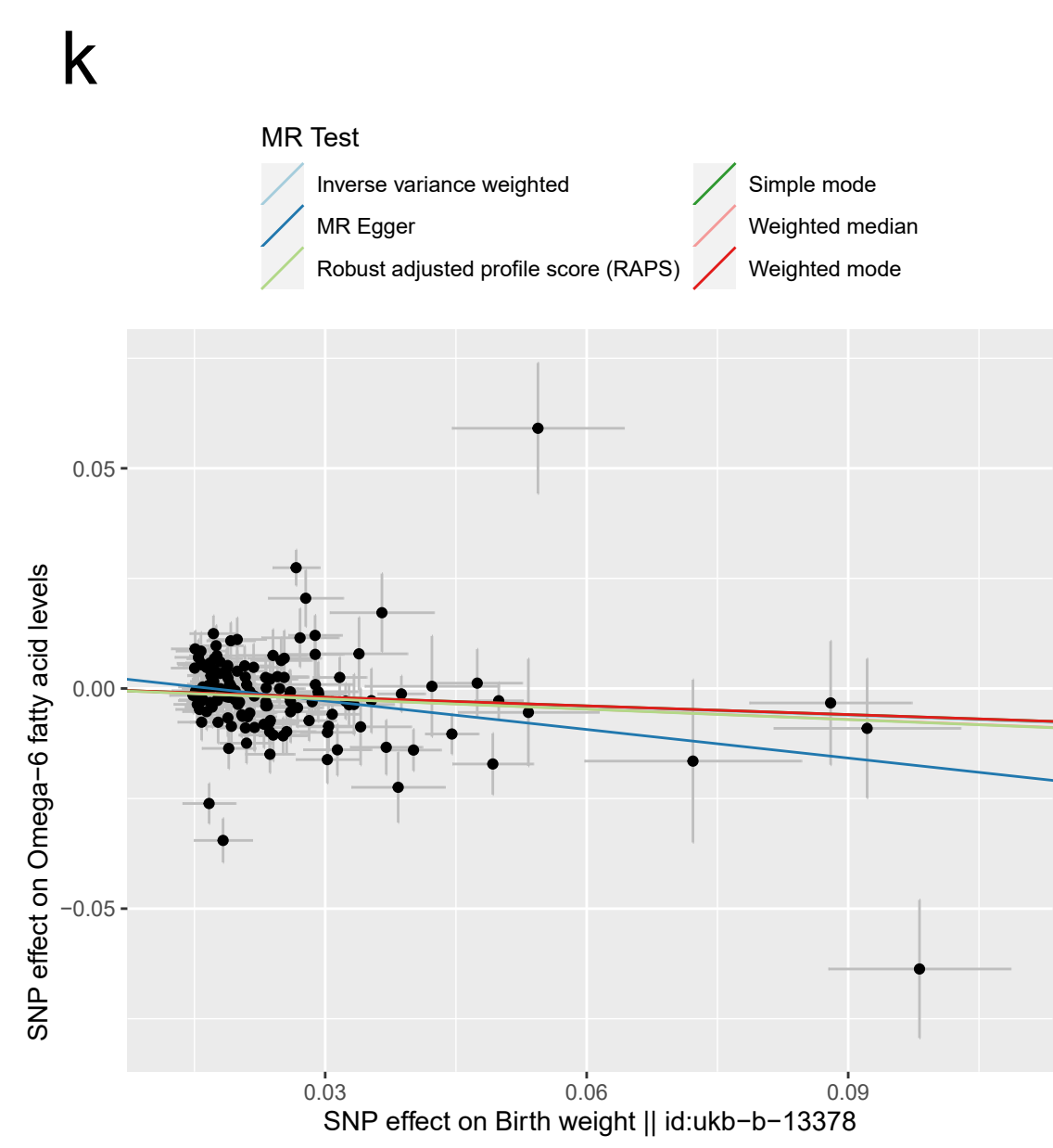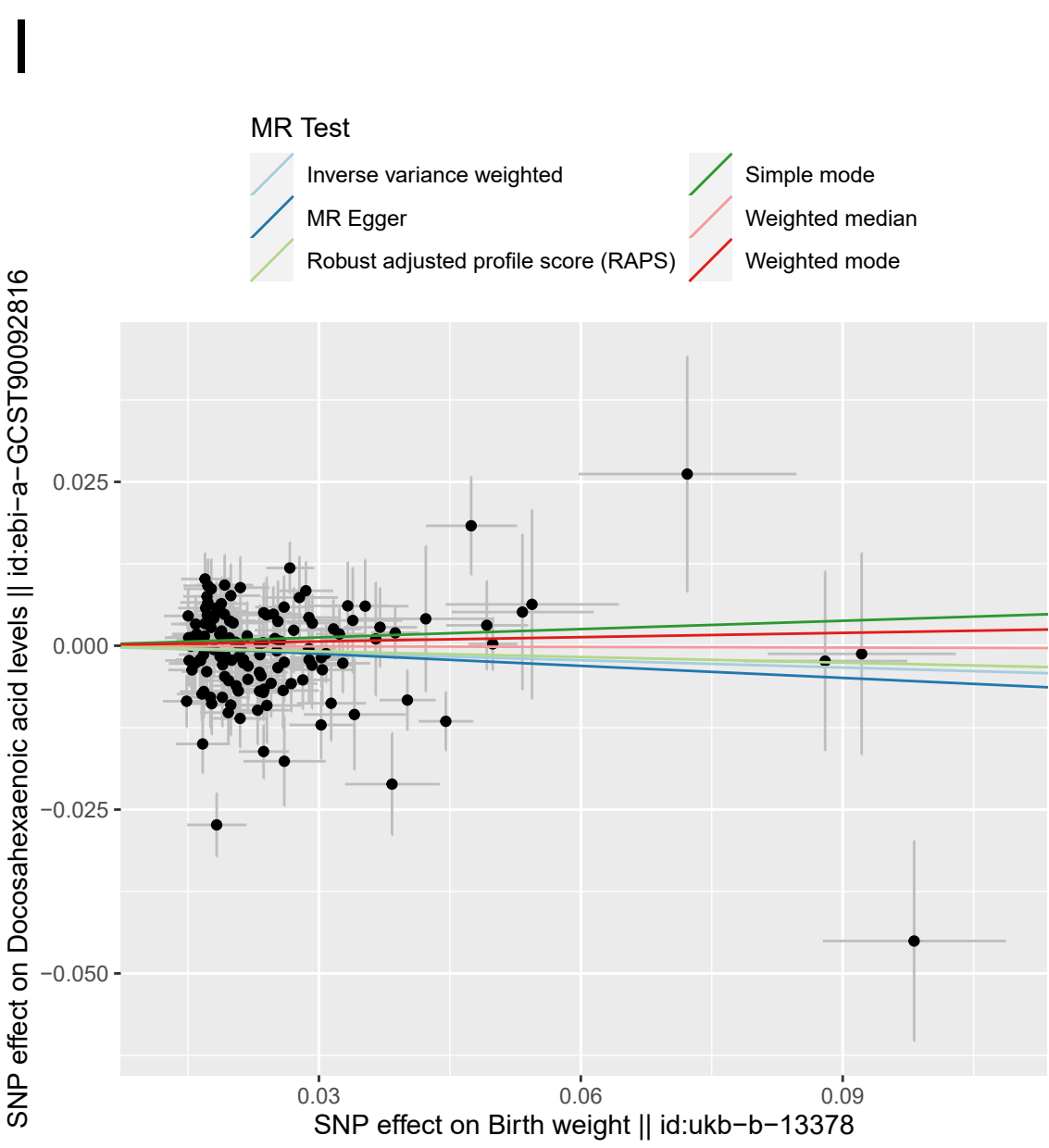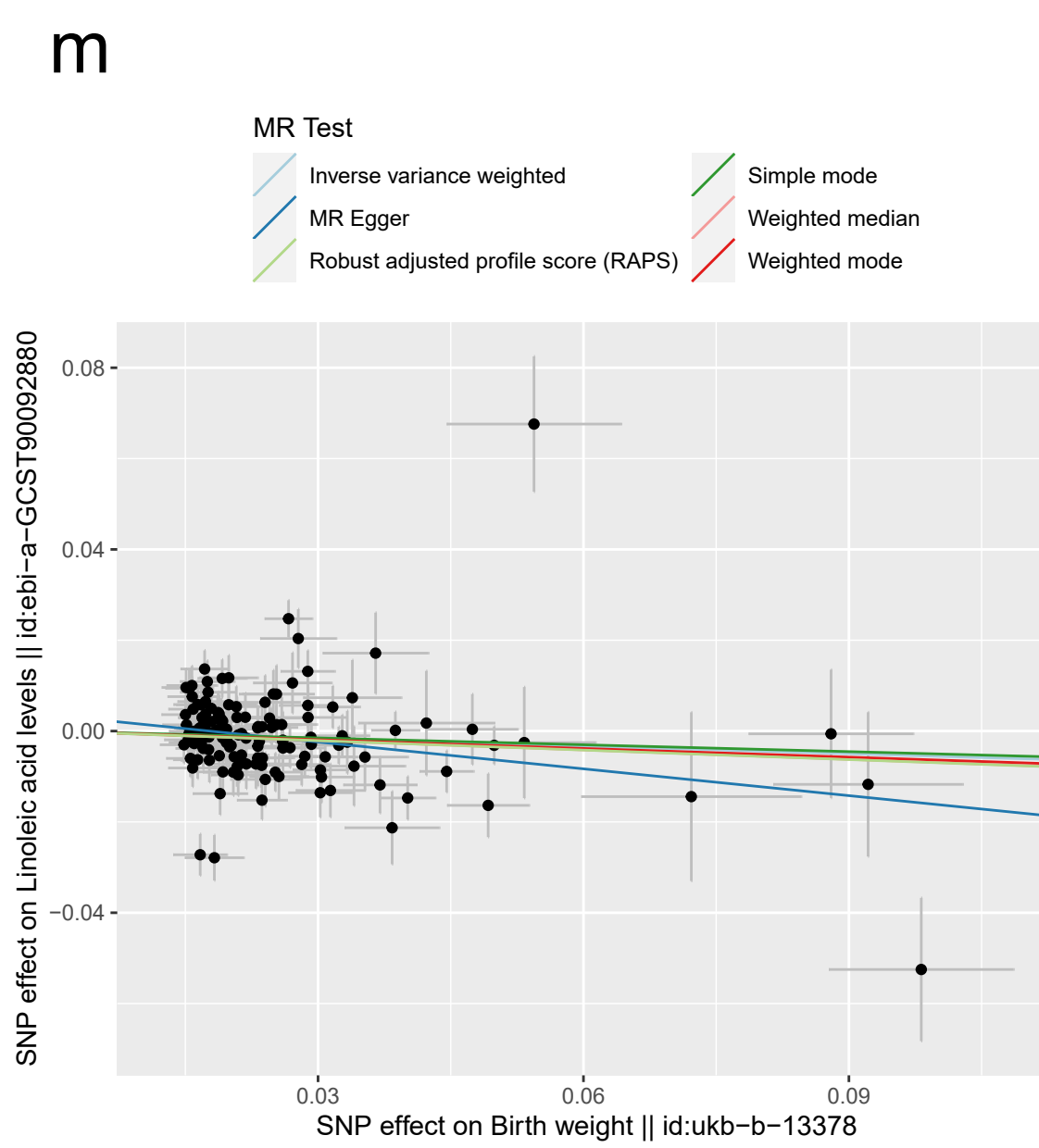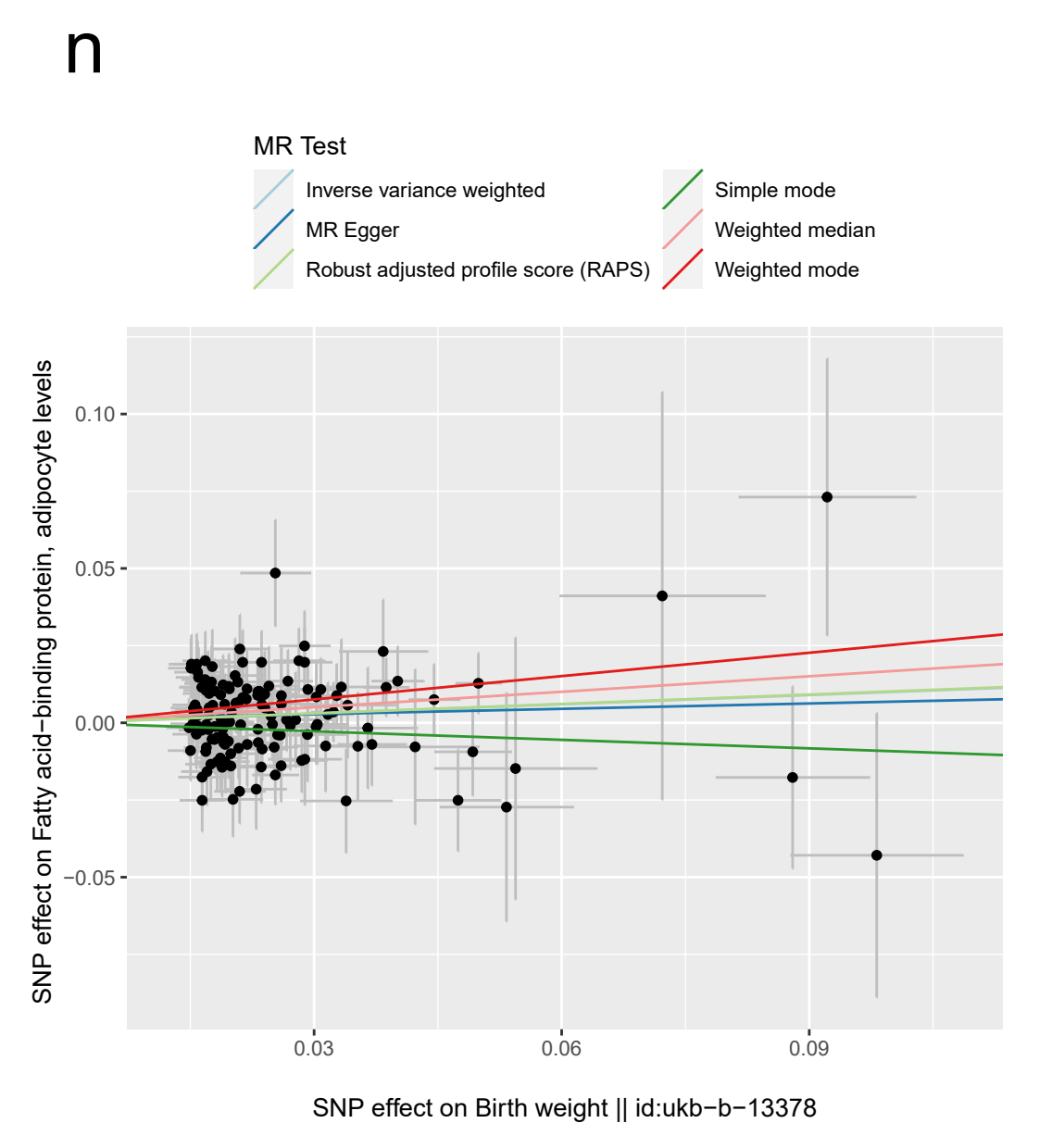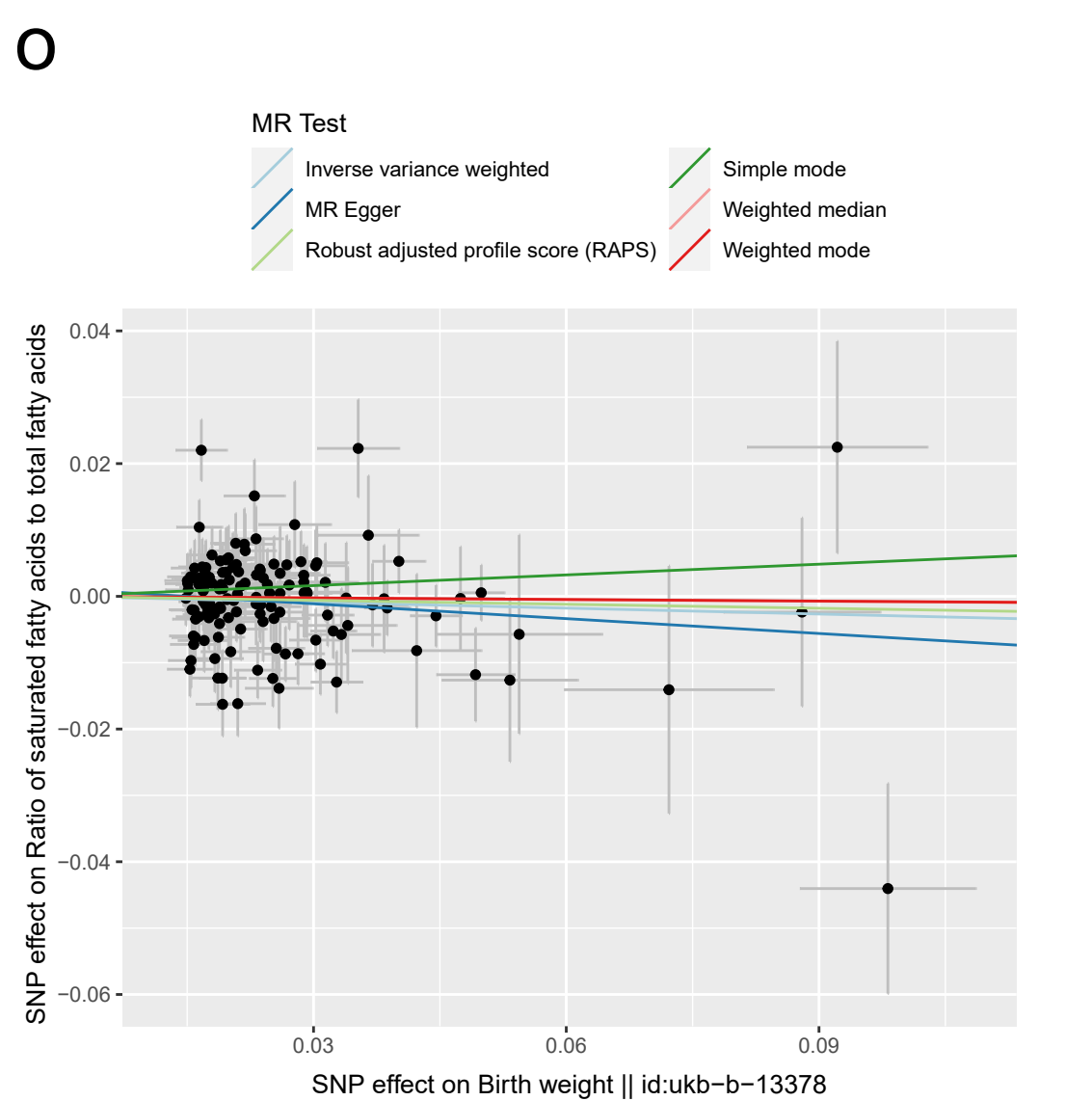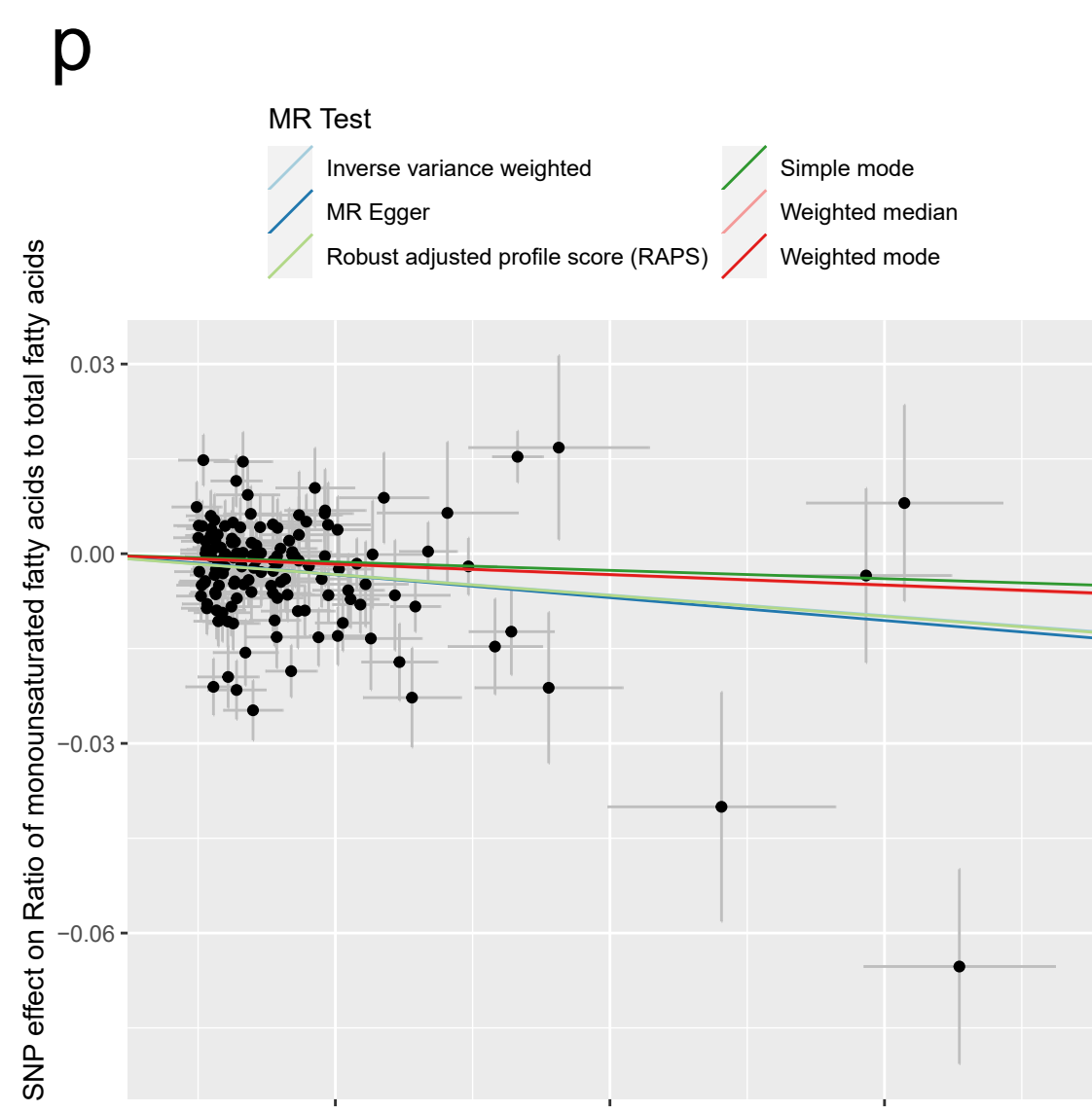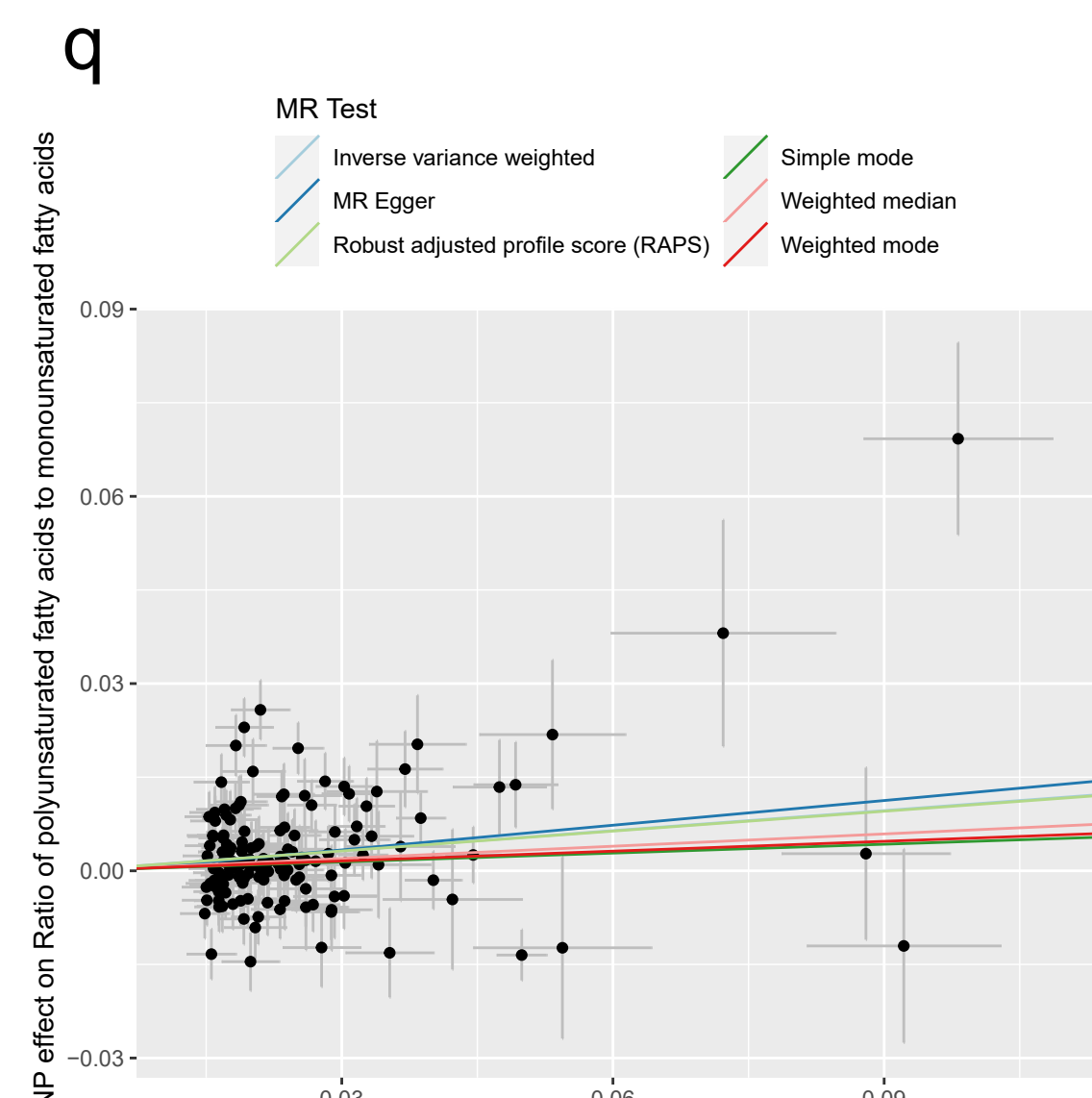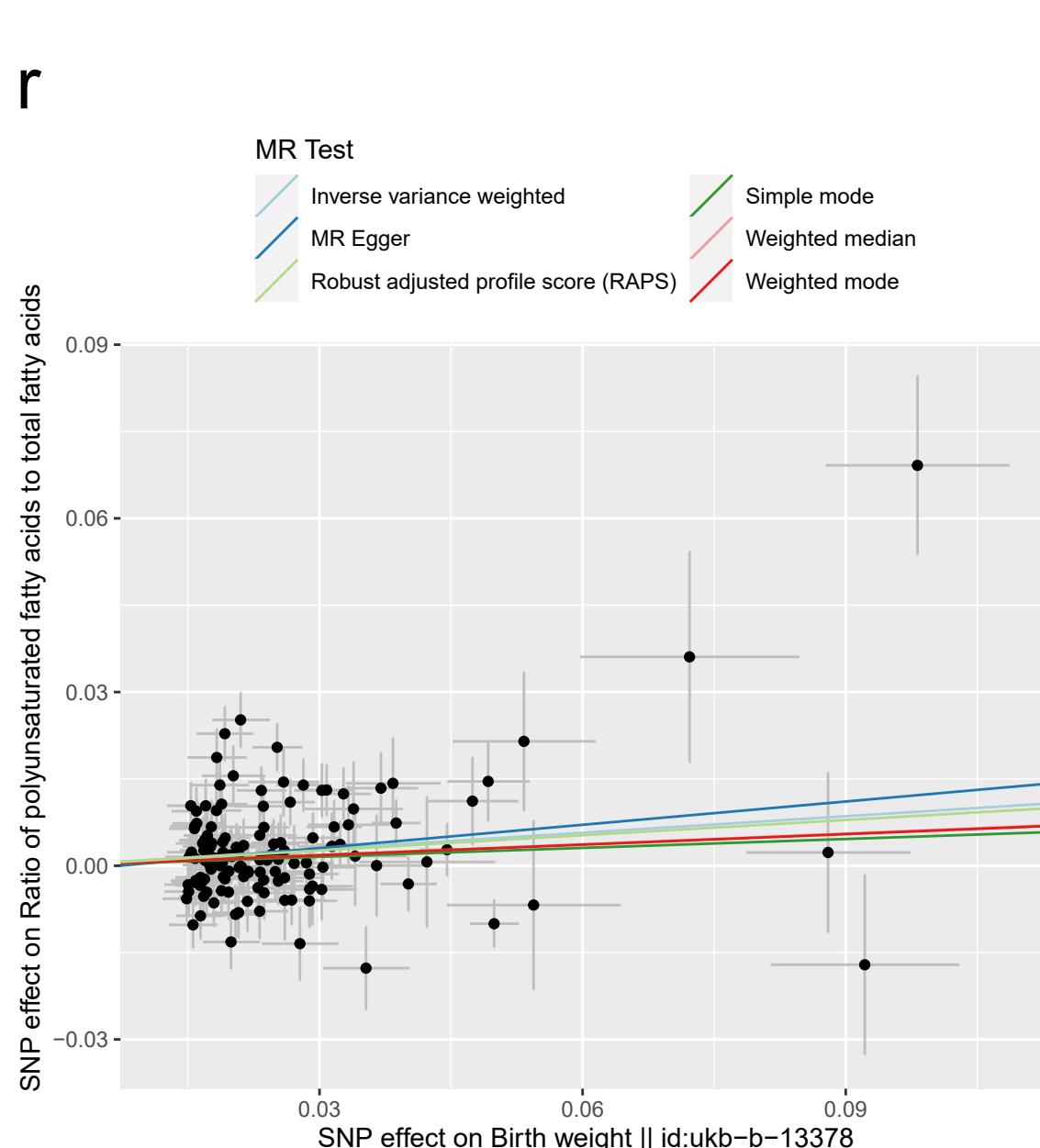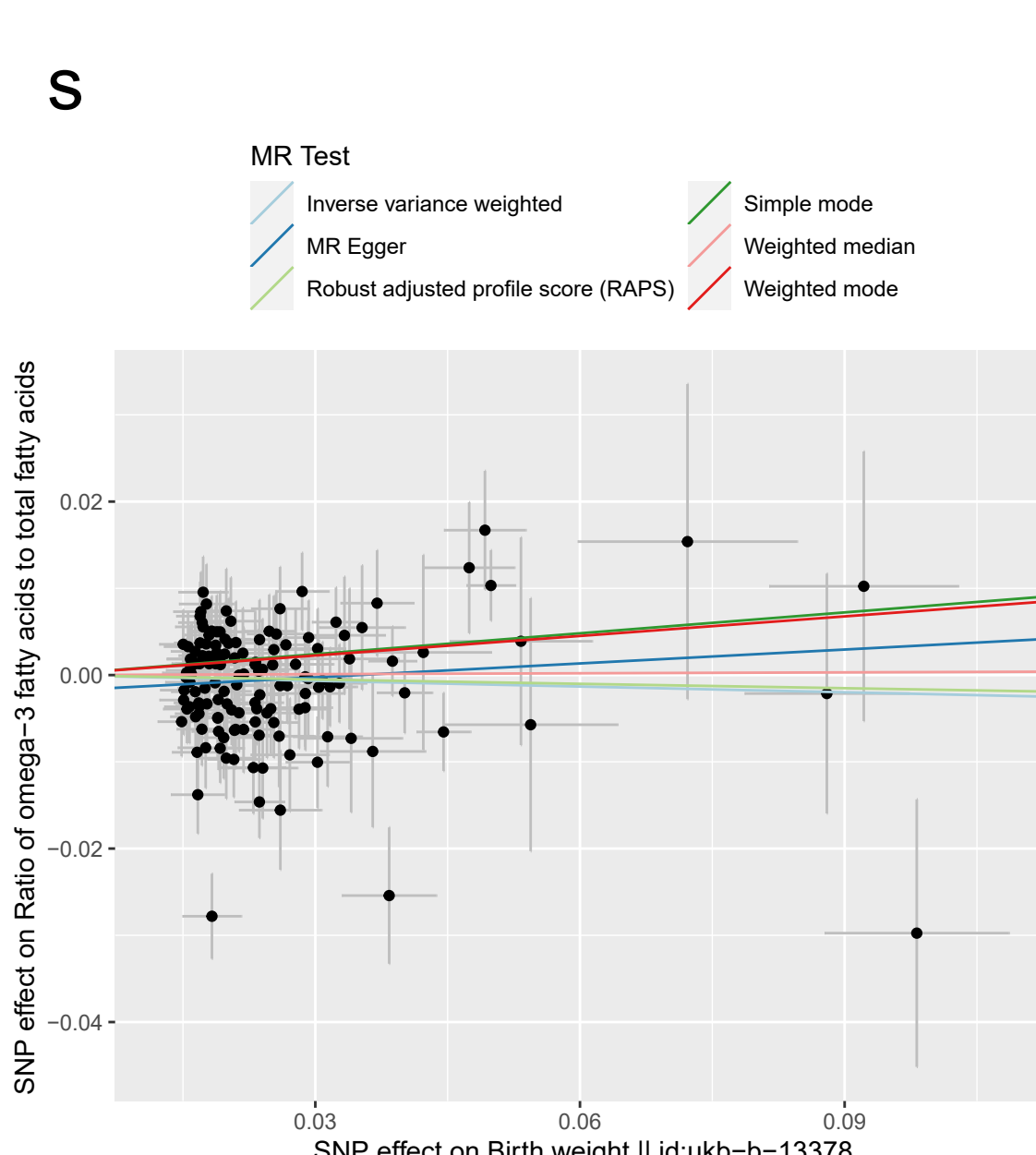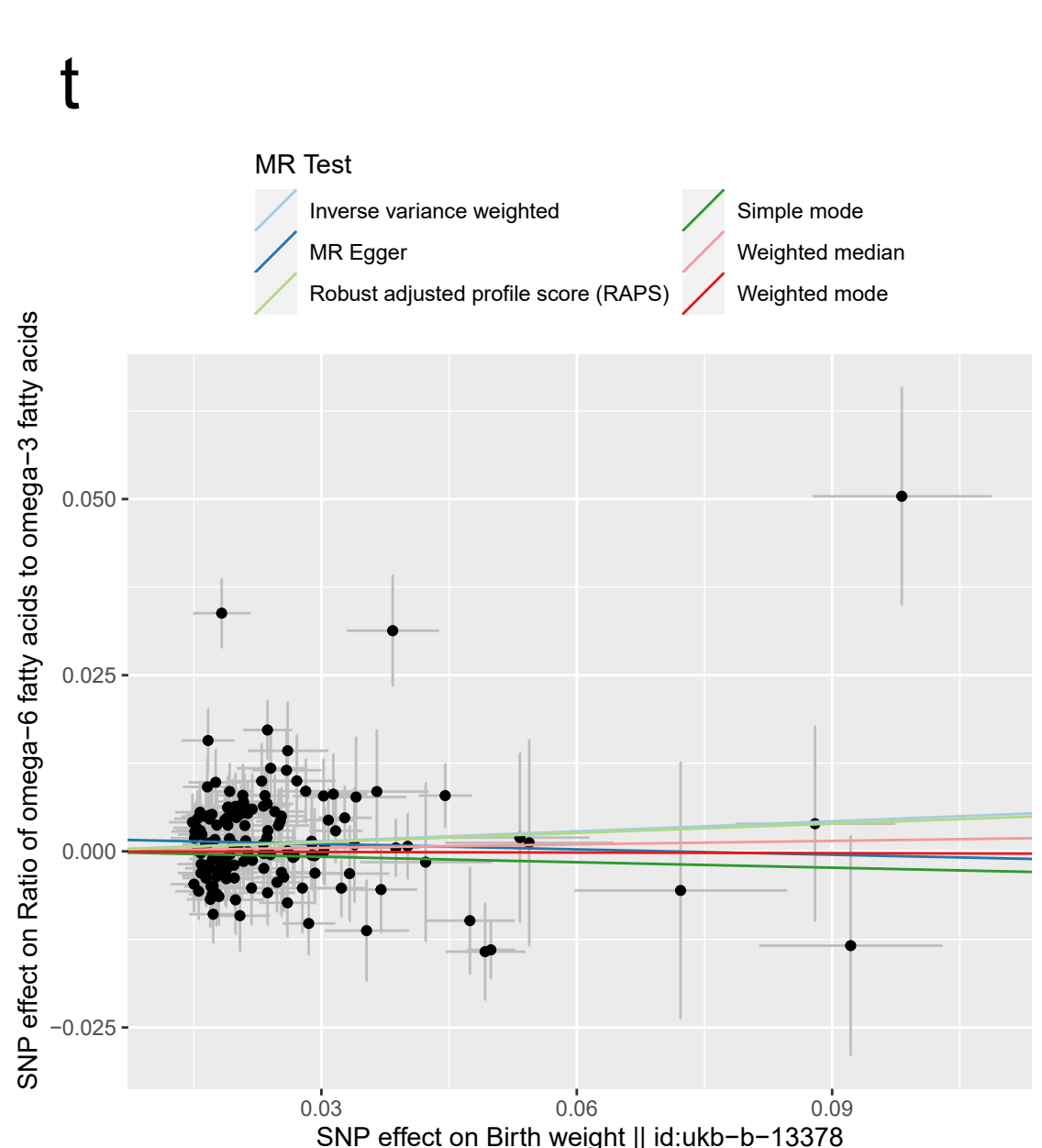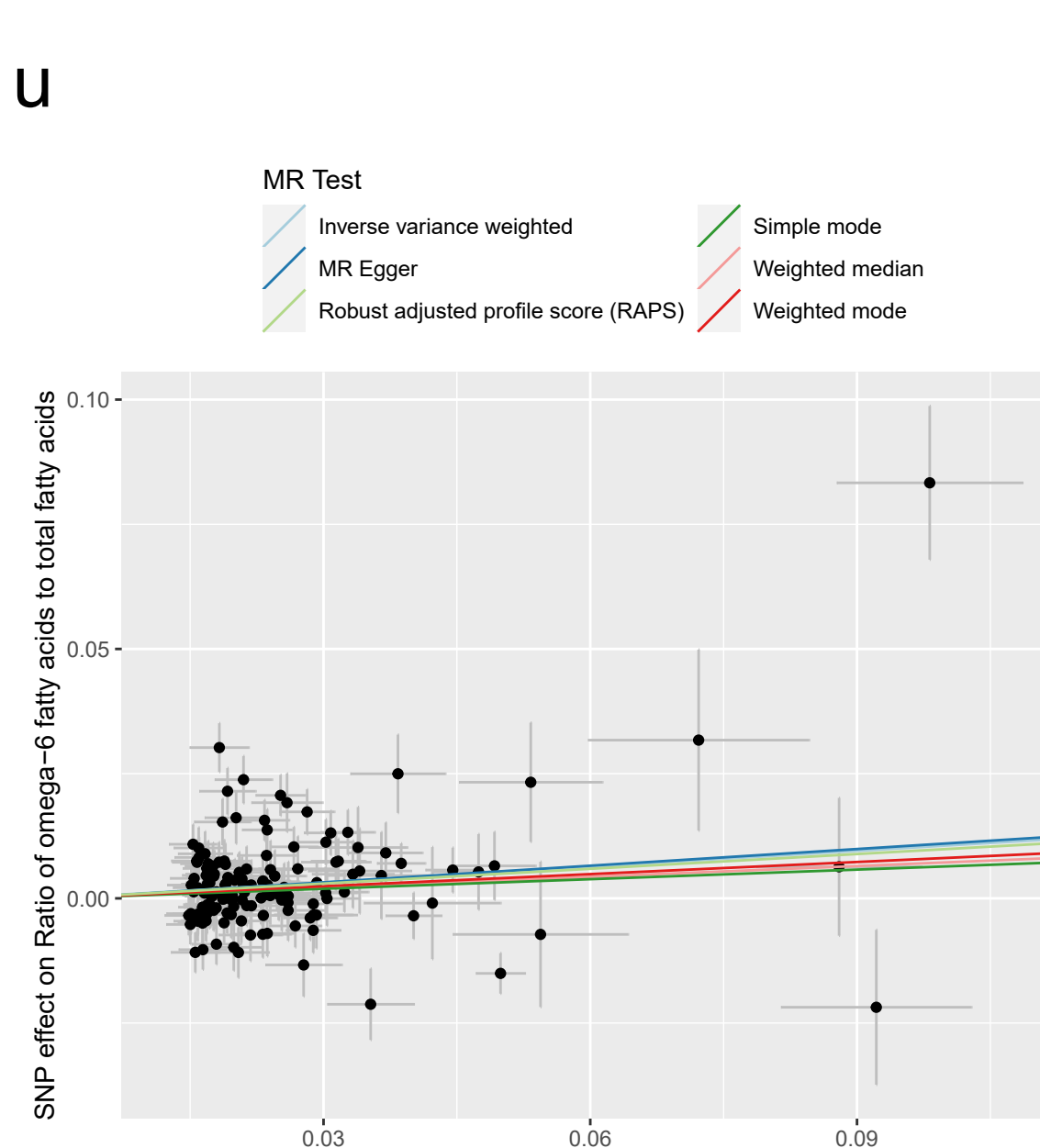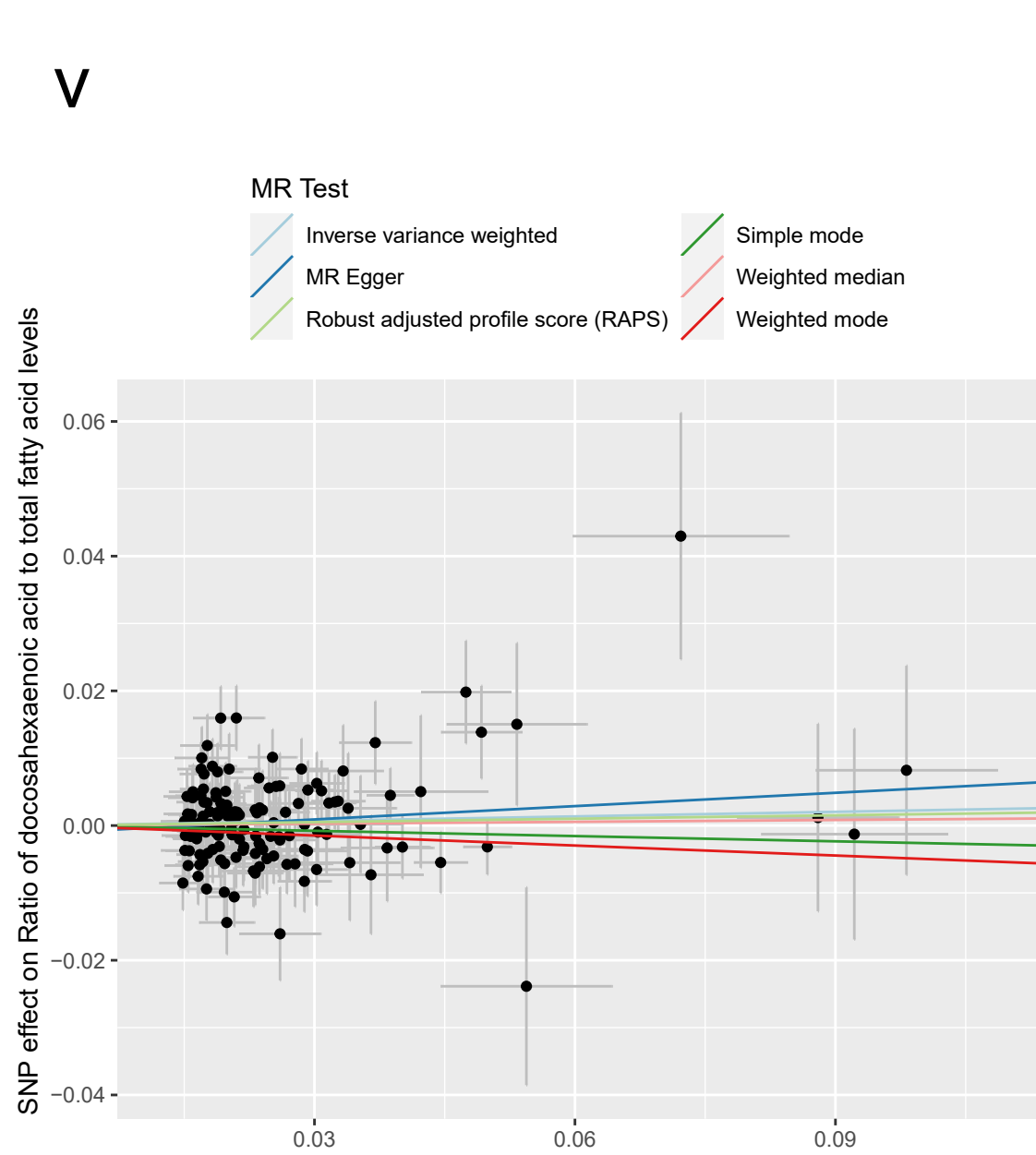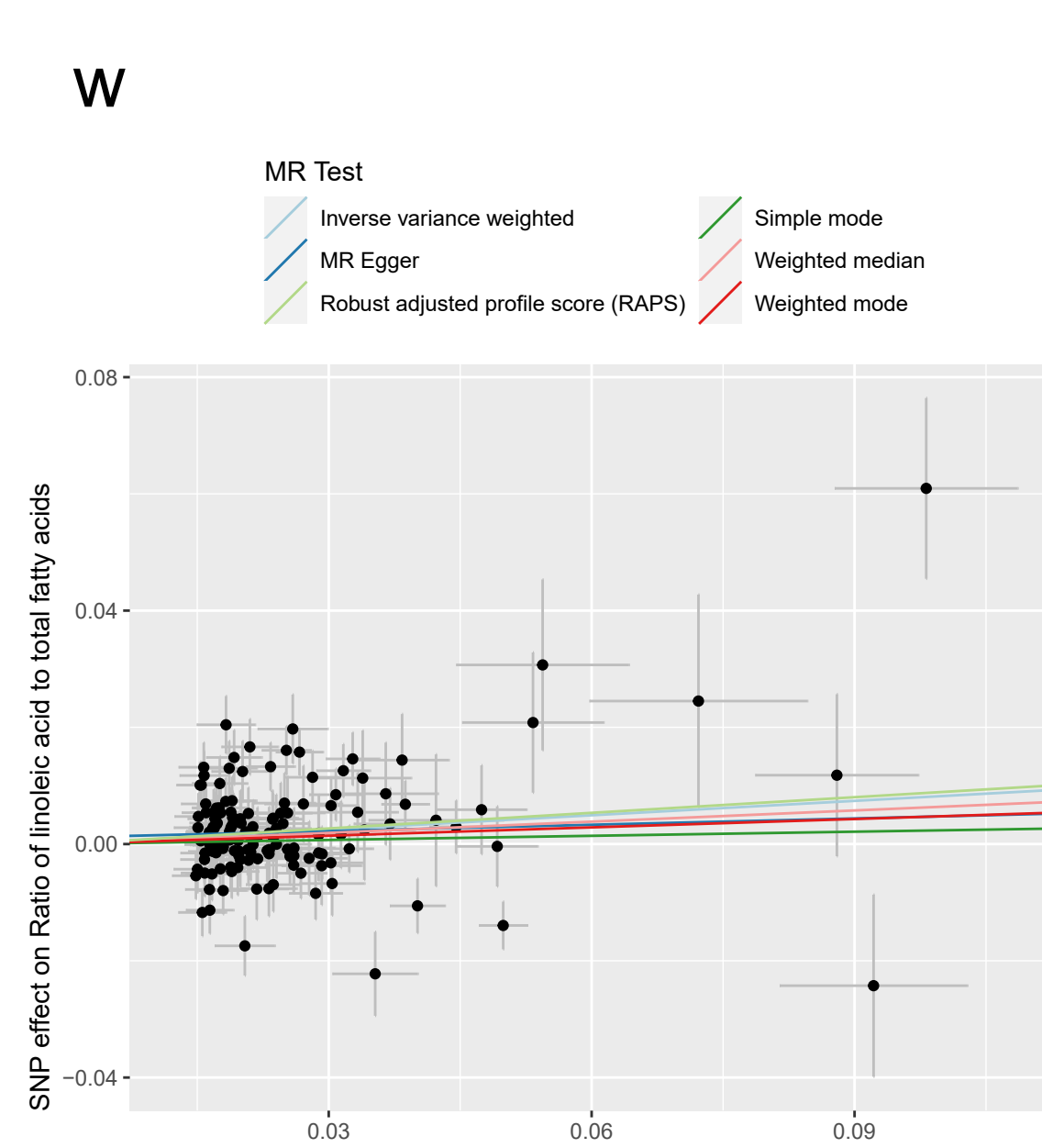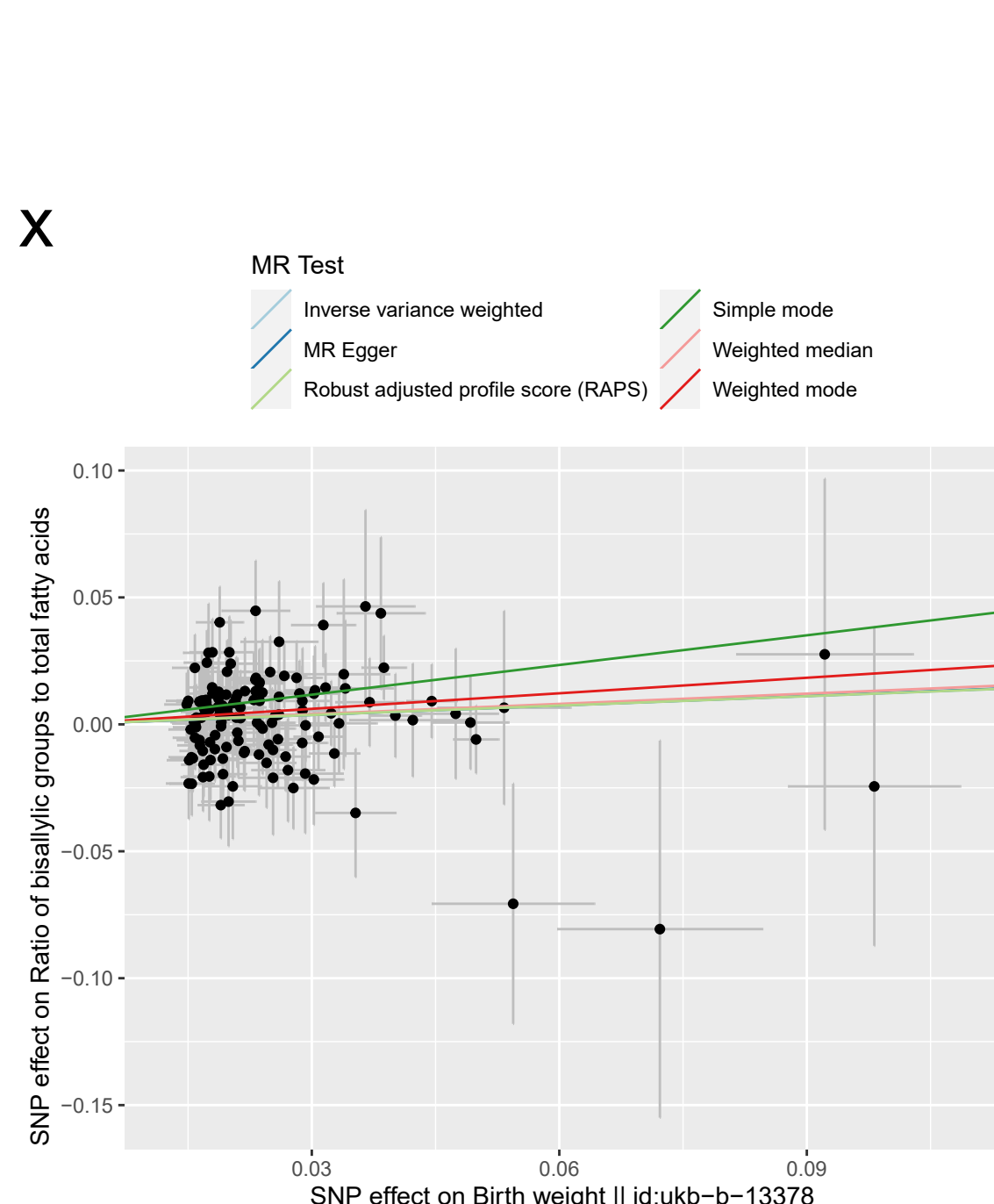

Supplement: Supplementary file 5 — Supplementary Material 5 [file 12944_2024_2087_MOESM5_ESM.pdf]

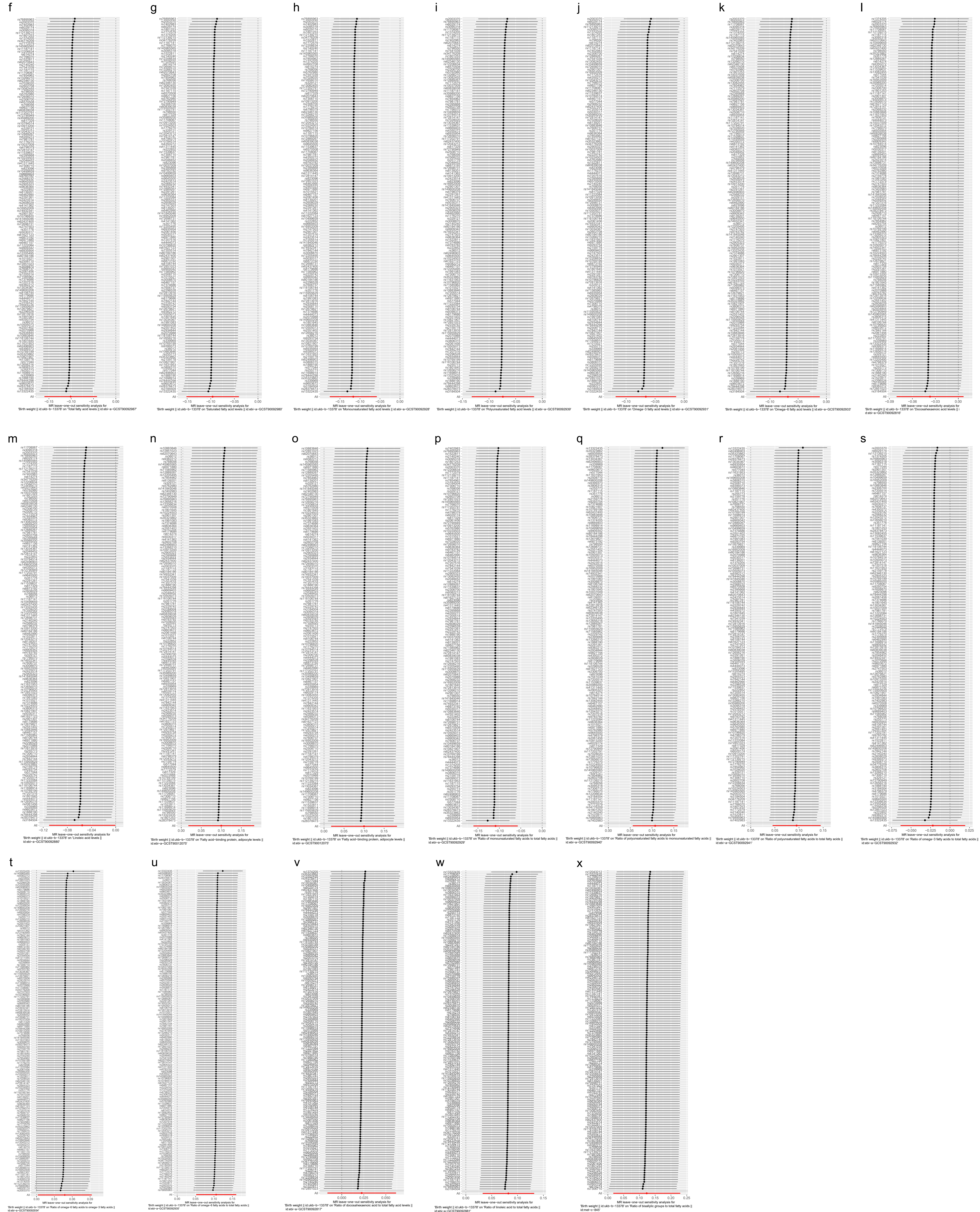

Supplement: Supplementary file 6 — Supplementary Material 6 [file 12944_2024_2087_MOESM6_ESM.pdf]
