# Supplementary material for: Association of birth weight with type 2 diabetes mellitus and the mediating role of fatty acids traits: a two-step mendelian randomization study
Source: Lipids Health Dis. 2024 Apr 2;23:97. doi: 10.1186/s12944-024-02087-z (PMC10986016; doi:10.1186/s12944-024-02087-z)
Supplement: Supplementary file 3 — Supplementary Material 3 [file 12944_2024_2087_MOESM3_ESM.docx]

Additional file 3

Supplemental text 1：Details of included GWASs

1.Birth weight

UK Biobank (UKB) is a renowned biomedical data repository that collects genetic and clinical data from half a million UK participants (1). SNPs associated with birth weight were obtained from UKB, which contained 261932 participants (261,932 cases and 9,851,867 controls).

2.T2DM and glycemic quantitative traits

The summary-level data on T2DM was obtained from the large multigene T2DM genome-wide association meta-analysis, comprising 48,286 cases and 250,671 controls, all from European ancestry (2). T2DM was diagnosed based on self-reported medical history and International classification of diseases (ICD) codes in linked electronic medical health records (2).

Additionally, glycemic quantitative traits, including fasting glucose, fasting insulin, HbA1c, and two-hour glucose were also collected as secondary outcomes. A GWAS study of fasting glucose, fasting insulin, and two-hour glucose included 200,622 (SNPs = 31,008,728), 151,013 (SNPs = 29,664,438), and 63,396 (SNPs = 27,330,879) participants of European ancestry, respectively (3). Summary statistics for HbA1c were acquired from a genome-wide meta-analysis that includes 45,734 participants from European ancestry (SNPs = 9,696,819) (4).

3.Fatty acids traits

We extracted summary-level data for the content of fatty acids, such as TFA, SFA, MUFA, PUFA, Omega-3 FA, Omega-6 FA, DHA, LA and A-FABP. Further, ratios of a series of fatty acids including SFA/TFA ratio, MUFA/TFA ratio, PUFA/MUFA ratio, PUFA/TFA ratio, Omega-3/TFA ratio, Omega-6/Omega-3 FA ratio, Omega-6/TFA ratio, DHA/TFA ratio, LA/TFA ratio, and bisallylic groups/TFA ratio, were obtained.

For A-FABP, summary data were extracted that involved 21,758 participants of European ancestry (SNPs = 13,138,563) (5). The GWAS for bisallylic groups/TFA ratio included 13,171 individuals (SNPs = 11,274,684) (6). Besides them, the rest of the fatty acids traits summary-level statistics were from one meta-analysis combining data that included 115,006 individuals of European ancestry (SNPs = 11,590,399)(7).

References

1. Bycroft C, Freeman C, Petkova D, Band G, Elliott LT, Sharp K, et al. The UK Biobank resource with deep phenotyping and genomic data. Nature. 2018;562(7726):203-9.

2. Mahajan A, Wessel J, Willems SM, Zhao W, Robertson NR, Chu AY, et al. Refining the accuracy of validated target identification through coding variant fine-mapping in type 2 diabetes. Nat Genet. 2018;50(4):559-71.

3. Chen J, Spracklen CN, Marenne G, Varshney A, Corbin LJ, Luan J, et al. The trans-ancestral genomic architecture of glycemic traits. Nat Genet. 2021;53(6):840-60.

4. Howe LJ, Nivard MG, Morris TT, Hansen AF, Rasheed H, Cho Y, et al. Within-sibship genome-wide association analyses decrease bias in estimates of direct genetic effects. Nat Genet. 2022;54(5):581-92.

5. Folkersen L, Gustafsson S, Wang Q, Hansen DH, Hedman AK, Schork A, et al. Genomic and drug target evaluation of 90 cardiovascular proteins in 30,931 individuals. Nat Metab. 2020;2(10):1135-48.

6. Kettunen J, Demirkan A, Wurtz P, Draisma HH, Haller T, Rawal R, et al. Genome-wide study for circulating metabolites identifies 62 loci and reveals novel systemic effects of LPA. Nat Commun. 2016;7:11122.

7. Richardson TG, Leyden GM, Wang Q, Bell JA, Elsworth B, Davey Smith G, et al. Characterising metabolomic signatures of lipid-modifying therapies through drug target mendelian randomisation. PLoS Biol. 2022;20(2):e3001547.

Supplemental text 2：The main code

#UVMR

bbb<-"1.ukb-b-13378→ebi-a-GCST007516"

bbb<-as.data.frame(bbb)

mtor<-extract_instruments(outcomes = "ukb-b-13378",p1=5e-8,clump=TRUE,r2=0.001,kb=10000,access_token= NULL)

try(mtor<-subset(mtor,eaf.exposure>=0.01) ,T)

try(mtor$r2<-2*(1-mtor$eaf.exposure)*(mtor$eaf.exposure)*(mtor$beta.exposure)^2,T)

try(mtor$F<- (mtor$r2/(1-mtor$r2))*(mtor$samplesize.exposure-2) ,T)

try(mtor<-subset(mtor,F>10) ,T)

try(F_all<-(sum(mtor$r2)/(1-sum(mtor$r2)))*(unique(mtor$samplesize.exposure)-length(mtor$samplesize.exposure)-1)/length(mtor$samplesize.exposure) ,T)

try(ccc<-bbb,T)

try(bbb<-as.data.frame(F_all) ,T)

try(ccc<-bind_rows(ccc,bbb) ,T)

try(t2d_out <- extract_outcome_data(snps=mtor$SNP,

outcomes='ebi-a-GCST007516',proxies = FALSE, maf_threshold = 0.01, access_token = NULL) ,T)

try(dat <- harmonise_data(exposure_dat=mtor,outcome_dat=t2d_out,action=2) ,T)

try(mr(dat,method_list = c("mr_ivw","mr_egger_regression","mr_weighted_median","mr_simple_mode","mr_weighted_mode","mr_raps")),T)

try(generate_odds_ratios(mr_res = mr(dat, method_list = c("mr_ivw","mr_egger_regression","mr_weighted_median","mr_simple_mode","mr_weighted_mode","mr_raps"))),T)

try(mr_result<-mr(dat,method_list = c("mr_ivw","mr_egger_regression","mr_weighted_median","mr_simple_mode","mr_weighted_mode","mr_raps")),T)

try(ccc<-bind_rows(ccc,as.data.frame(mr_result)[,c(5,7:9)]) ,T)

#Pleiotropy

try(pdf(file = "Pleiotropy.pdf",width=4, height=4) ,T)

try(mr_scatter_plot(mr_results = mr(dat,method_list = c("mr_ivw","mr_egger_regression","mr_weighted_median")),dat) ,T)

try(dev.off(),T)

try(mr_pleiotropy_test(dat) ,T)

try(bbb<-"Pleiotropy",T)

try(bbb<-as.data.frame(bbb) ,T)

try(ccc<-bind_rows(ccc,bbb) ,T)

try(ccc<-bind_rows(ccc,as.data.frame(mr_pleiotropy_test(dat))[,5:7]) ,T)

#Heterogeneity

try(pdf(file = "Heterogeneity.pdf",width=4, height=4) ,T)

try(mr_funnel_plot(singlesnp_results = mr_singlesnp(dat)) ,T)

try(dev.off(),T)

try(bbb<-"Heterogeneity",T)

try(bbb<-as.data.frame(bbb) ,T)

try(ccc<-bind_rows(ccc,bbb) ,T)

try(ccc<-bind_rows(ccc,mr_heterogeneity(dat)[,6:8]) ,T)

#leaveoneout

try(pdf(file = "leaveoneout.pdf",width=4, height=4) ,T)

try(mr_leaveoneout_plot(leaveoneout_results = mr_leaveoneout(dat)) ,T)

try(dev.off(),T)

try(write.xlsx(ccc,file = "Result.xlsx"),T)

try(ddd<-bind_rows(ddd,ccc) ,T)

#MVMR

bbb<-"1+ebi-a-GCST90092987+ebi-a-GCST007516"

bbb<-as.data.frame(bbb)

id_exposure<-c("ukb-b-13378","ebi-a-GCST90092987")

try(exposure_dat<-mv_extract_exposures(id_exposure, pval_threshold=5e-8, clump_r2=0.001,clump_kb=10000,access_token=NULL) ,T)

try(id_outcome <- "ebi-a-GCST007516" ,T)

try(outcome_dat <- extract_outcome_data(exposure_dat$SNP, id_outcome) ,T)

try(mvdat <- mv_harmonise_data(exposure_dat, outcome_dat) ,T)

try(res <- mv_multiple(mvdat, pval_threshold = 1) ,T)

try(ccc3<-bbb,T)

try(ccc3<-bind_rows(ccc3,as.data.frame(res)[,c(2,5:8)]),T)

try(write.xlsx(ccc3,file = "1+ebi-a-GCST90092987+ebi-a-GCST007516.xlsx"),T)

try(eee<-bind_rows(eee,ccc3) ,T)

try(ddd<-bind_rows(ddd,ccc3) ,T)

#BH

Data<- read.xlsx("input.xlsx", sheet = 2)

Data$BH =

p.adjust(Data$Raw.p,

method = "BH")

Supplemental text 3：STROBE-MR checklist of recommended items to address in reports of Mendelian randomization studies

| **Item No.** | **Section** | **Checklist item** | **Page No.** | **Relevant text from manuscript** |
| --- | --- | --- | --- | --- |
| 1 | **TITLE and ABSTRACT** | Indicate Mendelian randomization (MR) as the study’s design in the title and/or the abstract if that is a main purpose of the study | 1,2 | The term "Mendelian randomization" was incorporated into both the title and abstract. |
|  | **INTRODUCTION** |  |  |  |
| 2 | **Background** | Explain the scientific background and rationale for the reported study. What is the exposure? Is a potential causal relationship between exposure and outcome plausible? Justify why MR is a helpful method to address the study question | 3-6 | Introduction, Line 55-92. |
| 3 | **Objectives** | State specific objectives clearly, including pre-specified causal hypotheses (if any). State that MR is a method that, under specific assumptions, intends to estimate causal effects | 6 | Introduction, Line 92-94. |
|  | **METHODS** |  |  |  |
| 4 | **Study design and data sources** | Present key elements of the study design early in the article. Consider including a table listing sources of data for all phases of the study. For each data source contributing to the analysis, describe the following: |  |  |
|  | a) | Setting: Describe the study design and the underlying population, if possible. Describe the setting, locations, and relevant dates, including periods of recruitment, exposure, follow-up, and data collection, when available. | 6-8, | Methods, Line 113-135;  Figure 2. |
|  | b) | Participants: Give the eligibility criteria, and the sources and methods of selection of participants. Report the sample size, and whether any power or sample size calculations were carried out prior to the main analysis | 8,9 | Methods, Line 128-135;  Table1;  Supplemental text 1. |
|  | c) | Describe measurement, quality control and selection of genetic variants | 10 | Methods, Line 140-153. |
|  | d) | For each exposure, outcome, and other relevant variables, describe methods of assessment and diagnostic criteria for diseases |  | Supplemental text 1. |
|  | e) | Provide details of ethics committee approval and participant informed consent, if relevant | 8 | Methods, Line 125-126. |
| 5 | **Assumptions** | Explicitly state the three core IV assumptions for the main analysis (relevance, independence and exclusion restriction) as well assumptions for any additional or sensitivity analysis | 7 | Methods, Line 117-121. |
| 6 | **Statistical methods: main analysis** | Describe statistical methods and statistics used |  |  |
|  | a) | Describe how quantitative variables were handled in the analyses (i.e., scale, units, model) | N/A | In our MR analysis, the exposure (i.e., birth weight) had a unit of 1-SD and was standardized in its GWAS. For fasting insulin, fasting glucose, HbA1c, and two-hour glucose, the units were log-transformed pmol/L, mmol/L, %, and log-transformed mmol/L. |
|  | b) | Describe how genetic variants were handled in the analyses and, if applicable, how their weights were selected | 10,11 | Methods, Line 140-153;  Line 172-181. |
|  | c) | Describe the MR estimator (e.g. two-stage least squares, Wald ratio) and related statistics. Detail the included covariates and, in case of two-sample MR, whether the same covariate set was used for adjustment in the two samples | 10-12 | Methods, Line 154-181. |
|  | d) | Explain how missing data were addressed |  | N/A |
|  | e) | If applicable, indicate how multiple testing was addressed | 12 | Methods, Line 182-183. |
| 7 | **Assessment of assumptions** | Describe any methods or prior knowledge used to assess the assumptions or justify their validity | 10,11 | Methods, Line 140-153;  Line 172-181. |
| 8 | **Sensitivity analyses and additional analyses** | Describe any sensitivity analyses or additional analyses performed (e.g. comparison of effect estimates from different approaches, independent replication, bias analytic techniques, validation of instruments, simulations) | 11 | Methods, Line 172-181. |
| 9 | **Software and pre-registration** |  |  |  |
|  | a) | Name statistical software and package(s), including version and settings used | 12 | Methods, Line 183-185. |
|  | b) | State whether the study protocol and details were pre-registered (as well as when and where) | N/A |  |
|  | **RESULTS** |  |  |  |
| 10 | **Descriptive data** |  |  |  |
|  | a) | Report the numbers of individuals at each stage of included studies and reasons for exclusion. Consider use of a flow diagram | N/A |  |
|  | b) | Report summary statistics for phenotypic exposure(s), outcome(s), and other relevant variables (e.g. means, SDs, proportions) | N/A |  |
|  | c) | If the data sources include meta-analyses of previous studies, provide the assessments of heterogeneity across these studies | N/A |  |
|  | d) | For two-sample MR:  i.  Provide justification of the similarity of the genetic variant-exposure associations between the exposure and outcome samples  ii.  Provide information on the number of individuals who overlap between the exposure and outcome studies | N/A | There was minimal ethic heterogeneity because all the samples used here were of European ancestry. However, the study did not report any information regarding overlapping populations between the exposure and outcome in our study. |
| 11 | **Main results** |  |  |  |
|  | a) | Report the associations between genetic variant and exposure, and between genetic variant and outcome, preferably on an interpretable scale | 12-20 |  |
|  | b) | Report MR estimates of the relationship between exposure and outcome, and the measures of uncertainty from the MR analysis, on an interpretable scale, such as odds ratio or relative risk per SD difference | 12-20 |  |
|  | c) | If relevant, consider translating estimates of relative risk into absolute risk for a meaningful time period | N/A |  |
|  | d) | Consider plots to visualize results (e.g. forest plot, scatterplot of associations between genetic variants and outcome versus between genetic variants and exposure) | 14,16,18 | Figures 3-5 and Figure S2-S4. |
| 12 | **Assessment of assumptions** |  |  |  |
|  | a) | Report the assessment of the validity of the assumptions |  | Table S14-16 |
|  | b) | Report any additional statistics (e.g., assessments of heterogeneity across genetic variants, such as *I^2^*, Q statistic or E-value) |  | Table S14-16 |
| 13 | **Sensitivity analyses and additional analyses** |  |  |  |
|  | a) | Report any sensitivity analyses to assess the robustness of the main results to violations of the assumptions |  | Figure 3 and Table S10-12. |
|  | b) | Report results from other sensitivity analyses or additional analyses |  | Figure 3 and Table S10-12. |
|  | c) | Report any assessment of direction of causal relationship (e.g., bidirectional MR) | 15 | Line 219-221. |
|  | d) | When relevant, report and compare with estimates from non-MR analyses | 21-26 | Discussion section. |
|  | e) | Consider additional plots to visualize results (e.g., leave-one-out analyses) |  | Figure S6 |
|  | **DISCUSSION** |  |  |  |
| 14 | **Key results** | Summarize key results with reference to study objectives | 20,21 | Discussion, Line 274-285. |
| 15 | **Limitations** | Discuss limitations of the study, taking into account the validity of the IV assumptions, other sources of potential bias, and imprecision. Discuss both direction and magnitude of any potential bias and any efforts to address them | 25,26 | Discussion, Line 383-400. |
| 16 | **Interpretation** |  |  |  |
|  | a) | Meaning: Give a cautious overall interpretation of results in the context of their limitations and in comparison with other studies | 21-25 | Discussion, Line 286-382. |
|  | b) | Mechanism: Discuss underlying biological mechanisms that could drive a potential causal relationship between the investigated exposure and the outcome, and whether the gene-environment equivalence assumption is reasonable. Use causal language carefully, clarifying that IV estimates may provide causal effects only under certain assumptions | 21-25 | Discussion, Line 286-382. |
|  | c) | Clinical relevance: Discuss whether the results have clinical or public policy relevance, and to what extent they inform effect sizes of possible interventions | 21-25 | Discussion, Line 286-382. |
| 17 | **Generalizability** | Discuss the generalizability of the study results (a) to other populations, (b) across other exposure periods/timings, and (c) across other levels of exposure | 25 | Discussion, Line 392-394. |
|  | **OTHER INFORMATION** |  |  |  |
| 18 | **Funding** | Describe sources of funding and the role of funders in the present study and, if applicable, sources of funding for the databases and original study or studies on which the present study is based | 28 | Funding, Line 419-423. |
| 19 | **Data and data sharing** | Provide the data used to perform all analyses or report where and how the data can be accessed, and reference these sources in the article. Provide the statistical code needed to reproduce the results in the article, or report whether the code is publicly accessible and if so, where | 29 | Availability of data and materials, Line 424-427. |
| 20 | **Conflicts of Interest** | All authors should declare all potential conflicts of interest | 29 | Competing Interests, Line 434-435. |

This checklist is copyrighted by the Equator Network under the Creative Commons Attribution 3.0 Unported (CC BY 3.0) license.
